# The Banana Genome Hub

**DOI:** 10.1093/database/bat035

**Published:** 2013-05-23

**Authors:** Gaëtan Droc, Delphine Larivière, Valentin Guignon, Nabila Yahiaoui, Dominique This, Olivier Garsmeur, Alexis Dereeper, Chantal Hamelin, Xavier Argout, Jean-François Dufayard, Juliette Lengelle, Franc-Christophe Baurens, Alberto Cenci, Bertrand Pitollat, Angélique D’Hont, Manuel Ruiz, Mathieu Rouard, Stéphanie Bocs

**Affiliations:** ^1^CIRAD, UMR AGAP, Montpellier F-34398, France, ^2^Montpellier SupAgro, UMR AGAP, Montpellier F-34060, France, ^3^Bioversity International, Commodity Systems & Genetic Resources Programme, Montpellier F-34397, France and ^4^IRD, UMR RPB, Montpellier F-34394, France

## Abstract

Banana is one of the world’s favorite fruits and one of the most important crops for developing countries. The banana reference genome sequence (*Musa acuminata*) was recently released. Given the taxonomic position of *Musa*, the completed genomic sequence has particular comparative value to provide fresh insights about the evolution of the monocotyledons. The study of the banana genome has been enhanced by a number of tools and resources that allows harnessing its sequence. First, we set up essential tools such as a Community Annotation System, phylogenomics resources and metabolic pathways. Then, to support post-genomic efforts, we improved banana existing systems (e.g. web front end, query builder), we integrated available *Musa* data into generic systems (e.g. markers and genetic maps, synteny blocks), we have made interoperable with the banana hub, other existing systems containing *Musa* data (e.g. transcriptomics, rice reference genome, workflow manager) and finally, we generated new results from sequence analyses (e.g. SNP and polymorphism analysis). Several uses cases illustrate how the Banana Genome Hub can be used to study gene families. Overall, with this collaborative effort, we discuss the importance of the interoperability toward data integration between existing information systems.

**Database URL**: http://banana-genome.cirad.fr/

## Introduction

We recently published a reference genome sequence for banana (1). Banana is a tropical crop of socio-economic interest, as it is a staple food in developing countries, producing biomass of sugar, starch and cellulose (used for paper, textiles and fuel). In addition to its socio-economic importance, banana is the first non-*Poaceae* (grass family) monocotyledon for which a high-continuity whole-genome sequence is available, representing an essential bridge for comparative genome analysis in plants. The 472 Mb sequenced assembly was generated from DH-Pahang, a doubled-haploid genotype, obtained from the *Musa acuminata* subspecies *malaccensis* accession ‘Pahang’ (523Mb 1C estimated size). Several analytical pipelines were applied for gene (2), transposable element (TE) (3), expression data (4) and comparative genomics (5) to the analysis of the *Musa* genome. We stored the resulting data such as *ab initio* gene predictions, repeat elements, Expressed Sequence Tag (EST)/RNAseq assemblies, SNP markers, plant polypeptides clusters and orthologous relationships in various databases accessible through a bioinformatics Platform called South Green (6).

We anticipate that the number of tools used, and the quantity of data available, will continue to grow owing to the increase of NGS-based projects (Next Generation Sequencing). Thus, to build a dynamic and sustainable working environment, we developed a public crop-specific hub for *Musa* genomic information, described in this article. Our global strategy in implementing this hub was to exploit, whenever possible, generic software solutions interconnected to establish a reliable framework for scientists interested in banana and related biology. Similar specialized plant hubs exist, such as Gramene for cereal genomes (7), the Sol genomics network for the tomato genome sequencing project (8) and resources like the plant section of Ensembl (9). However, few such plant information hubs have the following complete integrated functionalities:

- A Chado-based (10, 11) community annotation system (CAS)
- Connectivity to a mainstream genome annotation editor (12)
- Editorial oversight by a Controller for history revision, data quality and permission management (11)
- Interoperability with Tripal (13), Galaxy(14) and several published tools for plant genomics

## The Hub content

The Banana Genome Hub is based on Tripal—a construction toolkit for online genome databases—to facilitate the integration between various systems that we developed for plant genome analysis that includes several major banana data sets. The Banana Genome Hub is supported by the South Green Bioinformatics Platform (http://southgreen.cirad.fr/), which gives access to original bioinformatics methods and tools to manage genetic and genomic resources of tropical plants that are summarized in Supplementary Table S1.

### Structural and functional genome annotations

Errors and deficiencies in genome annotation recorded in public databases is an ongoing global research community concern. Systematic activities for community annotation of the sequenced genomes of each genus can enhance the accuracy of such annotation. For example, scientific domain experts and students can collaborate with genome community information resource curators to review the annotation of genes or gene families of interest in jamborees (15). But to generate a larger volume of high-quality genomic annotations within a dispersed heterogeneous community, an operational high-quality and user-friendly ‘crowd sourcing’ on-line annotation system is desirable.

**Figure 1.**
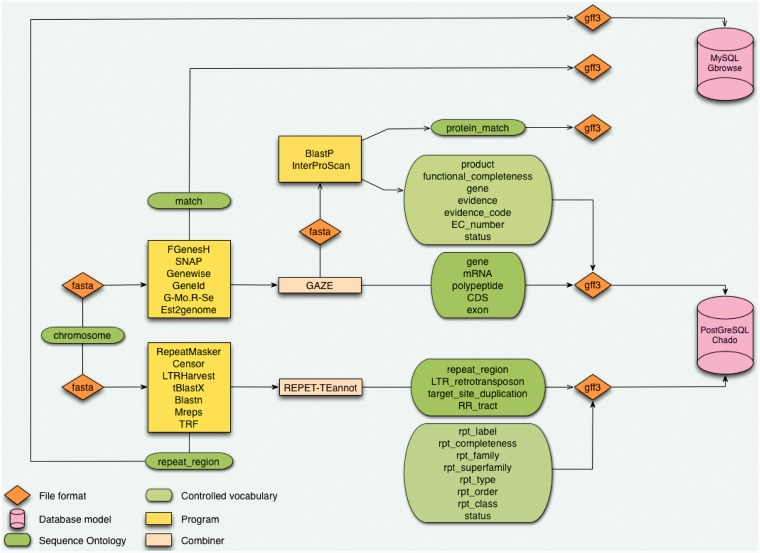
Architecture of the CAS. The starting point is a sequence without annotation which is being processed in analyses pipeline for genes and repeat elements annotation. Results are structured with the Sequence Ontology and controlled vocabularies. Data are formatted in GFF3 before being inserted in databases using Perl loaders.

To support this objective, we deploy a CAS (Figure 1) (16), which is the result of collaborative research to develop a generic, modular and sustainable system broadly applicable to eukaryotic organisms such as plants, insects (17) or fungi (18). In particular, a CAS called GNPAnnot is dedicated to the annotation of Mediterranean and tropical plant genomic sequences. Our Banana Hub is centered on a CAS deployed for the ongoing automated and manual annotation of the *Musa* DH-Pahang genome. These CAS installations use popular interoperable open source software, such as components of the Generic Model Organism Database project (GMOD) (19) to achieve their aims. CAS annotation workflows can connect many programs and are fine-tuned according to the particular genomic project.

In the Banana Genome Hub, the data flow starts with a DH-Pahang assembly composed of 11 pseudomolecules corresponding to the 11 *Musa* chromosomes and an additional pseudomolecule resulting from a random concatenation of contigs not yet assigned to a specific chromosome (Table 1). The protein-coding genes were predicted by Genoscope using the GAZE combiner (2), whereas we performed the TE prediction with the REPET package (3) (Figure 1). GAZE is an integrative gene finding software that combines several evidence such as *ab initio* predictions (Geneid, SNAP or FGenesH) (20–22), the mapping of different protein sequence sets (Genewise) (23), ESTs and full-length cDNAs (Est2genome) (24), as well as RNA-Seq reads from Solexa/Illumina technology (Gmorse) (25). The outputs of these analyses are formatted in fully compliant Generic Feature Format (GFF3) files most commonly used as the exchange format between components.

**Table 1.** Statistics of annotated genes and TEs in the banana genome through GNPAnnot (as of 29 November 2012). ChrUn_random corresponds to un-anchored scaffolds

Chromosome	Length (bp)	Gene count		TE count	
		Predicted	Curated	Predicted	Curated
chr1	27 573 629	2834	29	9259	0
chr2	22 054 697	2327	23	6748	0
chr3	30 470 407	3253	51	9188	0
chr4	30 051 516	3367	43	9416	11
chr5	29 377 369	2974	44	9727	0
chr6	34 899 179	3700	61	11 129	5
chr7	28 617 404	2766	38	8931	0
chr8	35 439 739	3454	48	11 758	0
chr9	34 148 863	3105	65	11 716	1
chr10	33 665 772	3157	54	11 013	0
chr11	25 514 024	2678	32	8833	0
chrUn_random	141 147 818	2927	0	64 340	0
Total	472 960 417	36 542	488	172 058	17

GAZE analysis, validated by some manual expert curation, predicted a consensus annotation of 36 542 protein-encoding genes. To define canonical protein-coding gene models, annotation was aggregated into gene entries consisting of one or more exons, one or more mRNAs and at least one polypeptide. Following the structural gene prediction step, we performed similarity searches (BLASTP) against UniProtKB/SwissProt, UniProtKB/TrEMBL and the rice proteome database, plus domain searches with InterProScan (26) to infer functions for each protein-coding genes model. We defined three parameters: (i) qcov (the percentage of the query covered in the match), (ii) scov (the percentage of the subject covered in the match) and (iii) percentage identity (the percentage amino acid identity in the match). We kept the best ranking following a decision tree to transfer the polypeptide function [e.g. Sucrose-phosphate synthase (SPS)] with a confidence level (close to the Gene Ontology Evidence Code) (27) and to define the completeness of the corresponding gene. All the predicted functions we found at this step contributed to create an in-house controlled vocabulary, namely, product. Other controlled vocabularies were also applied, such as gene (e.g. SPS), Enzyme Commission (EC)_number (e.g. 2.4.1.14), completeness (e.g. complete), is_obsolete (e.g. no), status (e.g. in_progress), evidence code (e.g. Inferred by Curator (IC)), to disambiguate the term used in gene annotations and to improve the data quality (11). The results of InterProScan were used to assign Gene Ontology (GO) terms with InterPro2GO. These resources contributed to enrich the GFF3 files dedicated to the manual annotation, at the polypeptide level, with cross-references on external databases (Dbxref), Gene Ontology terms and controlled vocabulary (Ontology_term).

CAS functionality has already demonstrated its usefulness in several annotators’ training sessions. Such CAS were applied to the preparation of several feature sets [learning gene set, Long Terminal Repeat (LTR) retrotransposon library (1)], and supported genomic studies (28–30).

### Gene families

For a newly sequenced genome, such as *Musa acuminata*, comparative genomics is critical for the generation of reliable gene function annotations. The Banana Genome Hub relies on a robust comparative genomics database called GreenPhylDB (31), which now includes the *Musa* protein-coding genes. The current version of GreenPhylDB compiles a stable and curated catalog of protein gene families based on the automatic clustering of 22 whole plant genome sequences. Specific information related to the clustering of the *Musa* genome is reported in Table 2. This information is calibrated against the 22 plant genomes and provides a suitable framework for evolutionary and functional analyses. The banana genome exhibits an interesting evolutionary pattern with several rounds of ancestral whole-genome duplications (WGD). Specific interfaces are available in GreenPhylDB to compare protein domains (http://www.greenphyl.org/cgi-bin/ipr2genomes.cgi) and to support analysis of the distribution of transcriptions factors across plant genomes. We established cross-links to the genome browser to navigate from gene families to individual genes inside the Banana Genome Hub.

**Table 2.** GreenPhylDB statistics associated with clusters containing *Musa* sequences. Half of the clusters at level 1 are actually curated (as of 29 November 2012)

Total number of clusters (Level1)	Sequences in clusters	Curated clusters	Sequences with InterPro signatures	Gene trees available	Number of homologous relationships
4686	31 192 (85%)	2855 (∼50%)	26 523	3000	329 546 orthologs
					(15 571 arabidopis thaliana)
					(13 591 Oryza sativa)
					11 842 ultraparlogs / in-paralogs

### Paralogous relationships within the banana genome

**Figure 2.**
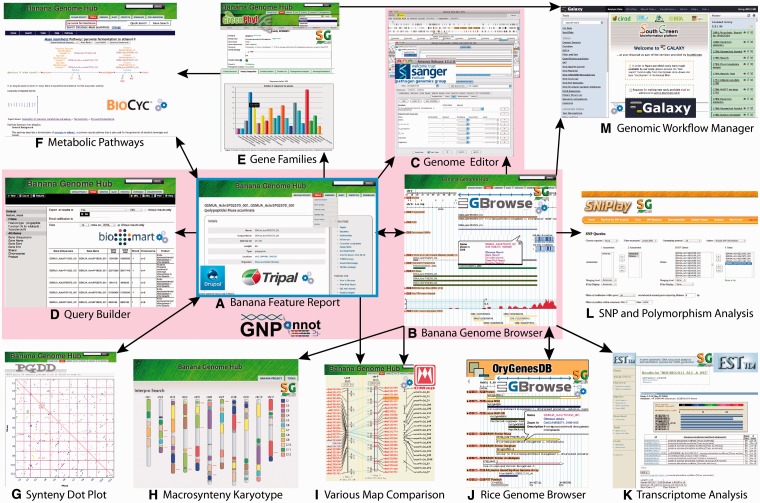
Interoperability within the Banana Genome Hub. The main entry point for the Banana Genome Hub (blue frame) is the Drupal CMS that has Tripal modules, the Web front end and gene report for Chado database. The Hub relies on URL integration of resources using a common uniquename (Chado feature table) and semantic terms (e.g. ontology). In the Chado schema, unique identifiers correspond to the column ‘uniquename’ (e.g. GSMUA_Achr4G16070_001). The same unique identifiers are stored in the other databases (e.g. GreenPhylDB, SNiPlay, MusaCyc, GBrowse, Tripal), and the links are based on this uniquename used for the polypeptide. The same concept applies for others types like genetic markers. The arrows indicate direct links between them. The GNPAnnot CAS [Tripal (**A**), GBrowse (**B**), Artemis (**C**), BioMart (**D**)] composes the core of the Banana Genome Hub (pink zone). All other bioinformatic systems are integrated using HTML iframes (those with the Banana Genome Hub green banner) such as GreenPhylDB (**E**), MusaCyc (**F**), PGDD Dot Plot (**G**), Macrosynteny Karyotype (**H**) CMAP and TropGeneDB (**I**), SNiPlay (**J**) and Galaxy (**M**). Banana Genome Browser links also ESTtik (**K**) and OryGenesDB (**L**). The in-house Advanced Search is linked to the GBrowse 2. Biomart query builder allows exporting personalized qualifiers of genomic features in various formats. The Macrosynteny Karyotype is linked to GBrowse 2 using the Bio::DB::SeqFeature::Store MySQL database. CMAP allows the Comparison of various maps (sequence, genetic, etc.). (**G**) The system of the PGDD is used to show the Beta ancestral blocks reconstruct from the DH-Pahang paralogous regions. (**H**) The Macrosynteny Karyotype is the result of an Advanced Search. It allows mapping the querying features relatively to the Beta ancestral blocks.

Any search within the Banana Genome Hub will display the matching genomic features on the *Musa* chromosomes (Figure 2) where paralogous regions are colored according to beta ancestral blocks as defined by D’Hont *et al.* (1). A local version of Plant Genome Duplication Database (PGDD) (32) was implemented with a dynamic dot plot allowing to focus on syntenic regions and provide access to the list of the duplicated gene pairs. Duplicated regions were detected with SynMap program of the CoGe web site (33) using a quota-align ratio of 3:3 (33). The synonymous substitution rate (Ks) and the non-synonymous one (Ka) of each gene pair were calculated using the Yang–Nielson method (34).

### Metabolic pathways

We set up a pathway tools database (35) for metabolic pathways that we called MusaCyc. Banana genes coding for enzymes were classified following the EC number using PRIAM (*PRofils pour l'Identification Automatique du Métabolisme*) (36). Then, metabolic pathways were predicted from these EC numbers using Pathway Tools (35). We also used the Pathway Hole filler program to identify missing enzymes in the MusaCyc database. The percentage of MusaCyc enzymes and transporters predicted from the proteome was 17.1% (6128 enzymes and 112 transporters for a total of 36 528 polypeptides), against 13.9% for RiceCyc v3.3 (6040 enzymes and 603 transporters for a total of 47 894) and 22.5% for AraCyc v8.0 (6023 enzymes and 143 transporters for a total of 27 416).

### Transcriptomics

Transcriptomics data and ESTs data are useful resources to characterize transcription patterns and to validate gene structures (37). The ESTtik system contains a semi-automatic cDNA annotation pipeline and a database, providing public and private access to cDNA libraries, including Next Generation Sequencing data. We used the system to annotate 14 banana cDNA libraries, comprising five genotypes, and various tissues and conditions. The transcriptome database records 91 041 public banana cDNA sequences available through the Global *Musa* Genomics Consortium, assembly data and various annotation data (e.g. Blast against public databases, Gene Ontology data, domain search, SSR and SNP data mining). We designed specialized web interfaces to query and visualize all the information. These sequences were linked and mapped onto the assembled genome in a two-step procedure (1): Blast and then a refinement with Est2Genome software (24). The results are available within the *Musa* GBrowse 2 on a track called ‘MusaGenomics consortium ESTs’. Similarly, Genoscope aligned 6 888 879 EMBL monocotyledon mRNAs with Blat and Est2Genome (GBrowse Public monocotyledon EST track). cDNA reads (829 587 from DH-Pahang) produced by 454 technology were aligned to the banana sequence assembly with the same procedure and posted to GBrowse Pahang 454 EST read track.

Approximately 30 million Illumina sequence reads were obtained for each of the four RNA-Seq libraries corresponding to cDNA libraries from DH-Pahang and Pahang genotypes inoculated with fungal pathogens and the corresponding controls. We mapped the reads to the genome using the BWA aligner (38) converting SAM to BAM format using samtools (39). The Bio::DB::SAM adaptor allows the Genome browser to render these binary file as xyplot plots. The number of available *Musa* RNA-Seq libraries is expected to increase rapidly in the near future.

### SNP and polymorphism analysis

We mined putative SNPs from the mapping of RNA-Seq data originating from the two genotypes (Pahang and DH-Pahang) against predicted coding sequences and stored them in the SNiPlay database (40). Overall, among 10 266 genes exceeding a 10× depth threshold for at least one genotype and computationally mined for SNPs, we identified 3311 sequence variants in 1350 different genes (partial data). The SNiPlay database enables users to discriminate between intra-genotype and inter-genotype SNP. Thus, 2689 intra-genotype SNPs are predicted in *Musa acuminata* Pahang corresponding to a density of 1 SNP every 1394 bp. As expected, the detected heterozygosity is low in double haploid Pahang compared to its parent Pahang (only 83 intra-genotypic SNP). Finally, 1245 SNPs (∼37%) appear to be non-synonymous coding versus 2060 (∼63%) synonymous coding SNPs. SNiPlay offers a searchable web interface where all SNPs can be queried using different criteria. Moreover, the possibility to export data in GFF format has enabled the SNPs to be integrated into the GBrowse database and thus to be visualized through the genome browser. Data entries in both SNiPlay and GBrowse are cross-linked.

### Other molecular markers, genetic maps and genetic resources

Molecular markers (SSR, DArT) and genetic maps of banana are stored in the TropGeneDB (41). This information system, organized on a crop basis with currently nine public modules including banana and other tropical plants, records molecular markers, quantitative trait loci, genetic and physical maps, genetic diversity and phenotypic diversity studies, as well as a short description of genetic resources related to the so-called passport data (geographic origin, parentage, collections). Crop-specific web interfaces allow complex queries, the results being related to the CMAP viewer (the Comparative Map Viewer) and the GBrowse. Six genetic maps are currently recorded in the banana module, as well as one genotyping study, and microsatellites data on 541 germplasm samples covering a wide range of *Musa* genetic diversity (42). The Pahang genetic map contains 652 markers on 11 *Musa* linkage groups and spans 1477 cM (1). Banana germplasm used in genotyping studies are identified with a unique identifier linked to passport data and morphological data documented in the *Musa* Germplasm Information System (MGIS; http://www.crop-diversity.org/banana/). From MGIS, *in vitro* Banana germplasm can be ordered at the International banana genebank for further studies (Figure 1). More than 1200 *Musa* accessions, representing much of the diversity of the crop, are maintained in the genebank at International Transit Centre (ITC) of Bioversity International in Belgium.

### Links with genome resources in Rice, the model plant for monocotyledons

Rice is one of the most studied crop genomes and is a model plant for monocotyledons. As banana belongs to the Zingiberales, a sister order of the Poales, a strong link with rice genome resources is extremely useful. We connected the Banana Genome Hub to OryGenesDB (43), an interactive tool for rice reverse genetics. OryGenesDB contains >245 000 flanking sequence tags of various mutagens and functional genomics data collected from both international insertion collections and the literature. OryGenesDB provides a set of tools around GMOD Genome Browser to visualize or to search for insertions in candidate genes. In all, 11 665 putative pairs of orthologs between rice and banana have been identified using reciprocal blast hit strategy, and it is possible to take advantage of the system for gene annotation or functional genomics purposes. Various research tools implemented on OryGenesDB were adapted to the Banana Genome Hub. These allow end users to search by keywords, locus, InterPro domain, EC number, location or by Gene Ontology identifier. The results are displayed as a dynamic table that summarizes information on the corresponding locus and can be sent directly to Galaxy, where other data analysis workflows can be designed and performed.

## Data Aggregation and System Interoperability

### Data storage

We use the modular relational database schema Chado to store only the gene models (e.g. GAZE predictions) and functional analysis results (Blastp and InterPro) required for manual annotation activities, in a PostgreSQL database. All other GFF3 features comprising structural annotations (i.e. FGenesH, Geneid, Genewise outputs) are stored in a separate GBrowse MySQL database.

Manual annotation activities are performed using the Artemis annotation tool directly connected to the Chado database (12). The Artemis gene builder has four tabs (Properties, Core, Controlled Vocabulary and Match) that provide curators with a comprehensive overview of each protein-coding gene model. The ‘Controlled Vocabulary’ section provides a way to add (or remove) CV terms assisted by an auto-completion field that ensure the compliance of the annotations with agreed standards. The ‘Match’ section displays the similarity found at the functional annotation step.

Moreover, to ensure a consistent and accurate standard of annotation using a CAS, we developed the Chado Controller (11), which contains three modules: (i) a module for managing user access rights to the features stored in the database (i.e. private and public project can be stored in a single database), (ii) a module for checking the manual annotation before edition in the database (annotation inspector) and (iii) a module for recording the history of the modifications (annotation history with the Chado ‘audit module’).

### Interoperability

**Figure 3.**
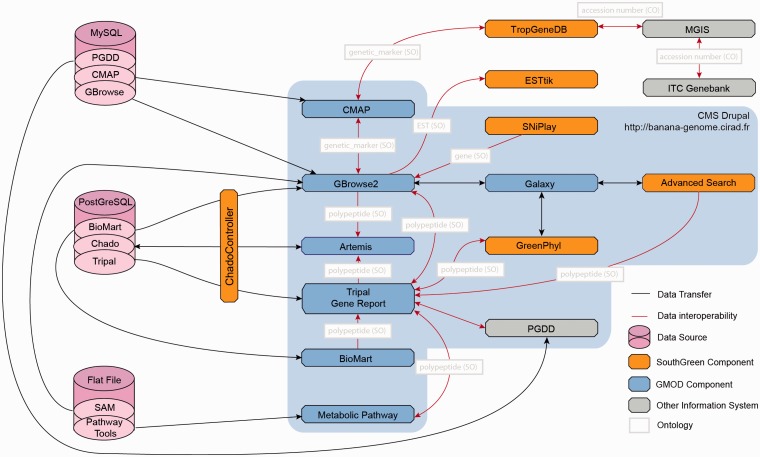
Architecture of the Banana Genome Hub and Interoperability between Biological Information Systems. Gene report can be displayed using Tripal, GBrowse or BioMart and edited with Artemis. Polypeptides can then be further analyzed with GreenPhylDB using for instance keywords and InterPro domains, with the Galaxy workflow manager by running personalized phylogenetic workflows and with Pathway Tools to study metabolic pathways through keywords or EC numbers. Finally, SNP stored in GBrowse can be investigated with SNiPlay. Genetic markers can be positioned on genetic maps using CMAP and investigated into the TropGeneDB, linked with the MGIS through ITC accession numbers. Germplasm material can then be requested to the ITC. Most of the systems were embedded using the Drupal CMS using HTML iframe.

As genomic data sets are distributed over multiple data sources (Table 3), a central function of the Banana Genome Hub is to link heterogeneous data and information systems (Figure 2). Providing different entry points to users and facilitating the data retrieval process in as minimum of steps were the driving principles of the work presented here. Conceptually, data consistency and quality is ensured by the CAS, whereas the interoperability with other systems is promoted by cross-links to their sites from Tripal and GBrowse (Figure 3) forged using unique identifiers and ontology. The Hub is organized around a protein-details page for each banana protein-coding gene. The page contains genomic data extracted from Chado and various links for associated data (e.g. gene families, metabolic pathways, etc.). The types of unique identifiers allowing the links are reported in the Figure 3.

**Table 3.** Relationships between data types and systems. ‘Yes’ displays the data content of the system. ‘Links’ correspond to URL integration of systems using unique identifiers

Data/Components	Tripal	ESTTIK	GBrowse	GreenPhylDB	MusaCyc	MGIS	PGDD	SNiPlay	CMAP
Gene report	Yes		Yes	Links	Links		Links	Links	
Genetic maps	Links		Links						Yes
Molecular markers			Yes			Links		Yes	Yes
Metabolic pathways	Links		Links	Links	Yes				
Phenotypes						Yes			Links
Proteins	Yes		Yes	Yes					
Proteins families	Links		Links	Yes					
Syntenic genes							Yes		
Transcripts		Yes	Yes

GBrowse 2, through its pop-up, proposes similar content and links based on unique identifiers that support persistent cross-links. GBrowse 2 was designed to facilitate data aggregation and can connect several data sources. We use this feature to aggregate Chado database, GBrowse MySQL database and BAM files (for RNA-Seq) using specific Perl database adaptor modules, respectively, Bio::DB::Das::Chado, Bio::DB::SeqFeature::Store and Bio::DB::Sam (Figure 1). This is particularly useful, as performance considerations dictated that we needed to apply different storage strategies depending on the type and volume of data. Although Chado remains the central database (for Artemis and Tripal), Chado sometimes suffers from poorer performance for data extraction. Therefore, GBrowse MySQL remains a valid complementary option for the storage of static data mapped to the banana genome such as *ab initio* gene prediction, ESTs matches and SNP markers. The Sequence Ontology gives a hierarchical structure to the data and Perl loaders guarantee their integrity. Finally, controlled vocabularies stored in the Chado Controller enforce curation practices, which promote data quality.

One of the main challenges we are facing is the need to propagate regular updates of the annotated genes or new genome release with associated functional data (e.g. cognate and similar UniProt entries). This need is confounded by the fact that each independent system has its own constraints and its own update protocol. For newly annotated genes in the CAS, modifications of the exon–intron structure of a banana gene often generate consequences. First, such modifications may change the result of protein domain search like InterProScan that are handled in different places (e.g. Chado, GBrowse MySQL, GreenPhylDB). Significant changes in the sequence, owing for instance to erroneous frame shifts or gene fusion, could result in an alternative protein clustering in GreenPhylDB clusters. In any case, the phylogenetic analyses may be affected as well as the ortholog predictions. Hence, introducing flexibility in gene annotation should be accompanied by the ability to update subsequent analyses.

As a step towards overcoming these challenges, we coupled our gene curation pipelines with an in-house version of the Galaxy workbench (44), to handle data resulting from our automatic pipelines. We implemented several analytical workflows to reproduce the analyses usually done at large scale via Perl scripts executed in command line. With a single click, GBrowse can send data to Galaxy for analysis. Conversely, we added to the Galaxy ‘get data’ menu, a tool that retrieves data through gbgff dumper packaged with GBrowse 2. This concept has been extended to GreenPhylDB and could be generalized in other systems supporting the Banana Genome Hub, in line with other initiatives (45).

Hereafter, we present several use cases that illustrate the use of the Banana Genome Hub to retrieve data, benefit from pre-computed analyses, edit incorrect predictions and update analyses. In its current state, the Banana Genome Hub functions well to facilitate phylogenetic studies and gene family analyses.

## Use Cases

### SPS Family

The first use case focuses on the SPS family. SPS are plant enzymes (E.C. 2.4.1.14) known to play a major role in sucrose biosynthesis (46–48). SPS genes are activated during osmotic stress and involved in sucrose accumulation during fruit development (49, 50). Several studies demonstrate a positive correlation between SPS activity and plant growth rate coupled with yield in important crops, though direct proof of a causal link is lacking (51). The evolution and function of the SPS gene family was studied in wheat and other grasses (52), highlighting several subfamilies, some of which probably arose after the monocotyledon dicotyledon divergence. An extended phylogenetic of this gene family described four unique SPS genes in *Arabidopsis*, five in rice and at least six in maize (53).

The banana SPS family was identified using search facilities of the *Musa* portal and GreenPhylDB using keywords related to the sucrose phosphate synthase function (Supplementary File 1 Figures S1 and S9). In GreenPhylDB, the SPS family appears as a cluster of 133 sequences (cluster GP016032 annotated as SPS subfamily) (Supplementary File 1 Figure S9), including four sequences of banana, distributed on three chromosomes (Supplementary Table S4). The SPS family belongs to a larger family of glycosyl transferase group 1 proteins, composed by 346 members (cluster GP000333), which includes the Sucrose synthase family. The four banana sequences have been manually annotated with the GNPAnnot CAS. The fifth exon of gene GSMUA_Achr4G16070_001 was extended (Supplementary File 1 Figure S3). This modification takes into account an AG/GC intron–exon junction not detected by the automatic gene prediction. The exons 11 and 12 of this gene have also been merged, as are the exons 11 and 12 of GSMUA_Achr4G05060_001 (Supplementary File 1 Figure S2), exons 1 and 2 and exons 13 and 14 of GSMUA_Achr6G17480_001, and exons 11 and 12 on GSMUA_Achr9G22510_001. These modifications are supported by additional evidence of gene structure like the ESTs. The ESTtik tools (Supplementary File 1 Figure S8) facilitated the retrieval of information about these ESTs. Through direct access to Artemis from GBrowse (Supplementary File 1 Figure S2), the genes were quickly corrected. Moreover, Gbrowse also links with a physical map and a genetic map of CMap for identifying genetic markers near such genes of interest (Supplementary File 1 Figure S4). All the SPS family members are characterized by the same InterPro signature (IPR006380—SPS domain) (Supplementary File 1 Figures S5 and S6). The SPS enzymes are characterized by EC 2.4.1.14, as shown in the sucrose biosynthesis pathway described within the Pathway Tools (Supplementary File 1 Figure S7).

No alignment has been made yet on the SPS subfamily in GreenPhylDB, but it is available by a phylogenic analysis linking to the cluster GP000333. This tree shows a distinct separation between the clade containing the SPS *Musa* genes and another containing the sucrose synthase family (Supplementary File 1 Figure S10). We can see that both monocotyledons and dicotyledons are represented in the smallest subtrees containing GSMUA_Achr6G17480_001 and GSMUA_Achr4G16070_001, suggesting that they arose from a duplication that occurred before the divergence between monocotyledons and dicotyledons. In contrast, the gene tree suggests that GSMUA_Achr9G22510_001 and GSMUA_Achr4G06050_001 are the result of a more recent duplication. The close proximity of GSMUA_Achr9G22510_001 and GSMUA_Achr4G06050_001 on the tree suggests that the duplication is specific to the *Musa* Group.

To evaluate the impact of manual curation of the phylogenic analysis, we performed a phylogenetic analysis with a Galaxy workflow developed by South Green that reproduced the GreenPhylDB pipeline but on a subset of checked polypeptide sequences: the four curated DH-Pahang SPS polypeptides and homologous sequences from the rice, sorghum and *Arabidopsis* genomes (selected from GreenPhylDB) (Supplementary File 1 Figures S11 to S14). The lower evolutionary distances between the genes GSMUA_Achr9G22510_001 and GSMUA_Achr4G06050_001 were confirmed by this analysis.

The two genes belong to the *Musa* α/β ancestral block number 4 (1). The analysis of local paralogous relationships with the PGDD tool shows that the two genes are present in the duplicated syntenic regions (Supplementary File 1 Figure S15), but not listed as duplicates. This might be due to local sequence rearrangements that can influence the results of global synteny analysis. The Ks value of 0.455 for the two genes is consistent with a divergence time of ∼50 MYA corresponding to the alpha/beta *Musa* WGD (Supplementary File 1 Figure S10).

### The cellulose synthase (CesA) and cellulose synthase-like (Csl) superfamily

The second use case relates to the CesA superfamily chosen because its members display a different distribution between eudicot and Poaceae species. We identified all members of the cellulose synthase (*CesA*) and cellulose synthase-like (*Csl*) gene superfamily analyzing their distribution in two eudicot reference species (*Arabidopsis* and grapevine), two grass reference species (the *Poaceae* family: rice, sorghum) and banana. The *CesA* and *Csl* genes encode members of the polysaccharide synthases/glycosyltransferases class of enzymes that are involved in cell wall biosynthesis. In plant primary cell walls, cellulose fibers are encased in a matrix of carbohydrates composed of hemi-cellulosic polysaccharides and of pectin. The composition in hemi-celluloses and pectin varies among flowering plants, leading to different types of primary cell wall structures, in particular, for monocot plants of the commelinid group (Aracales, Zingiberales, Poales) that includes *Musa* and the grasses, as opposed to eudicots and non-commelinid monocots (54, 55). The CESA proteins are involved in the synthesis of cellulose whereas the CSL family comprises several members that have been shown to be implicated in the synthesis of different hemi-cellulosic polysaccharides [reviewed in (56)]. The *Csl* genes are subdivided into nine families (*CslA* to *H* and *CslJ*), which show a different distribution between eudicots and *Poaceae* species (57, 58), and some correlation was found between this gene family structure and differences in cell wall composition between eudicots and the grasses (59, 60).

To identify *Musa* CESA and CSL sequences, we searched the *Musa* proteome using the protein combination tool of GreenPhylDB with InterPro domain IPR005150 corresponding to the CesA family (CESA, CSLB, CSLD-J) and IPR001173 corresponding to the Glycosyl transferase family 2 (CSLA, CSLC) (Supplementary File 2 Figure S1). The search using IPR005150 identified 34 sequences that clustered within the ‘Cellulose synthase’ family in GreenPhylDB. Predicted enzymes with the associated E.C. number E.C. 2.4.1.12 for CesA function that were retrieved from MusaCyc, corresponded to the same set of 34 sequences (Supplementary File 2 Figure S2). The search using IPR001173 in GreenPhylDB identified 32 sequences of which 26 clustered within the ‘Putative glycosyl transferase’ family that comprised CSLA and CSLC homologs from other species. Homologous sequences from the rice, sorghum, *Arabidopsis* and grapevine genomes were retrieved from GreenPhylDB and were assigned to the different subfamilies following published data (57, 58, 61) (Supplementary File 2 Figure S3). We manually curated the compiled 60 *Musa* genes using information from different *de novo* prediction programs, expression data and BLASTP data available on the banana GBrowse (Supplementary File 2 Figure S4). This resulted in a final set of 59 genes. All sequences were aligned using the program MAFFT (62) with default iterative refinement method. Maximum-likelihood phylogenetic analysis was performed using PhyML V.3.0 (63) with the Le-Gascuel (LG) evolution model, a gamma law with four categories and an estimated gamma distribution parameter, a *Nearest Neighbor Interchange* starting tree, and approximate likelihood ratio test for branches based on a Shimodaira–Hasegawa-like procedure.

**Figure 4.**
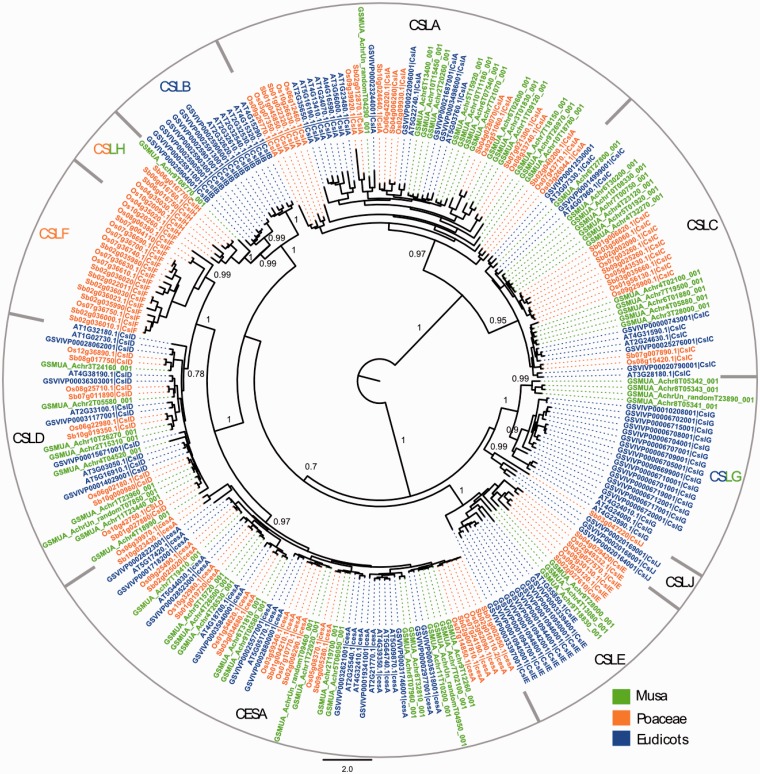
Maximum likelihood phylogenetic tree of the CESA and CSL families. Phylogenetic analysis was carried with full-length protein sequences from *Arabidopsis thaliana* (AT), *Vitis vinifera* (GSVIV), *Oryza sativa* (Os), *Sorghum bicolor* (Sb) and *Musa acuminata* (GSMUA). Branch support values correspond to approximate likelihood ratio test results. Scale represents number of amino acid substitutions per site. CSL subfamilies are indicated (CSLA to CSLH, CSLJ).

The results showed that the DH-Pahang genome comprises 16 *CesA* genes, which is relatively higher than *Arabidopsis* (10 genes), grape (11 genes), rice (10 genes) and sorghum (10 genes), but similar to poplar [18 genes, (61)] (Supplementary Table S2). We identified a total of 43 *Musa* CSL sequences in the *Musa* genome, distributed into six of the nine described subfamilies (Figure 4). The distribution of the 43 *Musa* CSL sequences is intermediate between a eudicot-like profile and a Poaceae-like profile. One of the two families (CslB and CslG), previously only observed in eudicots, is present in *Musa* (CslG, four genes). The CslH family, so far found only seen in Poaceae, has one member in *Musa*. Expression of the gene is inferred based on *Musa* RNA-Seq information displayed in the GBrowse, and the gene is therefore potentially functional. No *Musa* CSL sequence was found in the CslF and CslJ groups. CslJ sequences are present in some eudicots and monocots but are not always found in plant genomes (61). Genes of the CslF family are implicated in the synthesis of (1,3;1,4)-β-D-glucans (64), which in angiosperms are exclusively found in Poales cell walls. The absence of CslF genes in *Musa* supports an independent origin of these genes within the Poales. The organization of *Musa* Csl genes might be linked to the specific cell wall structure of non-grass commelinid monocots (55).

### 9-Cis-epoxycarotenoid Dioxygenase Family

For the final use case, we highlighted the 9-Cis-Epoxycarotenoid Dioxygenase Family (NCED), a small intron-less gene family belonging to a larger family of carotenoid cleavage dioxygenase (CCD) genes that can have introns, are implicated in abscisic acid biosynthesis, and are targeted to chloroplasts. NCED proteins have been shown to be involved in biotic and abiotic stress responses (65), fruit ripening (66, 67) and seed maturation (68) and therefore might present some interest for banana breeding.

A total of 13 DH-Pahang CCD gene members were identified from GreenPhylDB (search for IPR004294, carotenoid dioxygenase) and MusaCyc (search for EC 1.13.11.51) (Supplementary Table S3, Supplementary File 3 Figures S3 and S4). According to GreenPhylDB gene family curation, the Carotenoid dioxygenase family (GP000379 CCD) contains the NCED (GP069973 NCED) with 10 *Musa* NCED members. However, in MusaCyc, only seven NCED enzymes were found. To elucidate the numbers of NCED members in the DH-Pahang genome, we first checked whether the gene annotation was correct, using the GNPAnnot CAS. We used the quick search of Tripal to retrieve the CDS based on the uniquename (e.g. GSMUA_Achr5G02570_001; Supplementary File 3 Figure S5). A total of 10 poly-exonic NCED predicted genes were found (Supplementary File 3 Figures S2 and S6), inconsistent with the mono-exonic structure of the rice ortholog (OsNCED3/Os03g44380) (Supplementary File 3 Figure S7). Artemis was used to perform the manual curation and to restore the mono-exonic structure (Supplementary File 3 Figure S8). Two gene-fragments (GSMUA_Achr4G22870_001 and GSMUA_Achr4G22880_001) were merged, as they corresponded a single NCED gene. The monoexonic structure and the fusion of the two gene fragments were supported by public monocotyledon ESTs displayed in GBrowse 2.

**Figure 5.**
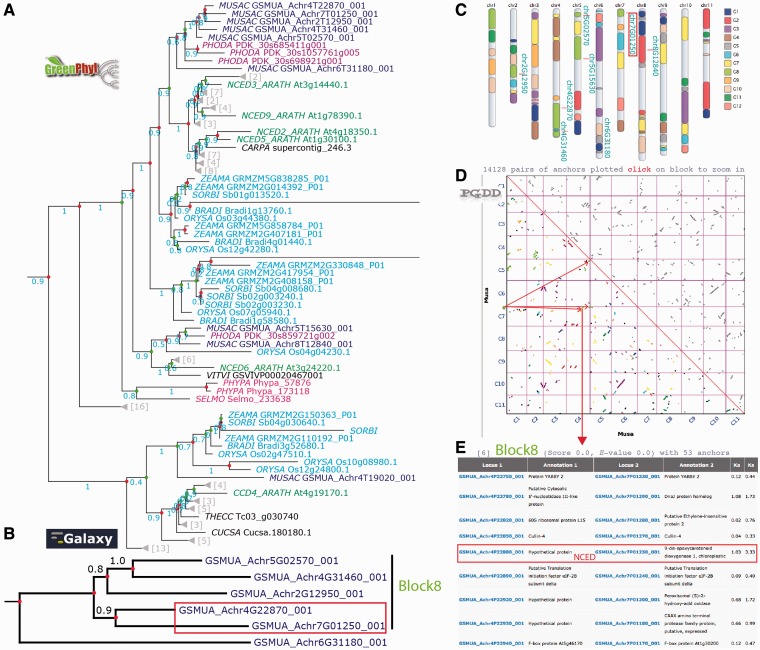
Analysis of the banana NCED gene duplication events. (**A**) The GreenPhylDB pre-computed polypeptide tree of the carotenoid dioxygenase family (GP000379 CCD) contains eight *Musa* 9-cis-epoxycarotenoid dioxygenase genes (GP069973 NCED Blue). CCDs are in cyan (Poaceae), purple (Arecaceae), green (Arabidopsis), magenta (moss). Green dots represent speciation events, whereas red dots represent duplication events. (**B**) The nucleotide tree of the six *Musa* NCED genes was performed after manual curation using an in-house Galaxy workflow. (**C**) Location of the NCED *Musa* genes on the Karyotype representation. *Musa* beta ancestral blocks are represented by the colored boxes within the chromosomes. (**D**) Clusters of *Musa* paralogous regions are represented on a PGDD dotplot. They are colored according to the beta ancestral blocks. (**E**) List of duplicated genes within the paralogous region containing GSMUA_Achr4G22870_001 and GSMUA_Achr7G01250_001 NCED genes.

On the GreenPhylDB CCD polypeptide tree (Figure 5A), the polypeptide GSMUA_Achr4G19020, was discarded from the NCED family, as its position in the tree corresponded to a sister group, including the *Arabidopsis* ARATH_CCD4 branch. Six *Musa* polypeptides clustered together in the vicinity of the group represented by [ARATH_NCED2, 5, 3 and 9] and two polypeptides (GSMUA_Achr8G12840 and GSMUA_Achr5G15630) were localized in the same group as ARATH_NCED6. The cluster of the six *Musa* NCED genes suggests duplications specific to *Musa* group.

To better understand the evolutionary history of the six *Musa* NCED genes, a nucleotide sequence phylogenetic analysis was done using an in-house Galaxy workflow (Supplementary File 3 Figure S9). The resulting gene tree (Figure 5B) identified two gene pairs as being the result of recent duplications in *Musa* (GSMUA_Achr5G02570_001, GSMUA_Achr4G31460_001) and (GSMUA_Achr4G22870_001, GSMUA_Achr7G01250_001). These four NCED genes are found on the *Musa* beta ancestral block 8 shown on the *Musa* paralogous region karyotype (in light green on Figure 5C). Four paralogous regions belonging to this ancestral block correspond to duplicated segments of *Musa* chromosomes 4 (two separate regions), 5 and 7 as shown on the dot plot in Figure 5D. Looking at the PGDD list of duplicated genes present on these regions, we found that GSMUA_Achr4G22870_001 and GSMUA_Achr7G01250_001 are present as duplicates (Figure 5E). The two other genes GSMUA_Achr5G02570_001 and GSMUA_Achr4G31460_001 are not listed, although they are present in the syntenic regions. This indicates that these two gene pairs resulted from *Musa* WGDs.

## Conclusion and Future Perspectives

The Banana Genome Hub aggregates for unified access various information systems and analytical tools that were not developed for the purpose of one specific crop. However, projects like the sequencing of the banana genome encouraged the synergistic integration of tools. In this publication, we presented a model of CAS that we found efficient, which we are promoting for other crop communities as a generic model applicable to other plant genomes. The three cases studies illustrate how genomic data for a given gene family can be easily compiled, curated and analyzed for relevant insights.

Given the development of NGS technologies, genome sequencing is becoming technically less challenging and less costly, resulting in many genomes being released every year. In addition, research communities will face the generation of a huge volume of new data including re-sequencing of related samples, transcriptomics (RNA-Seq), transcriptional regulation profiling (e.g. Chip-Seq), epigenetic studies, high-throughput genotyping and other related whole-genome functional studies. Thus, it is important to provide a tool that centralizes, provides easy access and allows exploiting huge amounts of data. Such a platform can be suitable to sustain re-sequencing efforts and to facilitate the updating of the genomic data in *Musa*. There will be some needs to develop additional visualization tools to highlight interspecific synteny and structural variations between multiple *Musa* genomes. The transcriptomic data will require efficient pipelines (e.g. Galaxy) for differential expression studies. These new data could also be useful for improving the reference genome annotation by supporting the refining of exon–intron junctions for instance. It will also help validating the gene predictions by confirming their expression in certain tissues and by characterizing their splice forms. These annotation improvements could then be provided in iterative updates of *Musa acuminata* genomic annotation. Owing to the importance of banana as a crop, the generation of SNP markers is an ongoing process, and we will need to focus our attention on the best way to represent these data. Finally, data integration remains a challenge, and the semantic integration of -omics data will be further investigated.

## Supplementary Material

Supplementary Data
