# Supplementary material for: The Banana Genome Hub
Source: Database (Oxford). 2013 May 23;2013:bat035. doi: 10.1093/database/bat035 (PMC3662865; doi:10.1093/database/bat035)
Supplement: Supplementary Data [file supp_2013_bat035_index.html]

Supplementary Data 

# The Banana Genome Hub

## Supplementary Data

files

**Files in this Data Supplement:**

- Supplementary Data - zip file
