# Supplementary material for: The Banana Genome Hub
Source: Database (Oxford). 2013 May 23;2013:bat035. doi: 10.1093/database/bat035 (PMC3662865; doi:10.1093/database/bat035)
Supplement: Supplementary Data [file supp_bat035_suppl_data.zip › Supplementary File 3.pdf]

Case Study of the Banana NCED Gene Family

Table of contents

1. Identification of the *Musa* NCED family 2
1.1. Search for a gene family 2
1.2. Search for a metabolic pathway 8

2. Annotation of the Musa NCED genes with the GNPAnnot CAS 9
2.1. Report NCED genes with the banana genome hub quick search 9
2.2. Show evidences for NCED genes with a genome browser 10
2.3. Similarity of banana NCED with *O. sativa* genome 11
2.4. Manual annotation of a NCED gene with a genome editor 12

3. Genomic comparative refinement 13
3.1. Phylogenetic analyses 13
3.2. Musa paralogous region analysis 15
3.2.1. Matching ancestral blocks with an advanced search 15
3.2.1. Using ancestral blocks on a dotplot 16
3.2.2. Checking back NCED gene contexts on the genome browser 16
3.2.3. SNP analysis 18

4. Query builder to export results 19

Figure 1: Search for NCED gene family (GreenPhylDB). 2
Figure 2: Structure of the carotenoid dioxygenase gene family (GP000379, CCD) and family composition of the 9-cis-epoxycarotenoid dioxygenase gene family (GP069973, NCED) (GreenPhylDB). 4
Figure 3: Banana carotenoid dioxygenase gene family (GP000379, CCD) and phylogenomic analysis of the 9-cis-epoxycarotenoid dioxygenase (NCED) family (GreenPhylDB). 6
Figure 4: Search enzymes in metabolic pathways (MusaCyc). 8
Figure 5: Banana Genome Hub Home and quick search for a gene (Tripal). 9
Figure 6: The genome browser displays evidences for *Musa* gene structures (GBrowse). 10
Figure 7: Rice genome browser displays evidences for a co-ortholog of a banana NCED gene (GBrowse). 11
Figure 8: Editing a NCED *Musa* gene (Artemis). 12
Figure 9: Dynamic phylogenetic analyses with Galaxy worflow manager. 13
Figure 10: Locate genes on ancestral blocks of *Musa* paralogous regions (Advanced Search). 15
Figure 11 Dot plot *Musa* beta ancestral blocks (PGDD). 16
Figure 12: Gene context of two *Musa* paralogous regions containing a NCED gene (GBrowse). 18
Figure 13: SNP analysis (SNiPlay). 18
Figure 14 Build a query (Biomart). 19

In this use case, we studied the evolutionary history of the 9-Cis-Epoxycarotenoid Dioxygenase (NCED) gene family of the *Musa acuminata* double-haploid Pahang proteome using several information systems available from the Banana Genome Hub. *M. acuminata* is a giant herb of the monocotyledon class.

To start with the Banana Genome Hub, go to http://banana-genome.cirad.fr/

1. Identification of the *Musa* NCED family

To identify the members of a gene family with the Banana Genome Hub several approaches are possible. In this example, we proposed a phylogenomic and a metabolic approach.

1.1. Search for a gene family

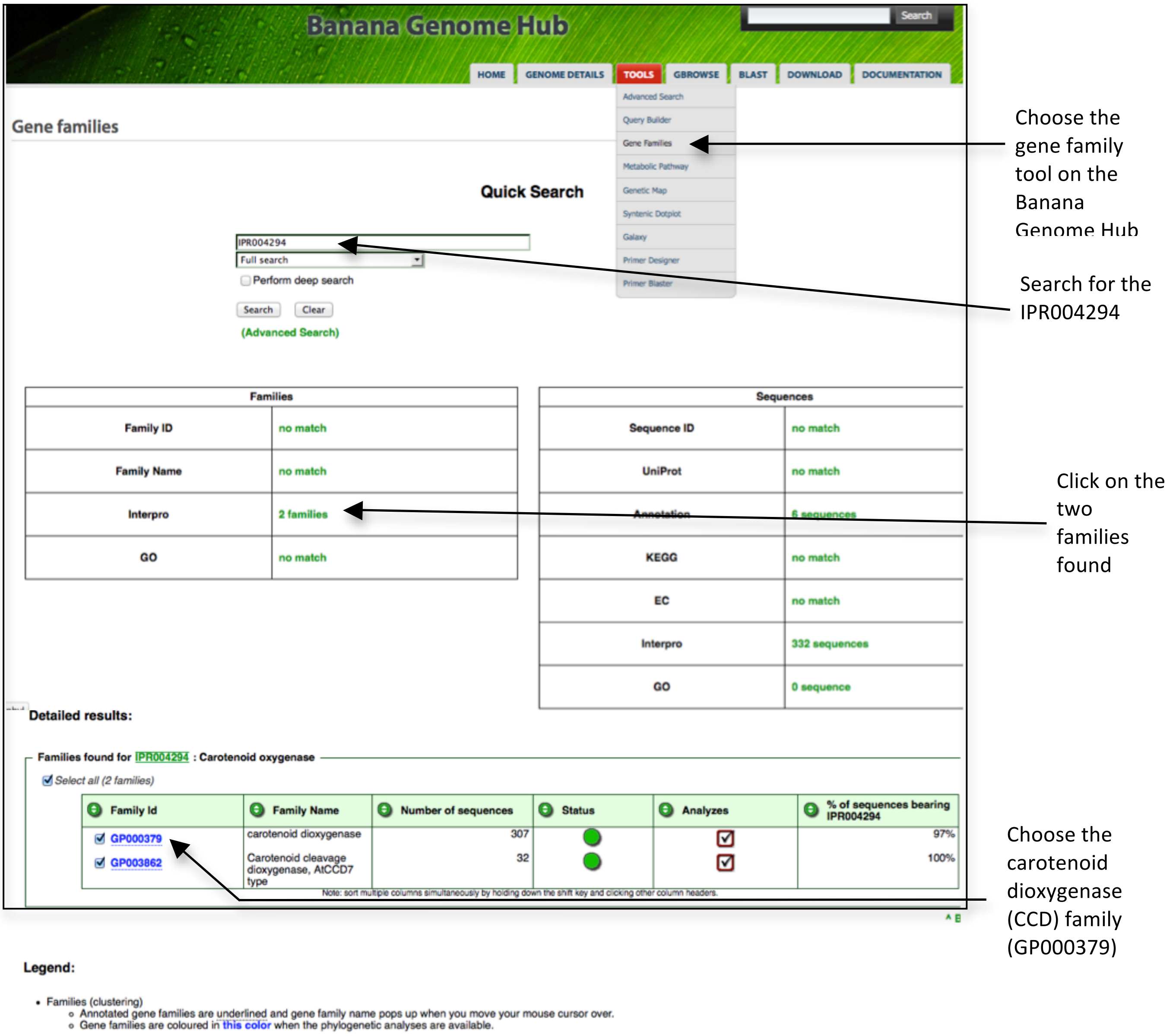

Figure 1: Search for NCED gene family (GreenPhylDB).

Method

The search for the Carotenoid oxygenase family was done with GreenPhylDB (available through the Gene Families link of the TOOLS tab of the Banana Genome Hub Web SiteFigure 1).
http://banana-genome.cirad.fr/greenphyl
Here, we searched for the family with the Interpro family identifier IPR004294 (Carotenoid oxygenase). Then click to the GP000379 Family id to see its detail report (Figure 2).

Results

Two families were found (at the less stringent clustering level): carotenoid dioxigenase (GP000379, 307 sequences) and carotenoid cleavage dioxygenase (GP003862, 32 sequences).
The Gene family GP000379 has been curated (Family id is underlined) and annotated as ‘carotenoid dioxygenase’ (CD). Curation status gives a High/normal confidence level to this family. The Phylogenetic analyses were done (family id is blue). For more, see Figure 2 and Figure 3.

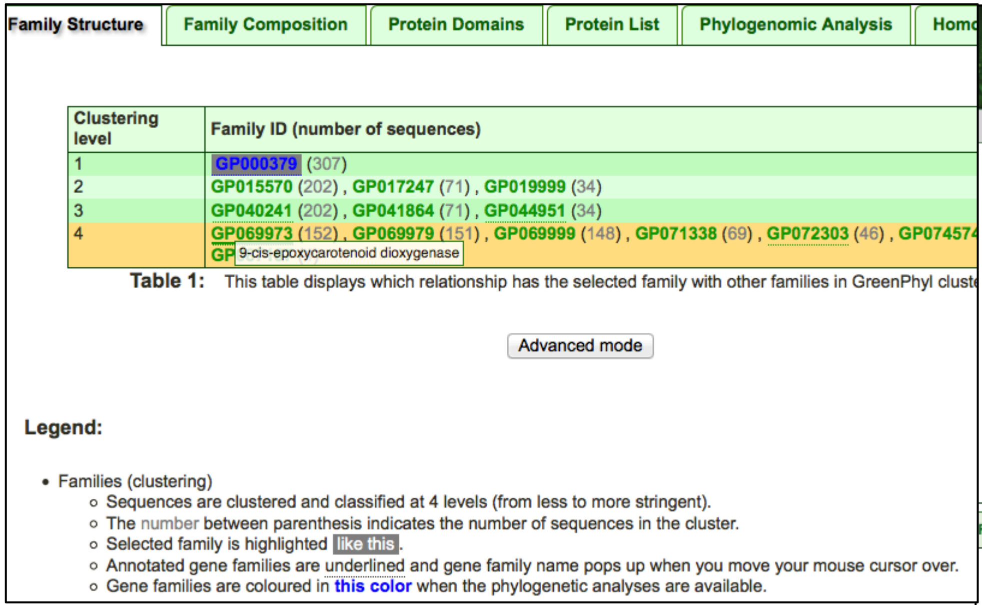

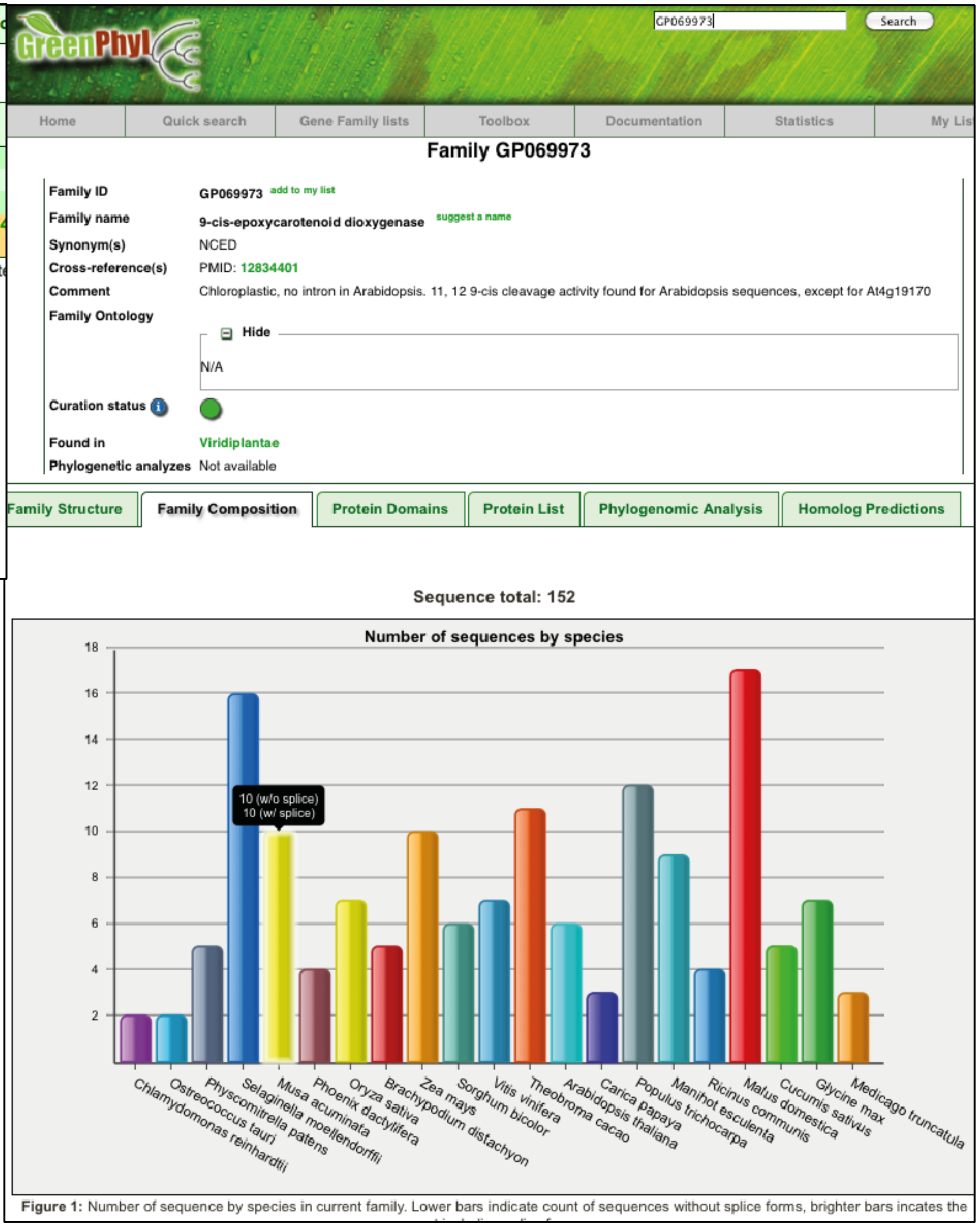

Figure 2: Structure of the carotenoid dioxygenase gene family (GP000379, CCD) and family composition of the 9-cis-epoxycarotenoid dioxygenase gene family (GP069973, NCED) (GreenPhylDB).

Method

You can go to the family report from **Figure 1** or Quick search for the family ID GP000379, click on the Family ID until arrive on
http://www.greenphyl.org/cgi-bin/family.cgi?p=id&family_id=379
http://www.greenphyl.org/cgi-bin/family.cgi?p=id&family_id=69973

Results

The Gene family GP069973 (subfamily at clustering level 4 in Family Structure tab of GP000379) has been annotated as 9-cis-epoxycarotenoid dioxygenase (152 sequences) but the phylogenetic analysis is not available (GP000379 family id is colored in green and not in blue). Experts decided to run the phylogenetic analyses at the Clustering level 1 for this family.

In the sequence number distribution among plant species complete proteomes, the *M. acuminata* one contains 10 NCED (see GP069973 Family Composition tab; Table 1).

Table 1: 10 *Musa* NCED belonging to the GP069973 family (GreenPhylDB)

<u>Method</u>

This table was exported from
http://www.greenphyl.org/cgi-bin/family.cgi?p=id&family_id=69973
by clicking on family composition, then *Musa* bar, finally Excel link (Figure 2).

Sequence ID	InterPro	Annotation
GSMUA_Achr4T22870_001	IPR004294	9-cis-epoxycarotenoid dioxygenase 1, chloroplastic
GSMUA_Achr5T02570_001	IPR004294	9-cis-epoxycarotenoid dioxygenase 1, chloroplastic
GSMUA_Achr2T12950_001	IPR004294	9-cis-epoxycarotenoid dioxygenase 1, chloroplastic
GSMUA_Achr4T19020_001	IPR004294	Putative Probable carotenoid cleavage dioxygenase 4, chloroplastic
GSMUA_Achr7T01250_001	IPR004294	9-cis-epoxycarotenoid dioxygenase 1, chloroplastic
GSMUA_Achr8T12840_001	IPR004294	9-cis-epoxycarotenoid dioxygenase 1, chloroplastic
GSMUA_Achr4T22880_001		Hypothetical protein
GSMUA_Achr5T15630_001	IPR004294	9-cis-epoxycarotenoid dioxygenase 1, chloroplastic
GSMUA_Achr6T31180_001	IPR004294	NCED3
GSMUA_Achr4T31460_001	IPR004294	9-cis-epoxycarotenoid dioxygenase 1, chloroplastic

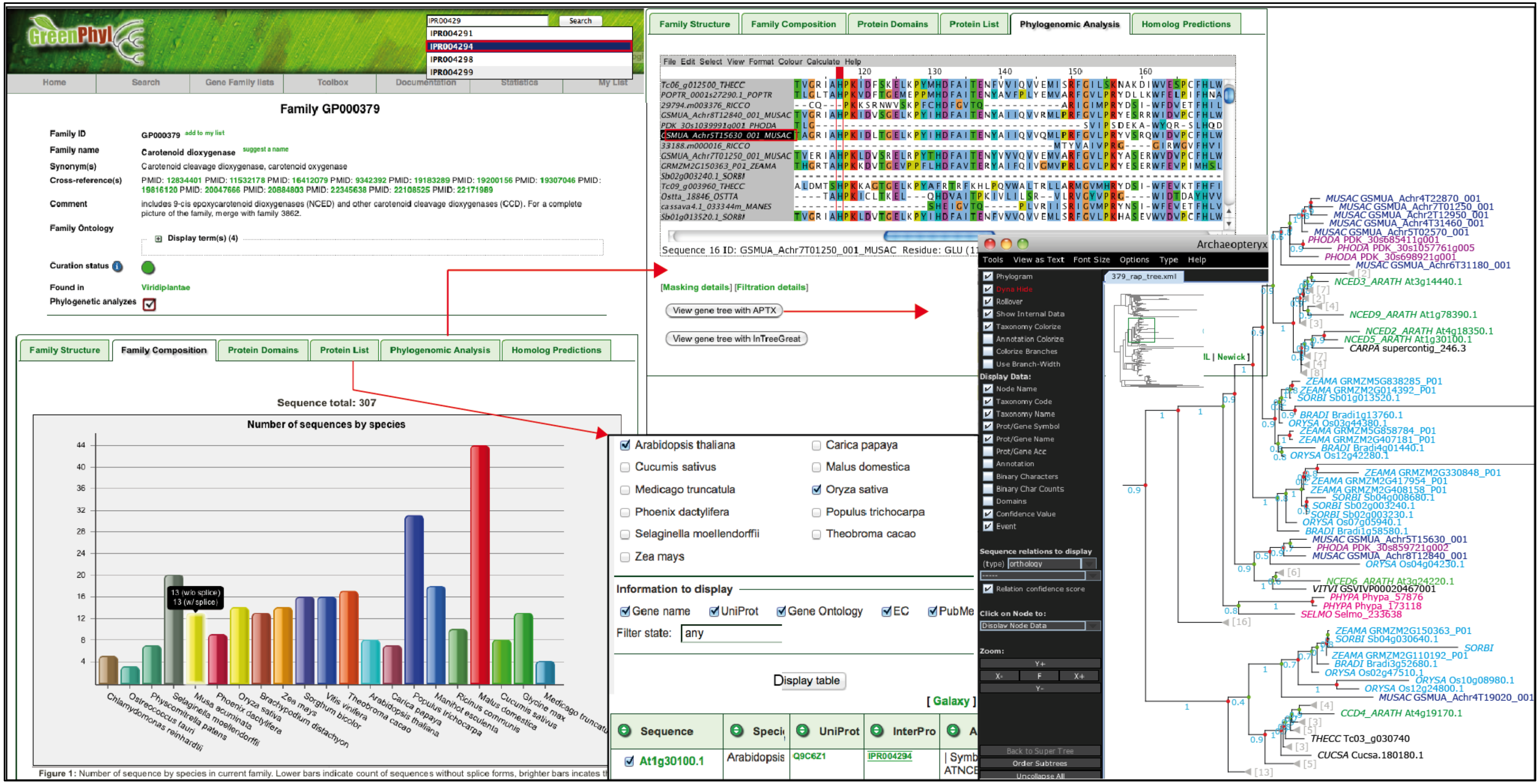

Figure 3: Banana carotenoid dioxygenase gene family (GP000379, CCD) and phylogenomic analysis of the 9-cis-epoxycarotenoid dioxygenase (NCED) family (GreenPhylDB).

Method

You can go to the family report from **Figure 1** or Quick search for the family ID GP000379, click on the Family ID until reaching http://www.greenphyl.org/cgi-bin/family.cgi?p=id&family_id=379

In the PhyML subtree, CCDs are in blue (Musaceae), cyan (Poaceae), purple (Arecaceae), green (Arabidopsis), magenta (moss). Green dots represent speciation events whereas red dots are duplication events.

Data uploading to Galaxy workflow manager (Figure 9) can be made from the Protein List tab in GreenPhylDB, after the selection of species.

Results

The DH-Pahang *Musa acuminata* proteome contains 13 CCD according to GreenPhylDB clustering (see GP000379 Family Composition tab). Histidine residues coordinating the catalytic iron, have been aligned and conserved in the pre-computed filtered multiple protein sequence alignment from MAFFT (see GP069973 Phylogenomic Analysis tab). In the PhyML pre-computed tree, viewed with the Archaeoptery Applet Java, available from the Phylogenomic Analysis tab, *Musa* polypeptides are separated into three groups containing dicotyledon CCD genes such as those of Arabidopsis (ARATH species code) suggesting duplications before the monocotyledons and dicotyledons divergence and whole genome duplications (WGD) specific to Zingiberales. We carried out the functional annotation to distinguish NCED from CCD enzymes using the *Arabidopisis thaliana* annotation provided by SwissProt, the reviewed section of the UniProt KnowledgeBase. The CCD family contains eight *Musa* NCED genes according (GSMUA_Achr4T22870_001, GSMUA_Achr8T12840_001, GSMUA_Achr5T15630_001, GSMUA_Achr5T02570_001, GSMUA_Achr6T31180_001, GSMUA_Achr7T01250_001, GSMUA_Achr2T12950_001, GSMUA_Achr4T31460_001) to Arabidopsis NCED annotation (NCED2, 3, 5, 6 and 9). GSMUA_Achr4T22880_001 was merged with GSMUA_Achr4T22870_001 during the manual curation process (see section 2). GSMUA_Achr4G19020 classified in the NCED GreenPhylDB family but seem to be in fact a CCD gene as its *Arabidopsis* ortholog is CCD4.

1.2. Search for a metabolic pathway

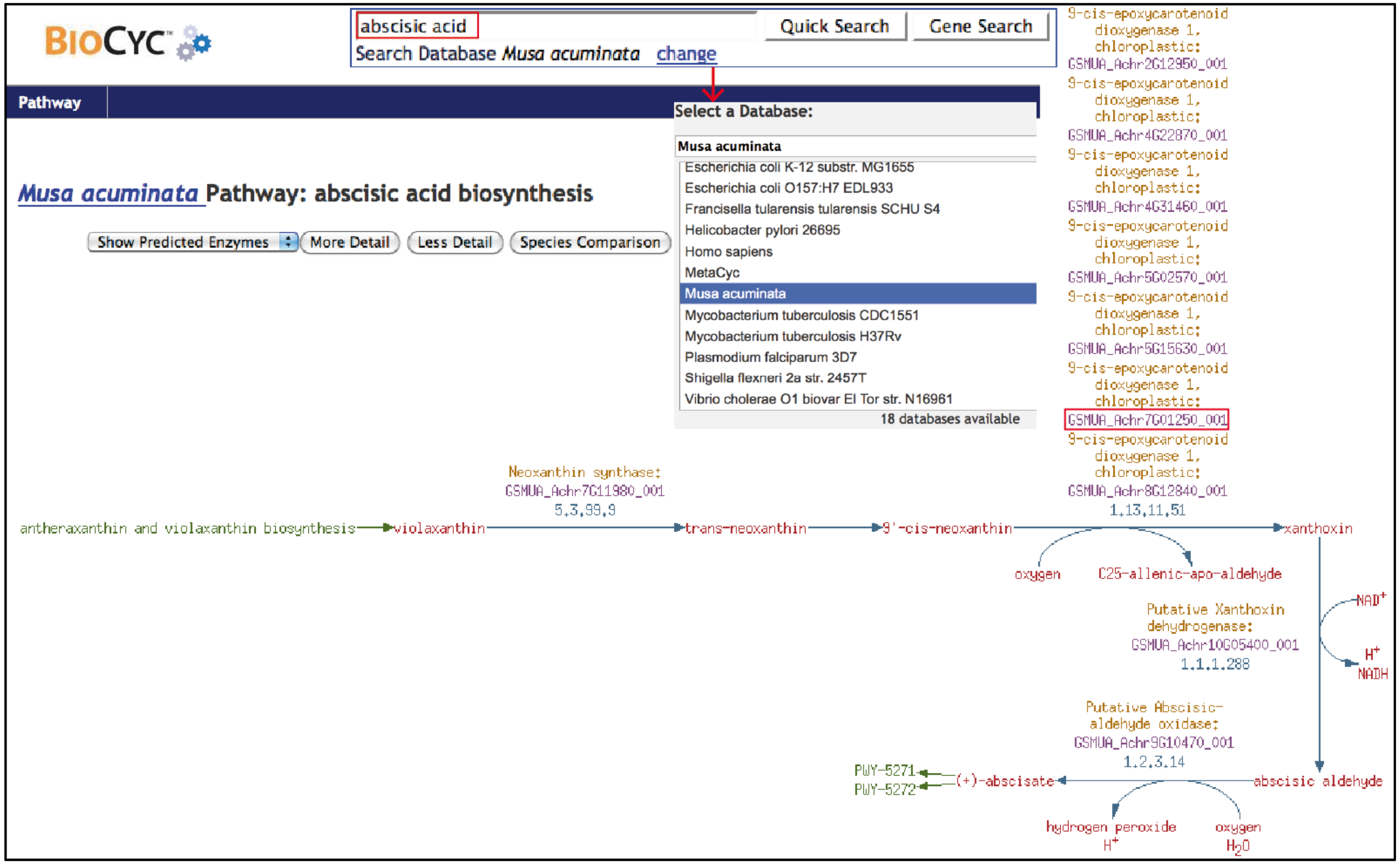

Figure 4: Search enzymes in metabolic pathways (MusaCyc).

Method

Pathway Tools allowed to create the banana Pathway/Genome Databases (PGDB), MusaCyc (available through the Metabolic Pathway link of the TOOLS tab of the Banana Genome Hub).

http://banana-genome.cirad.fr/musacyc

Results

NCED sequences were also searched with EC1.13.11.51 (9-cis-epoxycarotenoid dioxygenase in the abscisic acid biosynthesis) in MusaCyc (Pathway Tools). Seven *Musa* NCED were found and GSMUA_Achr6G31180_001 was missing compared to the gene family search described above.

2. Annotation of the Musa NCED genes with the GNPAnnot CAS

2.1. Report NCED genes with the banana genome hub quick search

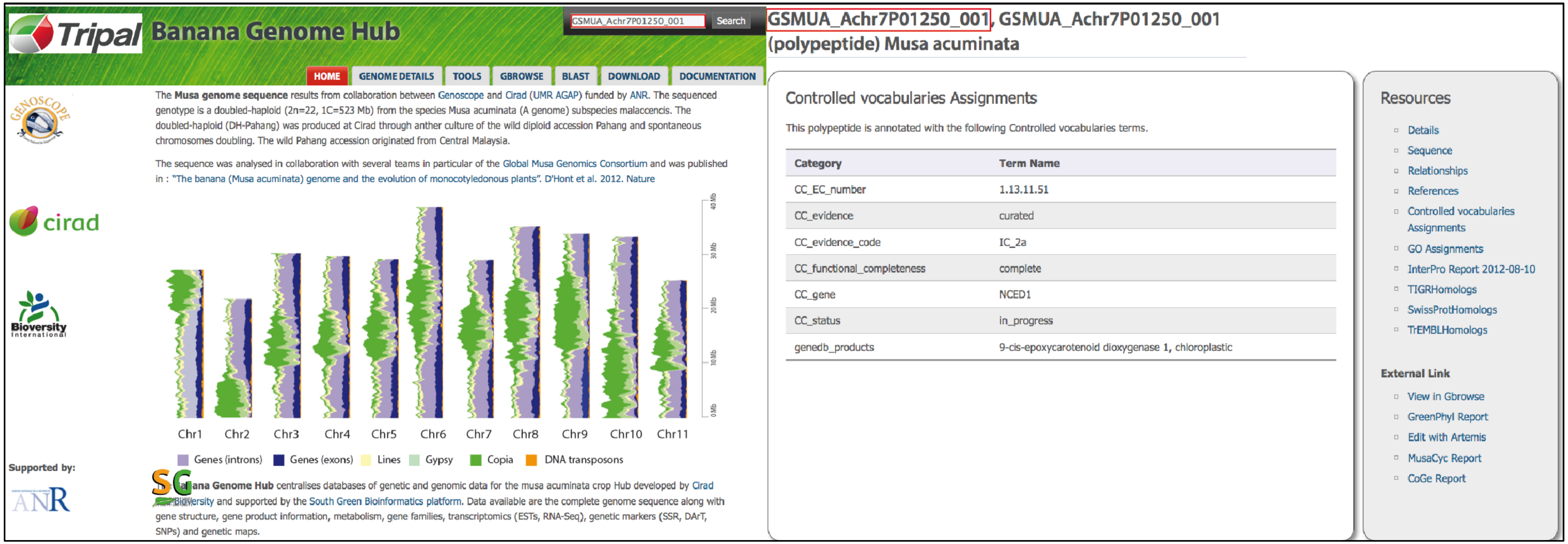

Figure 5: Banana Genome Hub Home and quick search for a gene (Tripal).

<u>Method</u>

Locus tags of a *Musa* NCED gene, such as GSMUA_Achr5G02570_001, were searched through the Tripal component of the GNPAnnot Community Annotation System (CAS) in order to access to its reports.

http://banana-genome.cirad.fr/GSMUA_Achr7P01250_001

Tripal is the Web front end of the CAS allowing gene report but also the Banana Genome Hub allowing integration of several tools (*e.g.* feature search, sequence analyses, bio-information systems, data download, external links).

2.2. Show evidences for NCED genes with a genome browser

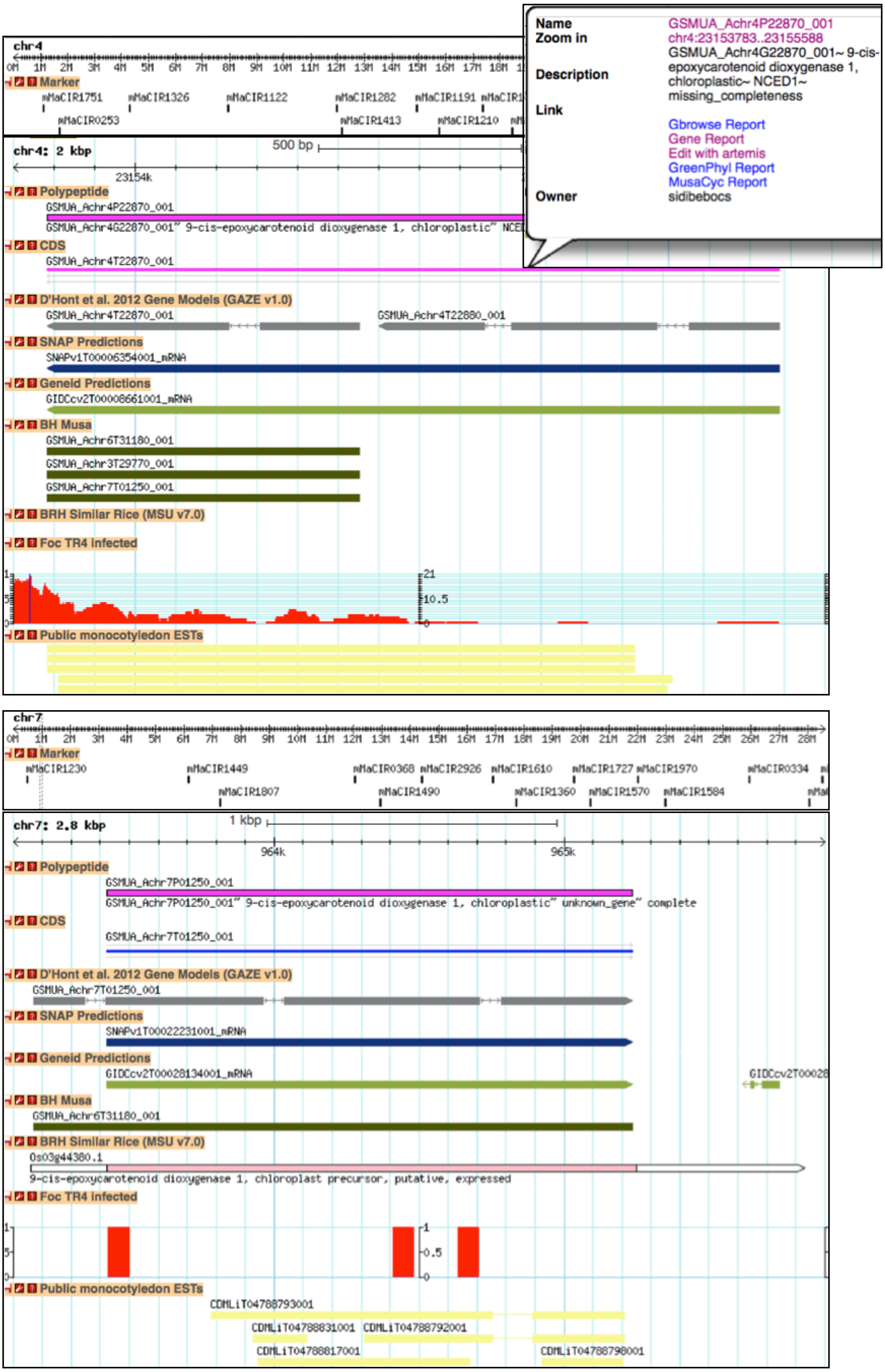

Figure 6: The genome browser displays evidences for *Musa* gene structures (GBrowse).

<u>Method</u>

The banana genome browser is available through the GBrowse tab of the Banana Genome Hub.
http://banana-genome.cirad.fr/cgi-bin/gbrowse/musa_acuminata/?name=chr7:963424..965235

The GMOD genome browser, GBrowse 2, displays evidences supporting a genomic feature and its genomic context. It can be queried by locus_tag or by genomic location.

<u>Results</u>

The automatic GAZE prediction made by the Genoscope of GSMUA_Achr7G01250_001 was poly-exonic. This structure was not supported by any of the *ab initio* gene finders (*i.e.* Snap, Geneid) or extrinsic evidences (*e.g.* monocotyledon EST). Moreover two NCED gene fragments were predicted (GSMUA_Achr4G22870_001 and GSMUA_Achr4G22880_001) instead of one (extended GSMUA_Achr4G22870_001 after curation).

2.3. Similarity of banana NCED with *O. sativa* genome

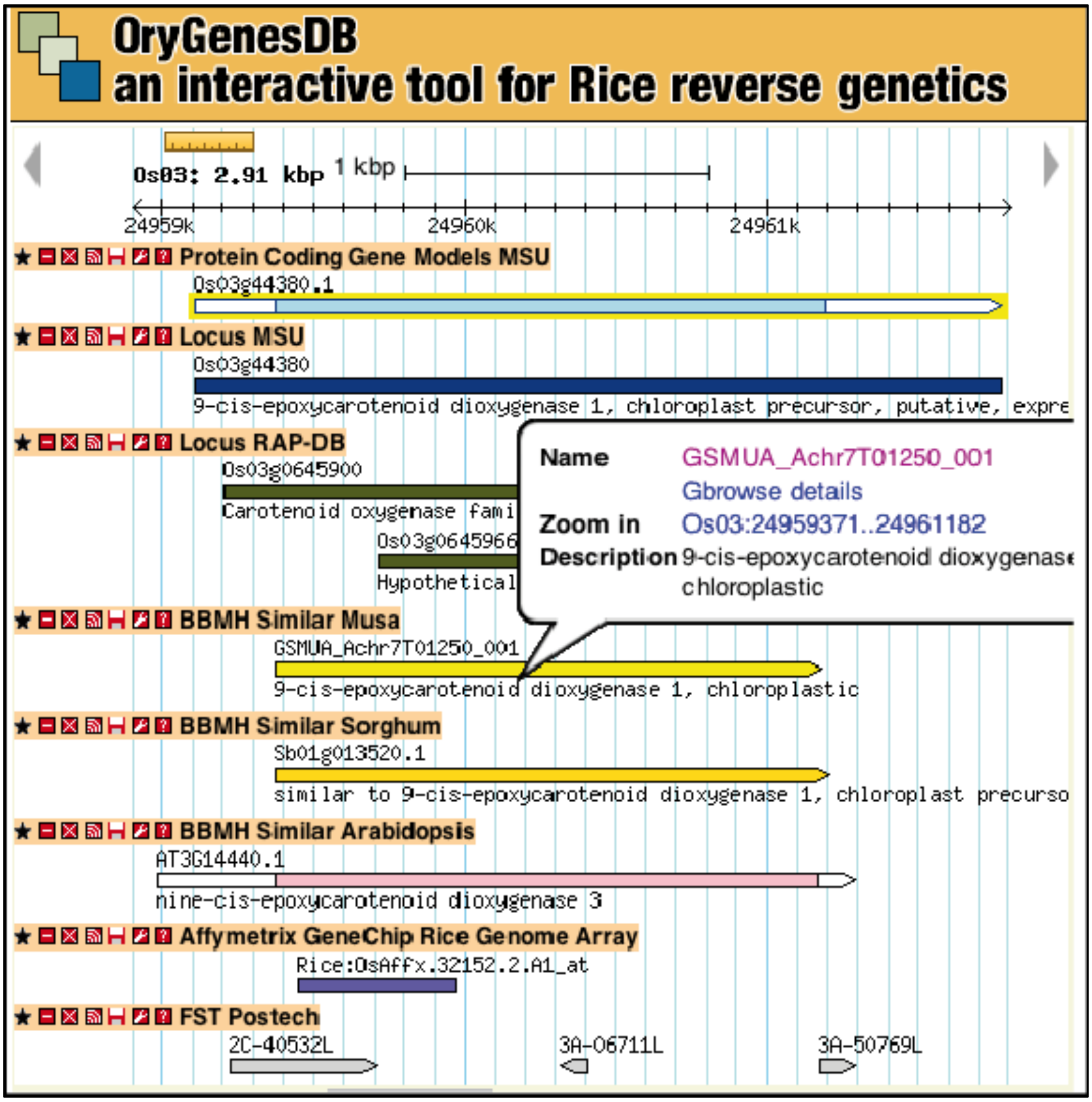

Figure 7: Rice genome browser displays evidences for a co-ortholog of a banana NCED gene (GBrowse).

<u>Method</u>

Rice genome is the model genome for the monocotyledon. One can browse in both directions between the DH-Pahang GBrowse and the OryGenesDB GBrowse. They are connected *via* URL that used the locus tag identifier as common identifier and links can be found in the popup balloons of GBrowse track Best Hits. So the OrygenesDB can also been entry point of the Banana Hub.

http://orygenesdb.cirad.fr/cgi-bin/gbrowse/odb_japonica/?name=Os03g44380.1

<u>Results</u>

The poly-exonic structure predicted by GAZE for the gene GSMUA_Achr7G01250_001 is not supported by its rice co-ortholog NCED3 Os03g44380 (MSU) found as intronless in OryGenesDB. We noted that Os03g44380 is present on Affimetrix GeneChip Rice Genome Array and a two T-DNA flanking sequence tag (FST) mutants exist that can help to better characterize the gene expression and polypeptide function if necessary.

2.4. Manual annotation of a NCED gene with a genome editor

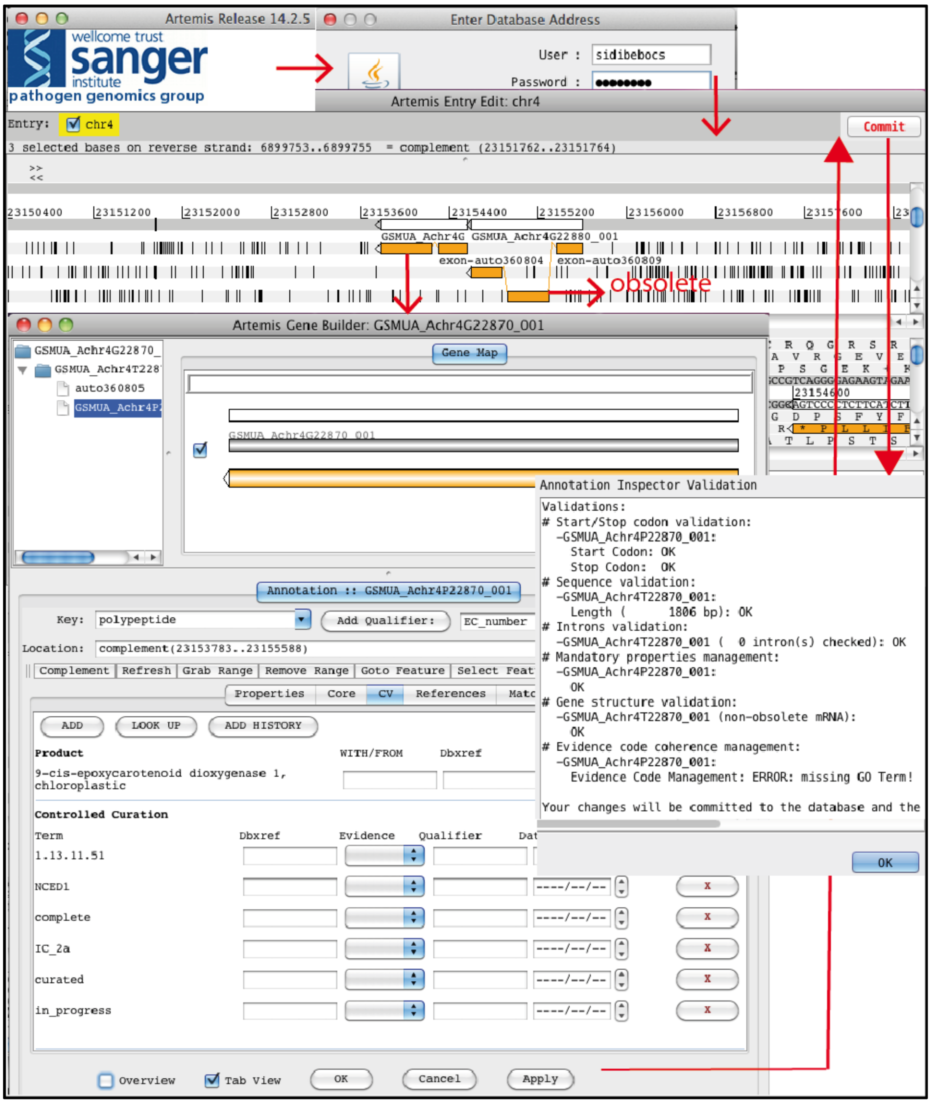

Figure 8: Editing a NCED *Musa* gene (Artemis).

Method

Artemis annotation tool can be launched from Tripal gene page or via any GBrowse balloon.
http://banana-genome.cirad.fr/cgi-bin/artemis.pl?species=musa;name=chr4:23128782..23180588
If the file.jnlp does not download when clicking on this link then copy paste it in a Firefox browser for instance. There is two ways to use the annotation tool: if you have an account, you can participate to the community annotation system by modifying features stored into the Chado Database. If you do not have an account, you can ask an account (see http://banana-genome.cirad.fr/content/annotation-account-request on the http://banana-genome.cirad.fr/gene_annotation accessible in the TOOLS tab of the Banana Genome Hub Web site) or connect to Artemis with a read-only account: banana_read_only without password and modifications can be saved on your local computer.

The inspector module of the Chado Controller checked manual annotation consistency. The advantage of working on a Chado database and not on flat files is that the annotations are managed via a Web information systems allowing sharing annotation, annotation backup and up to date homogeneous annotation. To have more information about how to annotate a gene structure, see the Protocols for gene annotation and the Chado Controller publication.

http://banana-genome.cirad.fr/documentation
http://www.gnpannot.org/biblio

Results

Both annotations, structural and functional, of GSMUA_Achr4G22870_001 were done with the Artemis genome editor. The coding exons were deleted except one which has been extended in order to restore the

mono-exonic structure. Most of the manual functional annotation consists in controlled vocabulary (*e.g.* qualifier gene symbol is NCED, product is 9-cis-epoxycarotenoid dioxygenase and EC_number is 1.13.11.51).

3. Genomic comparative refinement

3.1. Phylogenetic analyses

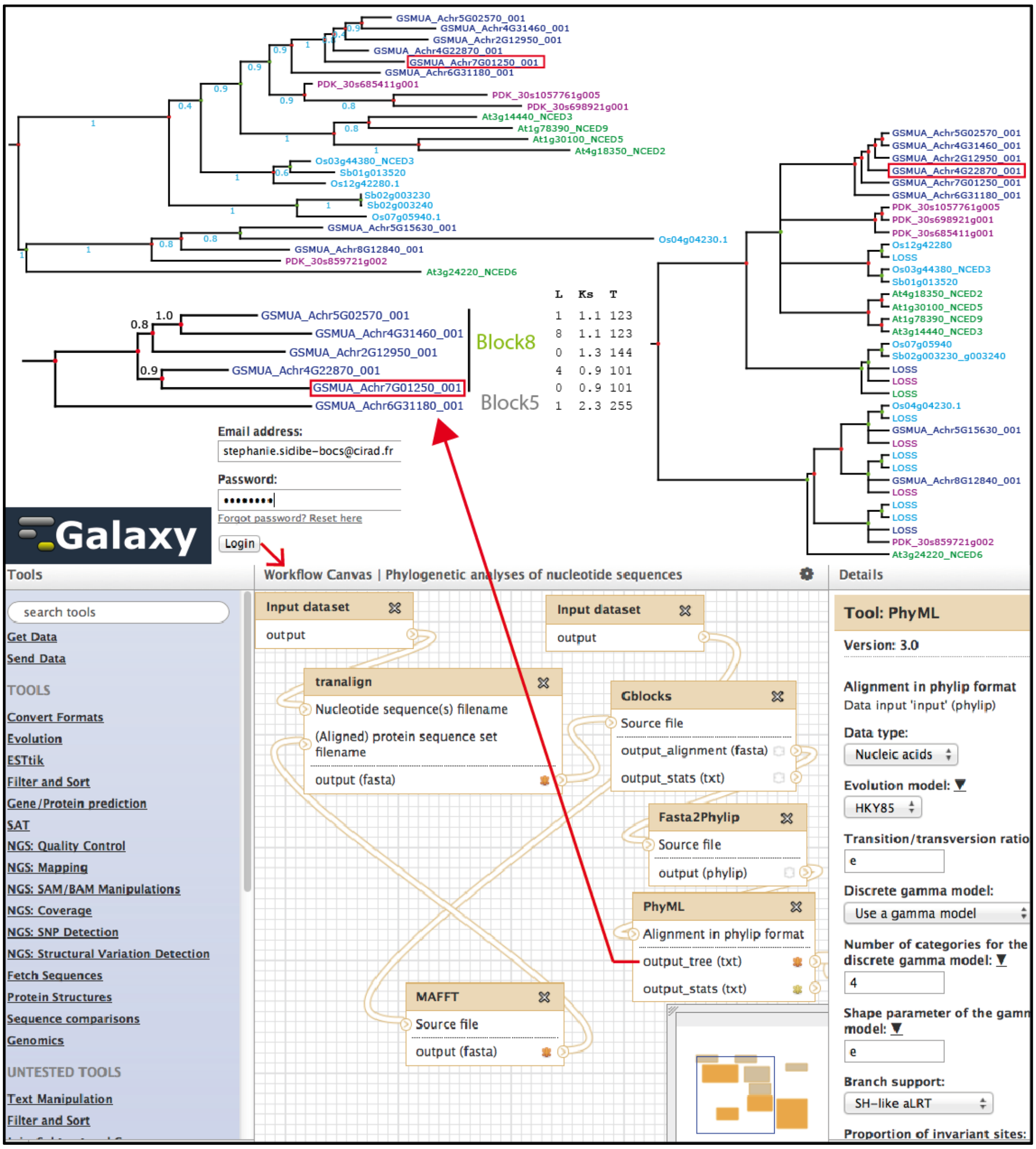

Figure 9: Dynamic phylogenetic analyses with Galaxy worflow manager.

<u>Method</u>

Galaxy workflow manager allows running easily complex sequence analyses.

http://gohelle.cirad.fr/galaxy/

Data uploading to Galaxy can be made from the Advanced Search tool of the Banana Genome Hub or from the Protein List tab in GreenPhylDB, after choosing species (Figure 3). You can also upload our own data through the uploading tool of Galaxy. Once the data uploaded, they will appear in green on the right of the screen. They will be available with a number that can be used by a Galaxy tool. Even if you are not logged in, you can use the Galaxy tools accessible in the left panel of the screen.

For users who have an account, the workflows are available on the Workflows tab. For those who do not have one, the workflows will not be available, but the tools are still accessible. You can apply for a account in the computer center section of the SouthGreen Web site.

http://gohelle.cirad.fr/cluster/compte_galaxy.php

The workflows can be modified in the edit section of the workflow menu accessible by clicking on the arrow next to the workflow's name. The different steps are visible in the central part of the screen. The parameters can be modified for each step in the Details panel.

In order to refine automatic phylogenomic analysis, GreenPhyl Phylogenomic analysis workflow (MAFFT with default iterative refinement method, and PhyML using LG+Γ model and providing aLRT+SH-like branch supports) was performed on a subset of NCED genes (banana, rice, sorghum, date palm, *Arabidopsis*). For the correct execution of the last step, tree reconciliation, of both workflows, the header of the plant NCED polypeptide fasta sequences of the input file should be correctly formatted with the five letter species code (*e.g.* GSMUA_Achr5G15630_001_MUSAC) and the species tree has to be in PhyloXML format. The resulting Newick and PhyloXML gene trees can be viewed using Archeopterix tree viewer. The gene tree topology was further analyzed in the light of the DH-Pahang *Musa* Ks and expression data. L reports the number of mapped reads per kilobase of CDS per million mapped reads (RPKM).

Results

The GreenPhylDB phylogenetic analyses were further completed using the Galaxy GreenPhyl-like workflow with the height corrected DH-Pahang polypeptide sequences and homologous sequences from the rice, sorghum, date palm and *Arabidopsis* genomes (selected from GreenPhylDB). Based on the reconciled gene trees (RAPGreen), we tried to reconstruct the evolutionary history of the DH-Pahang NCED family. We expected to observe *Musa* genes as an outlier clade of *Poaceae* genes with a speciation node between monocotyledon and dicotyledon. Among the eigth *Musa* NCED, Six *genes* clustered together in the vicinity of the group represented by [AtNCED2, 5, 3 and 9] and two genes (GSMUA_Achr8G12840 and GSMUA_Achr5G15630) were localized in the same group as AtNCED6.

In both GreenPhylDB (Figure 3) and Galaxy (this figure), two duplications prior to the divergence of monocots and dicots were visible: one with two *Musa* NCED members (the most ancestral), one with only *Poaceae* members and with the six *Musa* members.

Differences were also observed between the GreenPhylDB and Galaxy results. We first looked at the multiple alignments and the cleaned alignment obtained with Galaxy was longer than in the GreenPhylDB (499 aa vs 283 aa). This can be explained by the fact that it was based only on five species (banana, date palm, rice, sorghum and *Arabidopsis*), in contrast compared to the 22 taxa included in GreenPhylDB. It is interesting to note that the four histidine residues required for the catalytic iron activity, were maintained in both alignments. Differences in tree topology (*e.g.* position of date palm sequences and Os04g04230.1) could also be due to deficiencies in the sequence, assembly or annotation of other sequenced plant genomes (e.g. sequencing quality, annotation quality, gene fragment, pseudogenes).

At the intra-specific phylogeny level, some topologies were also different. The gene GSMUA_Achr5G02570_001-GSMUA_Achr4G31460_001 pair was predicted in the Galaxy gene tree but not in GreenPhylDB. Finally, a nucleotide sequence phylogenetic analysis was done (in-house Galaxy GreenPhyl Phylogenomic analysis workflow (nucleic)) only for the cluster of the six *Musa* NCED genes where the topology differed between both analyses. The resulting gene tree confirmed the GSMUA_Achr5G02570_001 and GSMUA_Achr4G31460_001 gene pair and created a new pair (GSMUA_Achr4G22870_001 and GSMUA_Achr7G01250_001).

3.2. Musa paralogous region analysis

3.2.1. Matching ancestral blocks with an advanced search

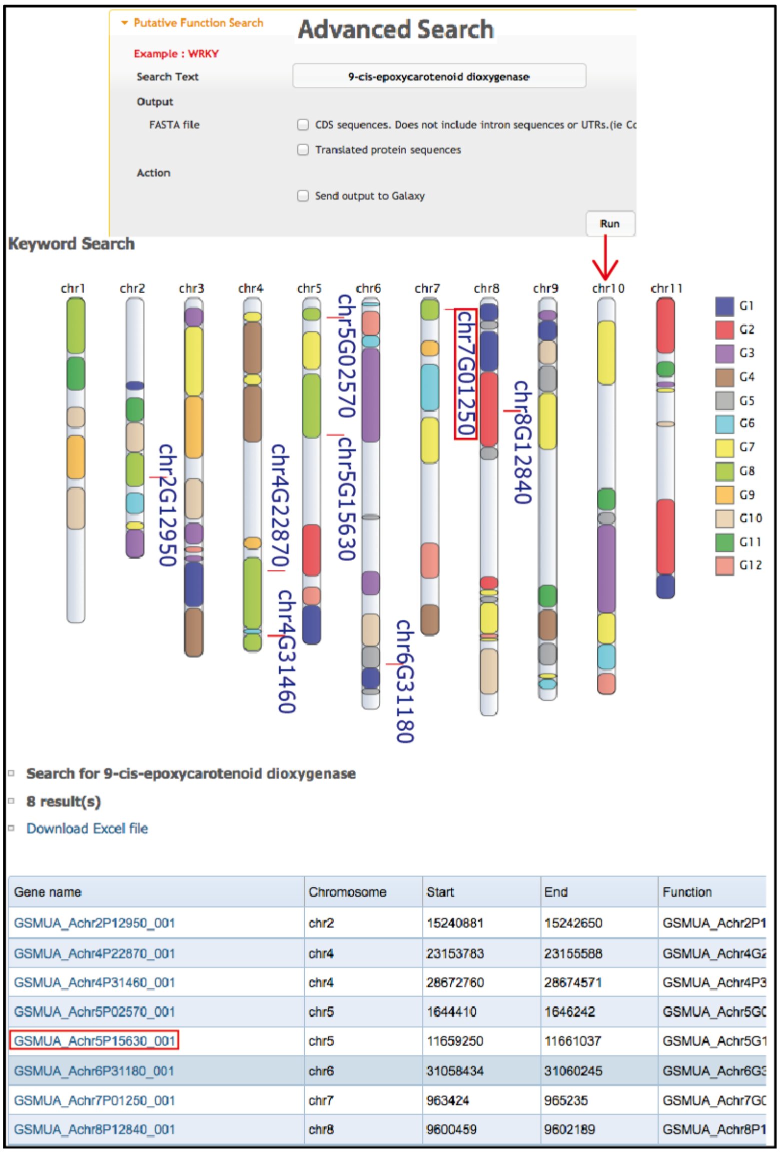

Gene name	Chromosome	Start	End	Function
GSMUA_Achr2P12950_001	chr2	15240881	15242650	GSMUA_Achr2P1
GSMUA_Achr4P22870_001	chr4	23153783	23155588	GSMUA_Achr4G2
GSMUA_Achr4P31460_001	chr4	28672760	28674571	GSMUA_Achr4P3
GSMUA_Achr5P02570_001	chr5	1644410	1646242	GSMUA_Achr5GC
GSMUA_Achr5P15630_001	chr5	11659250	11661037	GSMUA_Achr5G1
GSMUA_Achr6P31180_001	chr6	31058434	31060245	GSMUA_Achr6G3
GSMUA_Achr7P01250_001	chr7	963424	965235	GSMUA_Achr7GC
GSMUA_Achr8P12840_001	chr8	9600459	9602189	GSMUA_Achr8P1

Figure 10: Locate genes on ancestral blocks of *Musa* paralogous regions (Advanced Search).

Method

Banana Genome Hub TOOLS "Advanced Search" allows querying features from attributes (e.g. select gene where product is '9-cis-epoxycarotenoid dioxygenase') in the Bio::DB::SeqFeature::Store MySQL database.
http://banana-genome.cirad.fr/advanced

The resulting features are mapped onto a chromosome drawing where the chromosome are colored according to Musa beta ancestral blocks. This allows quickly checking if paralogs come from tandem duplication or ancestral WGD. The height DH-Pahang Musa NCED genes seem to come from ancestral duplication.

Results

To better understand the evolutionary history of the six *Musa* NCED genes, we investigated the origin and date of the duplications. So, we looked carefully at the paralogous *Musa* region dotplot, the synonymous substitution rate (Ks) and the gene context. The *Musa* beta ancestral block 8 shown on the *Musa* paralogous region karyotype is found duplicated on the *Musa* chromosomes 2, 4, 5 and 7 containing five out of the six NCED genes.

3.2.1. Using ancestral blocks on a dotplot

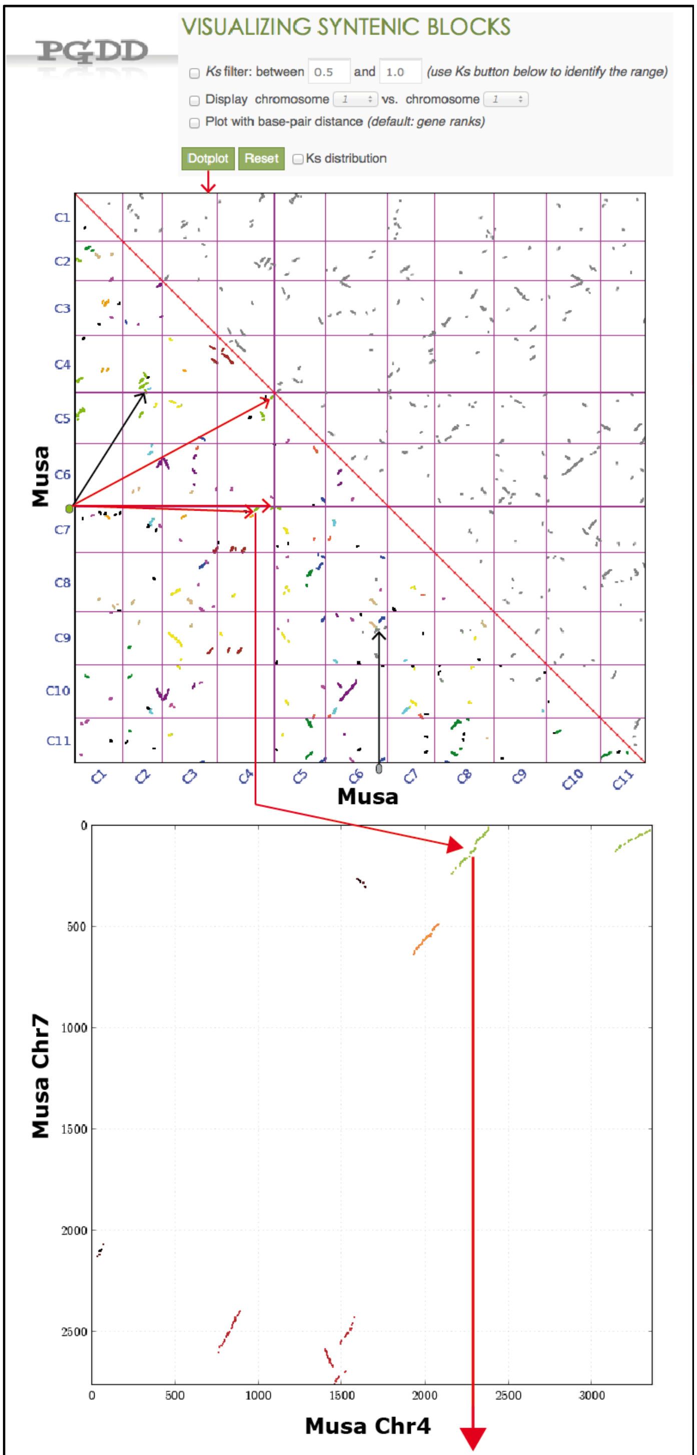

Locus 1	Annotation 1	Locus 2	Annotation 2	Ka	Ks
GSMUA_Achr4P22750_001	Protein YABBY 2	GSMUA_Achr7P01330_001	Protein YABBY 2	0.12	0.44
GSMUA_Achr4P22780_001	Putative Cytosolic 5'-nucleotidase III-like protein	GSMUA_Achr7P01290_001	DnaJ protein homolog	1.08	1.73
GSMUA_Achr4P22820_001	60S ribosomal protein L15	GSMUA_Achr7P01280_001	Putative Ethylene-insensitive protein 2	0.02	0.76
GSMUA_Achr4P22850_001	Cullin-4	GSMUA_Achr7P01270_001	Cullin-4	0.04	0.33
GSMUA_Achr4P22880_001	Hypothetical protein	GSMUA_Achr7P01250_001	9-cis-epoxycarotenoid dioxygenase 1, chloroplastic	1.03	3.33
GSMUA_Achr4P22890_001	Putative Translation initiation factor eIF-2B subunit delta	GSMUA_Achr7P01240_001	Putative Translation initiation factor eIF-2B subunit delta	0.09	0.40
GSMUA_Achr4P22920_001	Hypothetical protein	GSMUA_Achr7P01200_001	Peroxisomal (S)-2-hydroxy-acid oxidase	0.68	1.72
GSMUA_Achr4P22930_001	Hypothetical protein	GSMUA_Achr7P01180_001	CAAX amino terminal protease family protein, putative, expressed	0.66	0.99
GSMUA_Achr4P22940_001	F-box protein At5g46170	GSMUA_Achr7P01170_001	F-box protein At1g30200	0.12	0.47

NCED

Figure 11 Dot plot *Musa* beta ancestral blocks (PGDD).

Method

The Syntenic Doplot link in the Banana Genome Hub TOOLS menu corresponds to the Plant Genome Duplication Database system also allows viewing the synteny results but at a lower scale. http://banana-genome.cirad.fr/dotplot

It allows to clarify the number of block fragment and their location on the chromosomes. Finally, PGDD give access to the list of genes in the syntenic region.

Results

The same ancestral blocks were viewed as macrosynteny in the PGDD DotPlots. It helped to precisely link the relationships between current genes and ancestral blocks. The two pairs of NCED paralogs, (GSMUA_Achr2GP12950_001 and GSMUA_Achr7G01250_001) and (GSMUA_Achr7G01250_001, GSMUA_Achr4G22870_001) were found in syntenic position and resulted from whole genome duplications of beta ancestral blocks. Thus we can say that they are ancient homeologous gene pairs. The last gene of the six *Musa* NCED gene cluster, GSMUA_Achr6G31180_001, belongs to the ancestral block 5 and has the lowest Ks computed with GSMUA_Achr4G31460_001 suggests Zingiberales gamma WGD. Indeed, although the *Musa* NCED Ks are those of gene pairs and are not averages, they are coherent with the nucleotide gene tree topology. Thus, we adjusted the location of the microsynteny region to be viewed in GBrowse.

3.2.2. Checking back NCED gene contexts on the genome browser

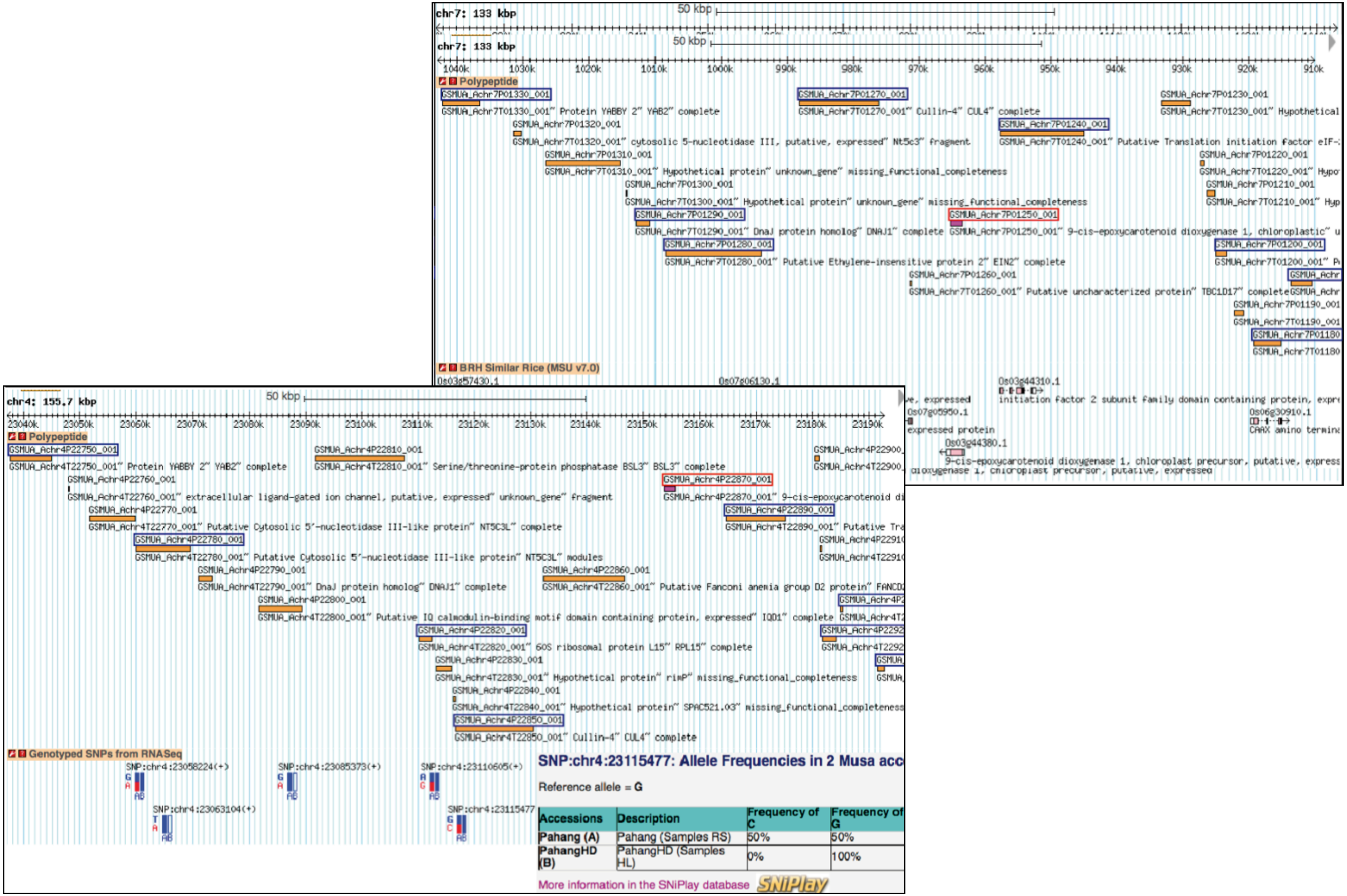
chr7: 133 kbp
50 kbp
Polypeptide
GSMUA_Achr7P01330_001
GSMUA_Achr7T01330_001" Protein YABBY 2" YAB2" complete
GSMUA_Achr7P01270_001
GSMUA_Achr7T01270_001" Cullin-4" CUL4" complete
GSMUA_Achr7P01230_001
GSMUA_Achr7T01230_001" Hypothetical
GSMUA_Achr7P01320_001
GSMUA_Achr7T01320_001" cytosolic 5-nucleotidase III, putative, expressed" Nt5c3" fragment
GSMUA_Achr7P01240_001
GSMUA_Achr7T01240_001" Putative Translation initiation factor eIF-
GSMUA_Achr7P01310_001
GSMUA_Achr7T01310_001" Hypothetical protein" unknown_gene" missing_functional_completeness
GSMUA_Achr7P01300_001
GSMUA_Achr7T01300_001" Hypothetical protein" unknown_gene" missing_functional_completeness
GSMUA_Achr7P01290_001
GSMUA_Achr7T01290_001" DnaJ protein homolog" DNAJ1" complete
GSMUA_Achr7P01250_001
GSMUA_Achr7T01250_001" 9-cis-epoxycarotenoid dioxygenase 1, chloroplastic"
GSMUA_Achr7P01280_001
GSMUA_Achr7T01280_001" Putative Ethylene-insensitive protein 2" EIN2" complete
GSMUA_Achr7P01260_001
GSMUA_Achr7T01260_001" Putative uncharacterized protein" TBC1D17" complete
BRH Similar Rice (MSU v7.0)
9-cis-epoxycarotenoid dioxygenase 1, chloroplast precursor, putative, expressed
chr4: 155.7 kbp
50 kbp
Polypeptide
GSMUA_Achr4P22750_001
GSMUA_Achr4T22750_001" Protein YABBY 2" YAB2" complete
GSMUA_Achr4P22810_001
GSMUA_Achr4T22810_001" Serine/threonine-protein phosphatase BSL3" BSL3" complete
GSMUA_Achr4P22760_001
GSMUA_Achr4T22760_001" extracellular ligand-gated ion channel, putative, expressed" unknown_gene" fragment
GSMUA_Achr4P22870_001
GSMUA_Achr4T22870_001" 9-cis-epoxycarotenoid di
GSMUA_Achr4P22770_001
GSMUA_Achr4T22770_001" Putative Cytosolic 5'-nucleotidase III-like protein" NT5C3L" complete
GSMUA_Achr4P22890_001
GSMUA_Achr4P22780_001
GSMUA_Achr4T22780_001" Putative Cytosolic 5'-nucleotidase III-like protein" NT5C3L" modules
GSMUA_Achr4P22790_001
GSMUA_Achr4T22790_001" DnaJ protein homolog" DNAJ1" complete
GSMUA_Achr4P22860_001
GSMUA_Achr4T22860_001" Putative Fanconi anemia group D2 protein" FANCD2
GSMUA_Achr4P22800_001
GSMUA_Achr4T22800_001" Putative IQ calmodulin-binding motif domain containing protein, expressed" IQD1" complete
GSMUA_Achr4P22820_001
GSMUA_Achr4T22820_001" 60S ribosomal protein L15" RPL15" complete
GSMUA_Achr4P22830_001
GSMUA_Achr4T22830_001" Hypothetical protein" rimP" missing_functional_completeness
GSMUA_Achr4P22840_001
GSMUA_Achr4T22840_001" Hypothetical protein" SPAC521.03" missing_functional_completeness
GSMUA_Achr4P22850_001
GSMUA_Achr4T22850_001" Cullin-4" CUL4" complete
Genotyped SNPs from RNASeq
SNP:chr4:23058224(+)
SNP:chr4:23085373(+)
SNP:chr4:23110605(+)
SNP:chr4:23063104(+)
SNP:chr4:23115477
SNP:chr4:23115477: Allele Frequencies in 2 Musa acc
Reference allele = G
Accessions | Description | Frequency of C | Frequency of G
Pahang (A) | Pahang (Samples RS) | 50% | 50%
PahangHD (B) | PahangHD (Samples HL) | 0% | 100%
More information in the SNiPlay database

Figure 12: Gene context of two *Musa* paralogous regions containing a NCED gene (GBrowse).

Method

Go to the banana Genome Hub GBROWSE tab and search for the two paralogous region.
http://banana-genome.cirad.fr/cgi-bin/gbrowse/musa_acuminata/?name=chr4:23040001..23190000
http://banana-genome.cirad.fr/cgi-bin/gbrowse/musa_acuminata/?name=chr7:1040000..910001

Results

At a lower scale, on the genome browser, we compared the genomic context of GSMUA_Achr7G01250_001 and GSMUA_Achr4G22870_001 we can guess a microsynteny region. Red boxes are for the NCED genes and blue boxes are for syntenic genes whose function is conserved between both paralogous regions. We noticed that the two regions are in opposite orientation.

3.2.3. SNP analysis

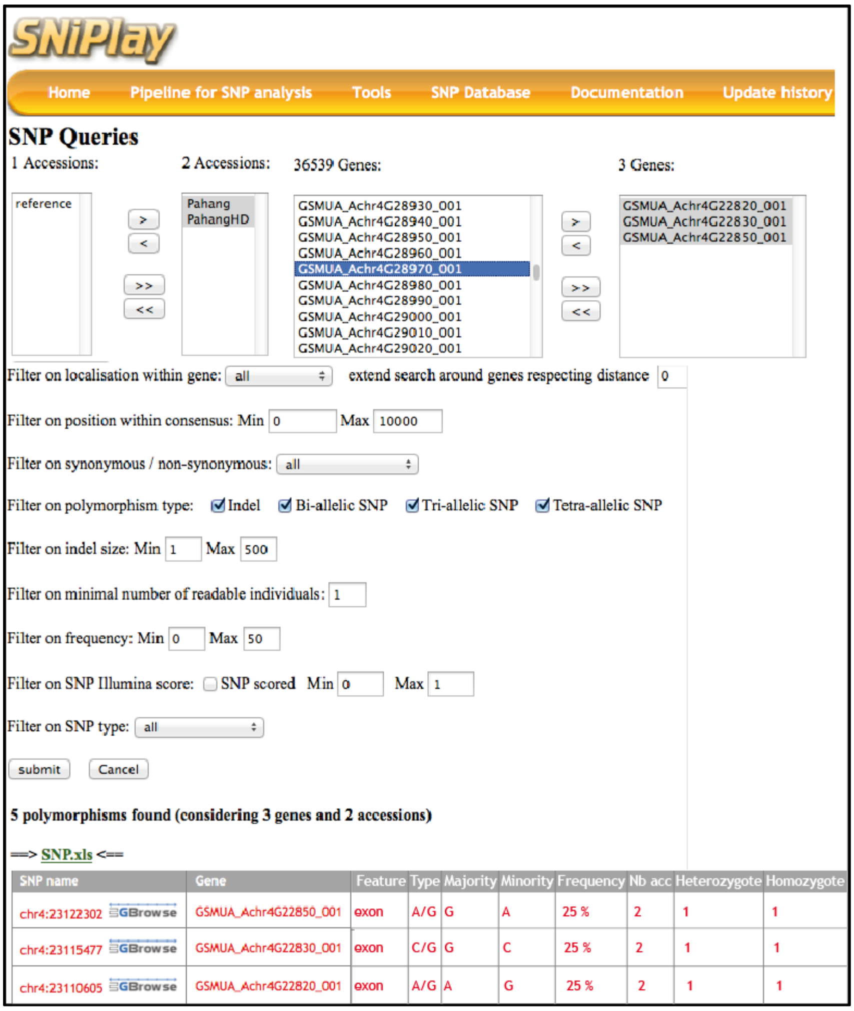

Figure 13: SNP analysis (SNiPlay).

Method

Here, Illumina RNA-seq from DH-Pahang (homozygous) and Pahang (heterozygous) (same data than RNA-seq tracks in GBrowse 2, *i.e.* BLS control and inoculated; FOC TR4 non infected and infected) were mapped and analyzed on the DH-Pahang CDS. A link is made from GBrowse balloon URL using locus tag identifier but one can also query SNiPlay with a custom gene list from the SNP link in the Banana Genome Hub TOOLS menu.
http://banana-genome.cirad.fr/sniplay

Results

Querying SNiPlay, we observed no single nucleotide polymorphism (SNP) in GSMUA_Achr4G22870_001 in the studied conditions but we did in neighbor genes such as the GSMUA_Achr4G22830_001 coding sequence (GC polymorphism).

4. Query builder to export results

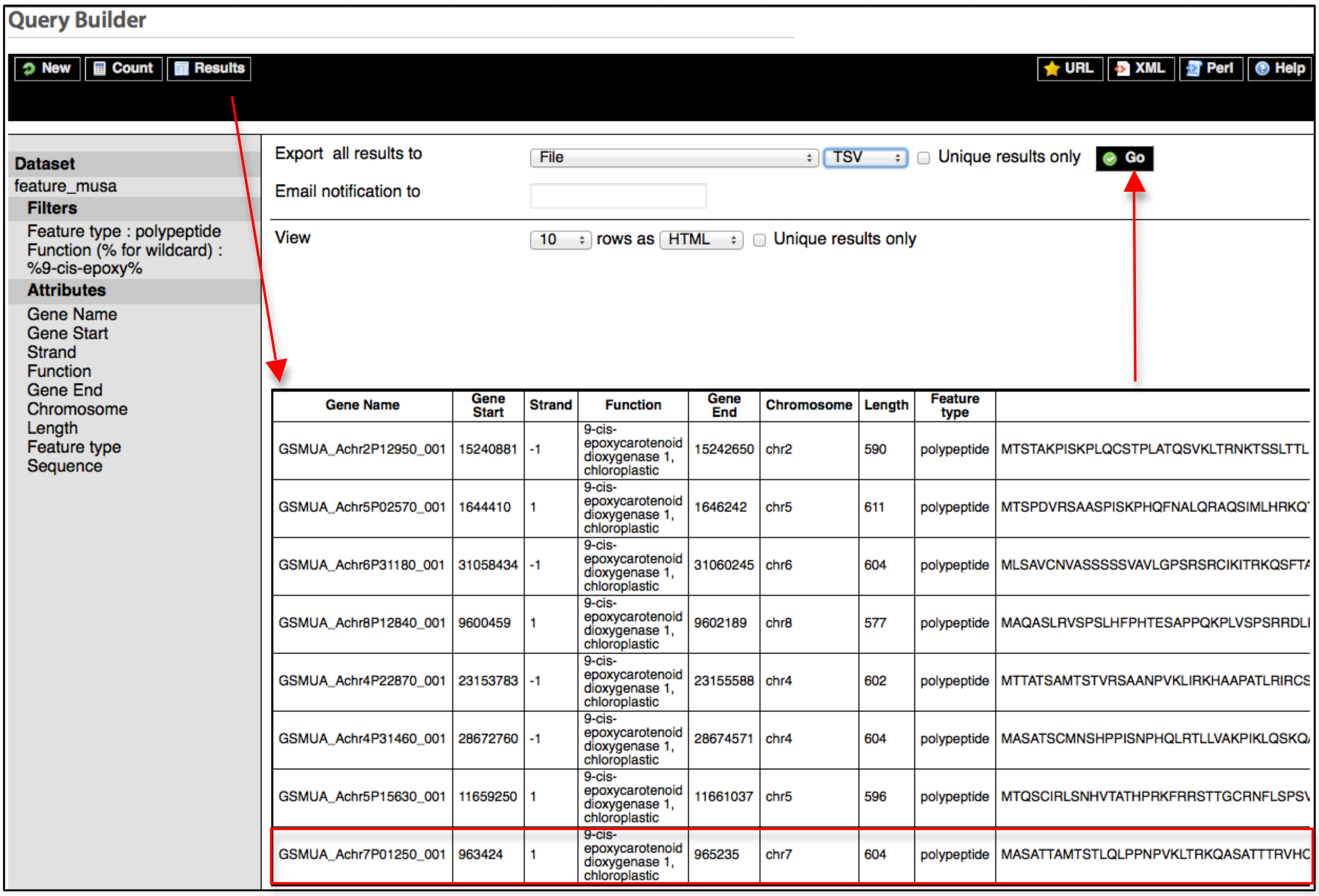

Gene Name	Gene Start	Strand	Function	Gene End	Chromosome	Length	Feature type	
GSMUA_Achr2P12950_001	15240881	-1	9-cis-epoxycarotenoid dioxygenase 1, chloroplastic	15242650	chr2	590	polypeptide	MTSTAKPISKPLQCSTPLATQSVKLTRNKTSSLTTL
GSMUA_Achr5P02570_001	1644410	1	9-cis-epoxycarotenoid dioxygenase 1, chloroplastic	1646242	chr5	611	polypeptide	MTSPDVRSAASPISKPHQFNALQRAQSIMLHRKQ
GSMUA_Achr6P31180_001	31058434	-1	9-cis-epoxycarotenoid dioxygenase 1, chloroplastic	31060245	chr6	604	polypeptide	MLSAVCNVASSSSSVAVLGPSRSRCIKITRKQSFTA
GSMUA_Achr8P12840_001	9600459	1	9-cis-epoxycarotenoid dioxygenase 1, chloroplastic	9602189	chr8	577	polypeptide	MAQASLRVSPSLHFPHTESAPPQKPLVSPSRRDLI
GSMUA_Achr4P22870_001	23153783	-1	9-cis-epoxycarotenoid dioxygenase 1, chloroplastic	23155588	chr4	602	polypeptide	MTTATSAMTSTVRSAANPVKLIRKHAAPATLRIRCS
GSMUA_Achr4P31460_001	28672760	-1	9-cis-epoxycarotenoid dioxygenase 1, chloroplastic	28674571	chr4	604	polypeptide	MASATSCMNSHPPISNPHQLRTLLVAKPIKLQSKQ
GSMUA_Achr5P15630_001	11659250	1	9-cis-epoxycarotenoid dioxygenase 1, chloroplastic	11661037	chr5	596	polypeptide	MTQSCIRLSNHVTATHPRKFRRSTTGCRNFLSPS
GSMUA_Achr7P01250_001	963424	1	9-cis-epoxycarotenoid dioxygenase 1, chloroplastic	965235	chr7	604	polypeptide	MASATTAMTSTLQLPPNPVKLTRKQASATTTRVHC

Figure 14 Build a query (Biomart).

Method

Biomart has its own database extracted from the Chado PostgreSQL database. It accessible via the Query Builder link in the Banana Genome Hub TOOLS menu.

http://banana-genome.cirad.fr/biomart

Results

The 8 NCED polypeptides can be easily exported all at once, in various formats (*e.g.* tabulated text, fasta), building a query with BioMart where the product (function) contains '9-cis-epoxy' for instance.
