# Supplementary material for: The Banana Genome Hub
Source: Database (Oxford). 2013 May 23;2013:bat035. doi: 10.1093/database/bat035 (PMC3662865; doi:10.1093/database/bat035)
Supplement: Supplementary Data [file supp_bat035_suppl_data.zip › Supplementary Tables.pdf]

SUPPLEMENTARY TABLES

Supplementary Table 1 : Banana Hub Systems hosted on the South Green Bioinformatics Platform

System	Component	Version	Language	Description	References	URL
**Musa Hub**	Tripal, Gbrowse	2.4	PHP	Banana genome reference		http://banana-genome.cirad.fr
**GNPAnnot**	Chado	1.2	PostgreSQL	Generic Model Organism database (GMOD)	(1)	http://www.gmod.org
	Chado Controller	1.5	PostgreSQL Perl Java	Manage user access rights on feature Annotation inspector Annotation history	(2)	http://www.gnpannot.org/content/chado-controller
	GBrowse	2.4	Perl	GMOD Genome Browser	(3)	http://gnpannot.musagenomics.org/cgi-bin/gbrowse/musa/
	Artemis	13.2.8	Java Web Start	Genome Editor	(4)	http://www.sanger.ac.uk/resources/software/artemis/
	Tripal		php	Chado front end with Drupal CMS	(5)	http://tripal.sourceforge.net/
	Web Portal		php	Drupal CMS	(6)	http://www.gnpannot.org/
	GNPAnnot pipeline		C, Perl	Protein coding gene and repetitive element prediction		n/a
	REPET pipeline		Python	Repetitive element prediction		http://urgi.versailles.inra.fr/Tools/REPET
**GreenPhylDB**	Website	3.0	Perl, Javascript (jquery, Raphael) Java applets MySQL	Protein clusters ing, curated gene families, gene trees, ortholog predictions	(7, 8)	http://www.greenphyl.org/cgi-bin/index.cgi
	Pipeline	3.0	Perl, java, C	Gene trees, homolog predictions	(7, 8)	n/a
**OryGenesDB**	Website		Perl, Jquery		(9, 10)	http://orygenesdb.cirad.fr/
**SNiPlay**				SNP predictions	(11)	http://sniplay.cirad.fr/cgi-bin/home.cgi
**ESTtik**	Website		Perl, MySQL	ESTs and RNA-seq	(12)	http://esttik.cirad.fr/

	Pipeline		Perl, C			*n/a*
**TropGENEDB**	Website		Java	Molecular markers, genetic maps and genetic resources	(*13*)	http://tropgenedb.cirad.fr/tropgene/JSP/index.jsp
**Galaxy**	Website	1.2.4	Python	Workflow manager	(*14, 15*)	http://gohelle.cirad.fr/galaxy

Supplementary Table 2 : Cellulose synthase and cellulose synthase-like gene numbers

	Arabidopsis thaliana	*Vitis vinifera*	*Oryza sativa*	*Sorghum bicolor*	***Musa acuminata***
Cellulose synthase (*CesA*)	10	11	10	10	**16**
Cellulose synthase-like (*Csl*)					
CslA	9	4	11	8	**14**
CslB	6	7	0	0	**0**
CslC	5	5	6	5	**12**
CslD	6	5	5	5	**9**
CslE	1	9	3	3	**3**
CslF	0	0	8	9	**0**
CslG	3	15	0	0	**4**
CslH	0	0	3	3	**1**
CslJ	0	3	0	1	**0**

Supplementary Table 3 : Summary of Musa CCD proteins identified by GreenPhylDB and analyzed through the banana hub. Chloroplat peptides were predicted with TargetP (*16*).

Locus tag	Family (Greenphyl ID)	Protein length	Chloro peptide	EST reads	Genotyped SNP	Nearest genetic marker
GSMUA_Achr2 P09410_001	GP074574 CCD8 type	567	Yes	AFFK454ReadsT00082726001 AFFK454ReadsT00082727001 AFFK454ReadsT00082728001 AFFK454ReadsT00082729001		mMaCIR1160
GSMUA_Achr2 P12950_001	GP069973 NCED	590	Yes	AFFM454ReadsT00070926001, AFFM454ReadsT00070925001, AFFM454ReadsT00070924001		mMaCIR1179
GSMUA_Achr3 P29770_001	GP072303 CCD1 type	548	No	Around 50 EST reads		mMaCIR1387
GSMUA_Achr4 P19020_001	GP069973 CCD4 type	527	Yes	7 EST reads		mMaCIR1045
GSMUA_Achr4 P22870_001	GP069973 NCED	602	Yes	none		mMaCIR1054
GSMUA_Achr4 P31460_001	GP069973 NCED	604	Yes	AFFN454ReadsT00031438001 AFFN454ReadsT00031439001 AFFN454ReadsT00031437001 AFFN454ReadsT00031436001		mMaCIR1405
GSMUA_Achr5 P02570_001	GP069973 NCED	548	Yes	None	BESCIRAD13770_5 BESCIRAD13770_3	mMaCIR1072
GSMUA_Achr5 P15630_001	GP069973 NCED	596	Yes	AFFK454ReadsT00054272001		mMaCIR1588
GSMUA_Achr6 P26310_001	GP074574 CCD8 type	557	Yes	none		mMaCIR1470
GSMUA_Achr6 P31180_001	GP069973 NCED	604	Yes	AFFI454ReadsT00005131001		mMaCIR0352
GSMUA_Achr7 P01250_001	GP069973 NCED	604	Yes	none		mMaCIR1230
GSMUA_Achr8 P12840_001	GP069973 NCED	576	Yes	none		mMaCIR2313
GSMUA_Achr1 1P21000_001	GP003862 CCD7 type	615	Yes	none		mMaCIR1092

Supplementary Table 4 : Summary of Musa SPS proteins identified by GreenPhylDB and analyzed through the banana hub. Chloroplat peptides were predicted with TargetP (*16*).

Locus tag	Family (Greenphyl ID)	Protein length	Chloro peptide	EST reads	Genotyped SNP	Nearest genetic marker
GSMUA_Achr9 P22510_001	GP016032 SPS Subfamily	1062	No	More than 50	GAPR00017878001 GAPR00017879001 GAPR00017880001 GAPR00017881001 BESCNS1573_5 GAPR00017882001 GAPR00004504001	mMeCIR525 mMaCI1634
GSMUA_Achr4 P06050_001	GP016032 SPS Subfamily	1060	No	AFFI454ReadsT00060836001 AFFH454ReadsT00110683001 AFFH454ReadsT00110682001 AFFI454ReadsT00060837001 AFFL454ReadsT00055556001 AFFI454ReadsT00060838001	1	mMaCIR1326 mMaCIR1122
GSMUA_Achr4 P16070_001	GP016032 SPS Subfamily	1083	No	More than 30	BESCNS4059_5 And 3 others	mMaCIR1745 mMaCIR1191
GSMUA_Achr6 P17480_001	GP016032 SPS Subfamily	1044	No	AFFI454ReadsT00000065001 AFFI454ReadsT00000066001 AFFN454ReadsT00000024001 AFFL454ReadsT00000034001		mMaCIR1653 mMaCIR1131

References

1. Mungall, C. J., and Emmert, D. B. (2007) A Chado case study: an ontology-based modular schema for representing genome-associated biological information, *Bioinformatics 23*, i337-346.
2. Guignon, V., Droc, G., Alaux, M., Baurens, F. C., Garsmeur, O., Poiron, C., Carver, T., Rouard, M., and Bocs, S. (2012) Chado Controller: advanced annotation management with a community annotation system, *Bioinformatics*.
3. Stein, L. D., Mungall, C., Shu, S., Caudy, M., Mangone, M., Day, A., Nickerson, E., Stajich, J. E., Harris, T. W., Arva, A., and Lewis, S. (2002) The generic genome browser: a building block for a model organism system database, *Genome Res 12*, 1599-1610.
4. Carver, T., Berriman, M., Tivey, A., Patel, C., Bohme, U., Barrell, B. G., Parkhill, J., and Rajandream, M. A. (2008) Artemis and ACT: viewing, annotating and comparing sequences stored in a relational database, *Bioinformatics 24*, 2672-2676.
5. Ficklin, S. P., Sanderson, L. A., Cheng, C. H., Staton, M. E., Lee, T., Cho, I. H., Jung, S., Bett, K. E., and Main, D. (2011) Tripal: a construction toolkit for online genome databases, *Database (Oxford) 2011*, bar044.
6. Guignon, V., Droc, G., and Bocs, S. (2008-2011) GNPAnnot portal http://www.gnpannot.org.
7. Conte, M., Gaillard, S., Lanau, N., Rouard, M., and Perin, C. (2008) GreenPhylDB: a database for plant comparative genomics, *Nucleic acids research 36*, D991-D998.
8. Rouard, M., Guignon, V., Aluome, C., Laporte, M. A., Droc, G., Walde, C., Zmasek, C. M., Périn, C., and Conte, M. G. (2011) GreenPhylDB v2. 0: comparative and functional genomics in plants, *Nucleic acids research 39*, D1095-D1102.
9. Droc, G., Périn, C., Fromentin, S., and Larmande, P. (2009) OryGenesDB 2008 update: database interoperability for functional genomics of rice, *Nucleic acids research 37*, D992-D995.
10. Droc, G., Ruiz, M., Larmande, P., Pereira, A., Piffanelli, P., Morel, J., Dievart, A., Courtois, B., Guiderdoni, E., and Perin, C. (2006) OryGenesDB: a database for rice reverse genetics, *Nucleic acids research 34*, D736-D740.
11. Dereeper, A., Nicolas, S., Le Cunff, L., Bacilieri, R., Doligez, A., Peros, J. P., Ruiz, M., and This, P. (2011) SNiPlay: a web-based tool for detection, management and analysis of SNPs. Application to grapevine diversity projects, *BMC bioinformatics 12*, 134.
12. Argout, X., Ruiz, M., Fouet, O., Lanaud, C., Wincker, P., Da Silva, C., and Courtois, B. ESTtik, a semi-automatic cDNA sequence analysis and annotation pipeline including SSR and SNP search tools.
13. Ruiz, M., Rouard, M., Raboin, L. M., Lartaud, M., Lagoda, P., and Courtois, B. (2004) TropGENE-DB, a multi-tropical crop information system, *Nucleic acids research 32*, D364-D367.
14. Afgan, E., Ananda, G., Baker, D., Blankenberg, D., Bouvier, D., Clements, D., Coraor, N., Goecks, J., Hillman Jackson, J., von Kuster, G., Lazarus, R., Nekrutenko, A., and Taylor, J. (2012) Galaxy Bioinformatics workflow management systems https://main.g2.bx.psu.edu/, Galaxy is funded by National Science Foundation, Eberly College of Science, and Huck Institutes for the Life Sciences.

15. Vincent Maillol, R. B., Stéphanie Sidibe Bocs, Jean-Michel Boursiquot, Grégory Carrier, Alexis Dereeper, Gaétan Droc, Cécile Fleury, Pierre Larmande, Loïc Lecunff, Jean-Pierre Péros, Bertrand Pitollat, Manuel Ruiz, Gautier Sarah, Guilhem Sempéré, Marilyne Summo, Patrice This, and Jean-Francois Dufayard. (2012) Role of Galaxy in a bioinformatic plant breeding platform, In *Galaxy Community Conference*.
16. Emanuelsson, O., Nielsen, H., Brunak, S., and von Heijne, G. (2000) Predicting subcellular localization of proteins based on their N-terminal amino acid sequence, *Journal of molecular biology 300*, 1005-1016.
