# Supplementary material for: The Banana Genome Hub
Source: Database (Oxford). 2013 May 23;2013:bat035. doi: 10.1093/database/bat035 (PMC3662865; doi:10.1093/database/bat035)
Supplement: Supplementary Data [file supp_bat035_suppl_data.zip › Supplementary File 1.pdf]

Case Study of the Banana SPS Gene Family

Table of contents

1. Search for the *Musa* SPS Gene Family 2

2. Structural Annotation 4

2.1. Genes 4
2.2. Molecular Markers 8

3. Functional Annotation 10

3.1. Gene Report 10
3.2. Gene Families 13
3.3. Metabolic Pathways 15
3.1. Transcriptome Expression 17

4. Phylogenetic and Paralogous Region Analyses 19

4.1. Phylogenomics System 19
4.2. Phylogeny Refinement 23
4.3. Synteny of Paralogous Musa Regions 30

Figure 1: Genes search (Advance Search). 4
Figure 2: Genome browser (GBrowse). 6
Figure 3: Annotation editor (Artemis). 8
Figure 4: Genetic Map (CMap). 10
Figure 5: Gene report. 11
Figure 6: Sucrose-phosphate synthase family (GreenPhylDB). 15
Figure 7: Enzyme Sucrose-phosphate synthase, reaction 2.4.1.14 and pathway sucrose biosynthesis (Pathway Tools / MusaCyc). 17
Figure 8: EST report (ESTtik). 19
Figure 9: Phylogenomics system (GreenphylDB) 21
Figure 10: Phylogenetic trees (GreenphylDB) 23
Figure 11: Upload data to the workflow manager (Galaxy) 25
Figure 12: Editing a workflow (Galaxy) 27
Figure 13: Running a workflow (Galaxy) 29
Figure 14: Phylogenetic trees (Galaxy) 29
Figure 15: Dotplot of paralogous *Musa* regions coloured according to Musa ancestral blocks (PGDD) 32

In this document, we provide an overview of the functionalities of the Banana Genome Hub. For this purpose we selected a specific gene family to support the description. We studied the Sucrose phosphate synthase (SPS) family of the *Musa acuminata* double-haploid Pahang proteome using several information systems available from the Banana Genome Hub. *M. acuminata* is a giant herb of the monocotyledon class.

To start with the Banana Genome Hub, go to http://banana-genome.cirad.fr/

1. Search for the *Musa* SPS Gene Family

The advanced research tool is available in the "Tools" tab of the BananaGeome Hub.

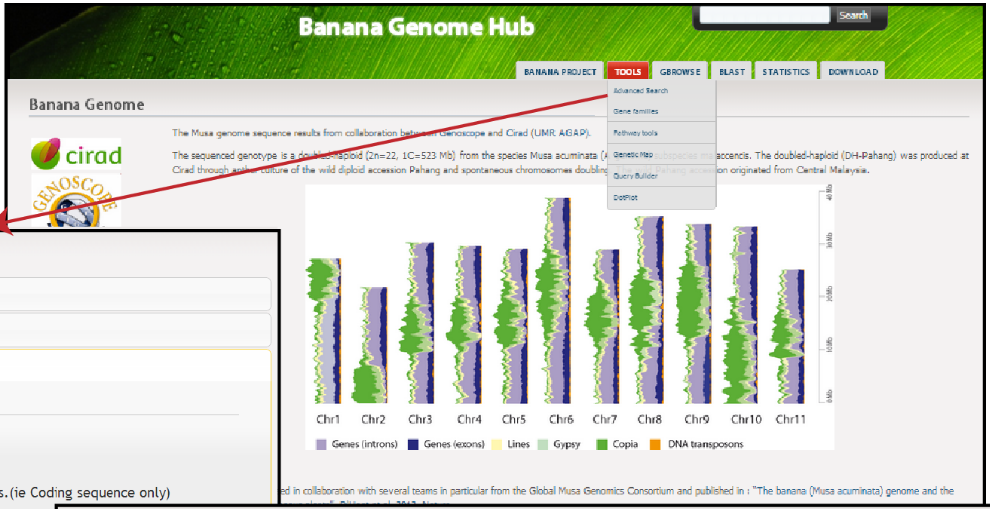

The research can be made from several ways.

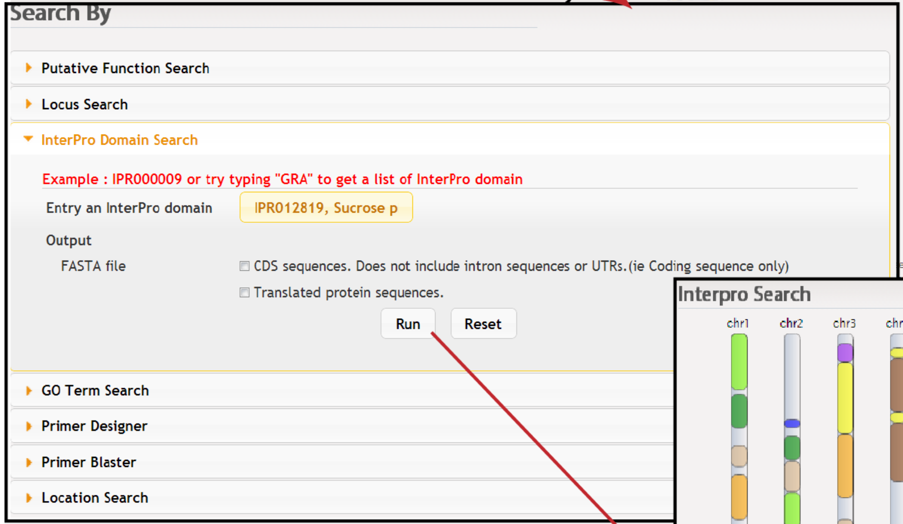

Results are given as a list of gene with a karyotype showing the synthenic blocks and the position of gene

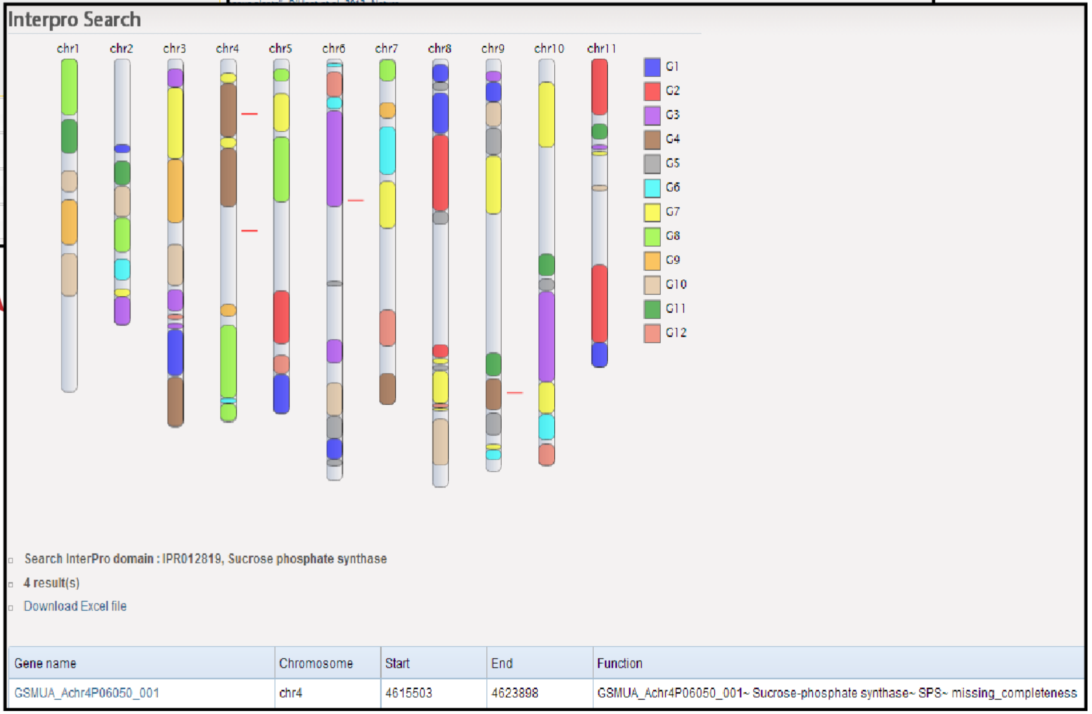

Figure 1: Genes search (Advance Search).

Method

Click on Advanced Search located in the dropdown menu TOOLS of the main menu.

http://banana-genome.cirad.fr/advanced

Gene retrieval can be performed using keywords, Locus, InterPro domains, GO Terms or genome location. Access the search field by clicking desired type of search. The loci of the resulting genes are located on the karyotypes and listed in a table with their genome location along the chromosome as well as their polypeptide product. To study the SPS, we searched for the InterPro IPR012819 corresponding to the Sucrose-phosphate synthase domain. By clicking on the name of one of the resulting gene (*e.g.* GSMUA_Achr4P06050_001) it leads to the genome browser, GBrowse.

Results

The four genes corresponding to this search are:

- GSMUA_Achr4P16070_001
- GSMUA_Achr4P06050_001
- GSMUA_Achr6P17480_001
- GSMUA_Achr9P22510_001

2. Structural Annotation

2.1. Genes

To analyze the structure of these genes, it is possible through the Community Annotation System (CAS) accessible via the dropdown menu TOOLS (http://banana-genome.cirad.fr/gene_annotation).

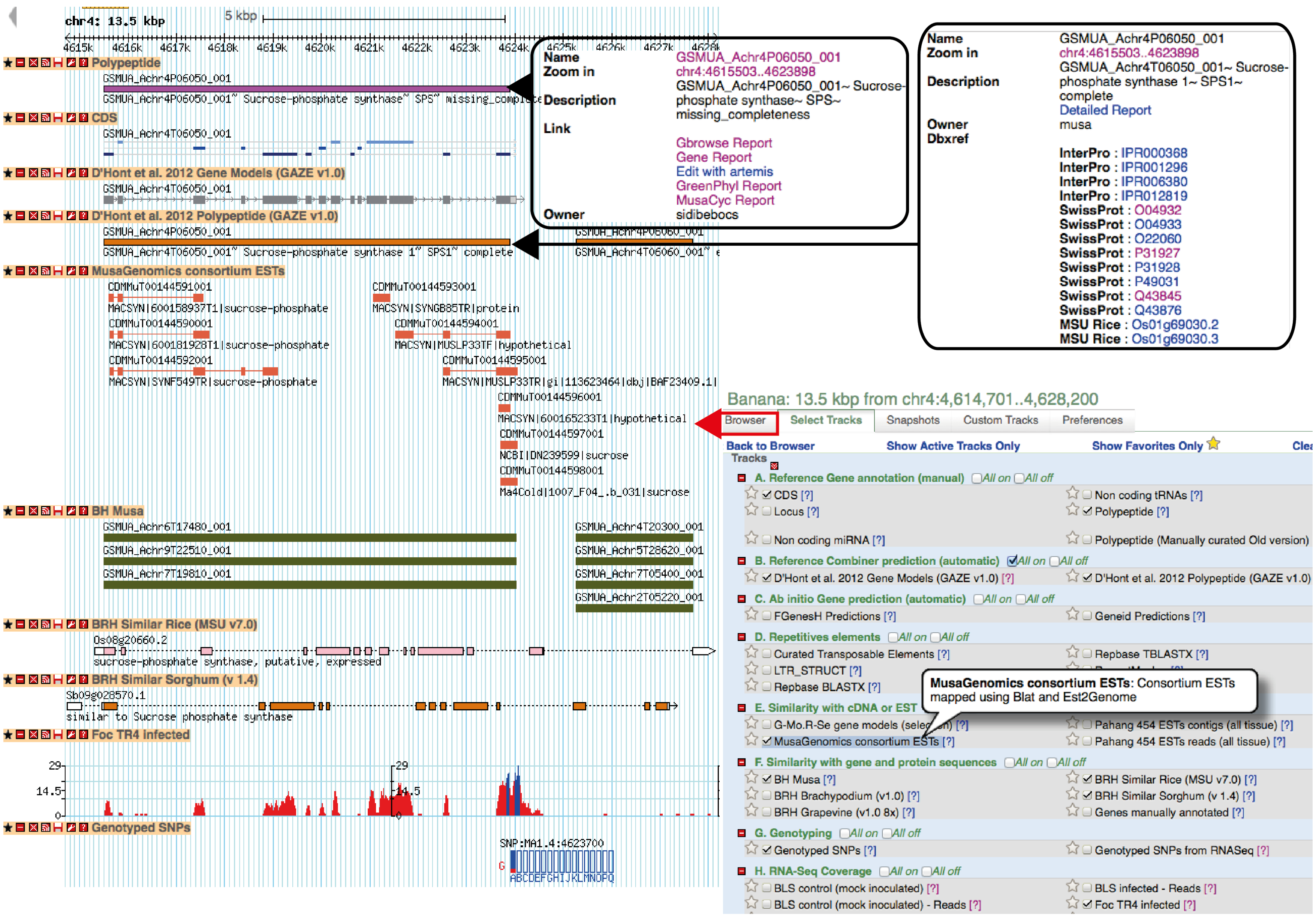

chr4: 13.5 kbp
5 kbp
Polypeptide
GSMUA_Achr4P06050_001
CDS
GSMUA_Achr4T06050_001
D'Hont et al. 2012 Gene Models (GAZE v1.0)
D'Hont et al. 2012 Polypeptide (GAZE v1.0)
MusaGenomics consortium ESTs
BH Musa
BRH Similar Rice (MSU v7.0)
sucrose-phosphate synthase, putative, expressed
BRH Similar Sorghum (v 1.4)
similar to Sucrose phosphate synthase
Foc TR4 infected
Genotyped SNPs
Name
Zoom in
Description
Link
Owner
GSMUA_Achr4P06050_001
chr4:4615503..4623898
GSMUA_Achr4P06050_001~ Sucrose-phosphate synthase~ SPS~ missing_completeness
Gbrowse Report
Gene Report
Edit with artemis
GreenPhyl Report
MusaCyc Report
sidibebocs
Name
Zoom in
Description
Owner
Dbxref
GSMUA_Achr4T06050_001~ Sucrose-phosphate synthase 1~ SPS1~ complete
Detailed Report
musa
InterPro : IPR000368
InterPro : IPR001296
InterPro : IPR006380
InterPro : IPR012819
SwissProt : O04932
SwissProt : O04933
SwissProt : O22060
SwissProt : P31927
SwissProt : P31928
SwissProt : P49031
SwissProt : Q43845
SwissProt : Q43876
MSU Rice : Os01g69030.2
MSU Rice : Os01g69030.3
Banana: 13.5 kbp from chr4:4,614,701..4,628,200
Browser
Select Tracks
Snapshots
Custom Tracks
Preferences
Back to Browser
Show Active Tracks Only
Show Favorites Only
Tracks
A. Reference Gene annotation (manual)
B. Reference Combiner prediction (automatic)
C. Ab initio Gene prediction (automatic)
D. Repetitives elements
E. Similarity with cDNA or EST
F. Similarity with gene and protein sequences
G. Genotyping
H. RNA-Seq Coverage
MusaGenomics consortium ESTs: Consortium ESTs mapped using Blat and Est2Genome

Figure 2: Genome browser (GBrowse).

Method

The Banana Genome Browser is accessible through links in various locations, (i) the most obvious is from the GBROWSE tab on the bar menu of the Banana Genome Hub, but (ii) it can be accessed by clicking on the name of a gene resulting from the Advanced Search.

(i) Search by name: you go to http://banana-genome.cirad.fr/cgi-bin/gbrowse/musa_acuminata/ then you search for GSMUA_Achr4P06050_001.

(ii) Search by region: you go directly to
http://banana-genome.cirad.fr/cgi-bin/gbrowse/musatract_tripal/?name=chr4:4615503..4623898

The choice of displayed tracks is available on the «Select Tracks» tab. Information about each feature is available via the question mark. By clicking on the tracks you can display more information and external links.

Looking at GSMUA_Achr4P06050_001 in the genome browser allows checking its intron/exon structure. Indeed, GBrowse provides information about the genomic region (chr4:4,615,503..4,623,898) and can aggregate various types of data such as similarities with EST, polypeptide sequences. The available information can be customized by checking/unchecking in track boxes. By clicking on any track feature, a pop up displays more information about the feature and provides external links such as cross references to other databases.

http://www.ebi.ac.uk/interpro/DisplayIproEntry?ac=IPR012819
http://www.uniprot.org/uniprot/P31927
http://rice.plantbiology.msu.edu/cgi-bin/ORF_infopage.cgi?orf=LOC_Os08g20660.1
http://www.uniprot.org/uniprot/Q6ZHZ1

In the case of Reference Gene annotation (manual) track, the feature pop up provides a link to several tools of the Banana Genome Hub such as Artemis annotation editor (Figure 3) and gene reports (GBrowse; Tripal, Figure 5; GreenPhylDB, Figure 6 and Musacyc, Figure 7).

Results

In this case, we compared the automatic prediction of genes (D'Hont et al. 2012 Gene Models (GAZE v1.0) box of the Reference Combiner prediction (automatic) track) with the manual gene annotation (CDS box of the Reference Gene annotation (manual) track) and similarities with other genes and proteins (Best Hits, Reciprocal BH). The annotation comparison will allow to quickly checked whether the structure of the predicted gene (GSMUA_Achr4P06050_001 chr4:4,615,503..4,623,898) is correct. Structural modifications of size large enough should be visible in the Reference Gene annotation (manual) track. We can see by comparing those tracks, that exon 11 and 12 of GSMUA_Achr4P06050_001 predicted by GAZE were manually merged. These exons were merged for all of the four *Musa* SPS genes. The visualization of the GBrowse report shows that the annotation has been made by our team (e.g. owner: sidibebocs).

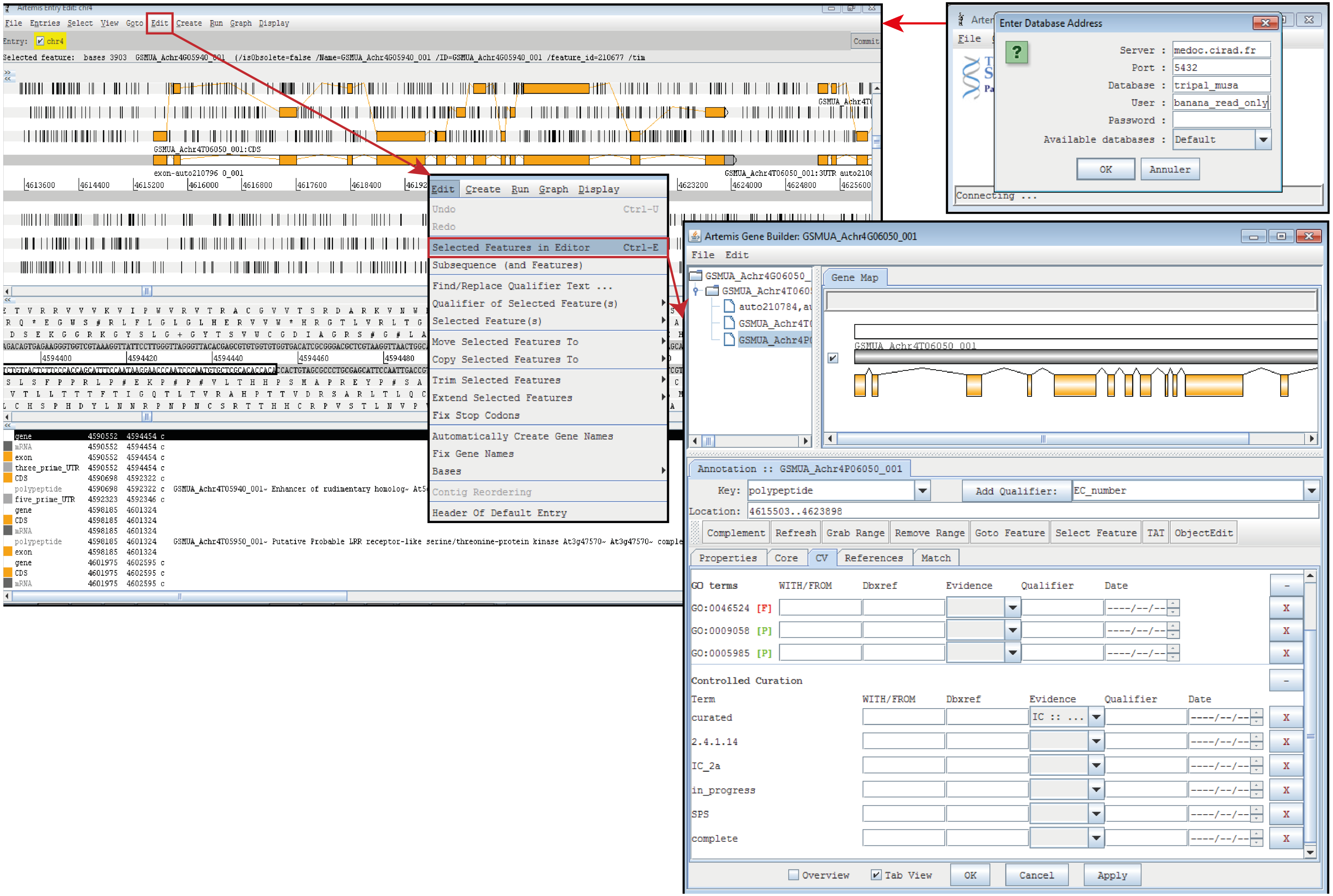
Enter Database Address
Server : medoc.cirad.fr
Port : 5432
Database : tripal_musa
User : banana_read_only
Password :
Available databases : Default
OK
Annuler
Connecting ...
Edit Create Run Graph Display
Undo Ctrl-U
Redo
Selected Features in Editor Ctrl-E
Subsequence (and Features)
Find/Replace Qualifier Text ...
Qualifier of Selected Feature(s)
Selected Feature(s)
Move Selected Features To
Copy Selected Features To
Trim Selected Features
Extend Selected Features
Fix Stop Codons
Automatically Create Gene Names
Fix Gene Names
Bases
Contig Reordering
Header Of Default Entry
Artemis Gene Builder: GSMUA_Achr4G06050_001
File Edit
Gene Map
Annotation :: GSMUA_Achr4P06050_001
Key: polypeptide
Add Qualifier: EC_number
Location: 4615503..4623898
Complement Refresh Grab Range Remove Range Goto Feature Select Feature TAT ObjectEdit
Properties Core CV References Match
GO terms WITH/FROM Dbxref Evidence Qualifier Date
GO:0046524 [F]
GO:0009058 [P]
GO:0005985 [P]
Controlled Curation
Term WITH/FROM Dbxref Evidence Qualifier Date
curated
2.4.1.14
IC_2a
in_progress
SPS
complete
Overview Tab View OK Cancel Apply

Figure 3: Annotation editor (Artemis).

Method

To edit a gene, you need to launch Artemis via the “Edit with Artemis” link in the GBrowse pop up of a banana gene or polypeptide or in the Gene Report (visible in figure 2).

http://banana-genome.cirad.fr/cgi-bin/artemis.pl?species=musa;name=chr4:4590502..4648898

There is two ways to use the annotation tool: if you have an account, you can participate to the community annotation system by modifying features stored into the Chado Database. If you do not have an account, you can ask an account (see http://banana-genome.cirad.fr/content/annotation-account-request on the http://banana-genome.cirad.fr/gene_annotation accessible in the TOOLS tab of the Banana Genome Hub Web site) or connect to Artemis with a read-only account: banana_read_only without password and modifications can be saved on your local computer.

Once Artemis is open, you can visualize the area around the gene of interest. By clicking on the Edit tab and then on the “Select Feature on the Editor” part, you can access the Artemis gene builder. In this builder you can access information such as comments of the curators that annotated this gene. Clicking on the commit button calls the Chado Controller annotation inspector module. The annotation Inspector validation window will appear. Clicking OK will commit the modifications whereas clicking the upper right cross will rollback to the original state. The changes are stored into the Chado database. To have more information about how to annotate a gene structure, see the Protocol for structural gene annotation and the Chado Controller publication.

http://banana-genome.cirad.fr/documentation

http://www.gnpannot.org/biblio

Like in GBrowse, you can have access the cross references for other databases, in the References tab of the gene builder.

Results

As mentioned before, we can see in the Artemis entry edit window that exons 11 and 12 of GSMUA_Achr4G16070_001 were merged.

In the annotator_comment qualifier of the Core tab of the gene builder, another correction is indicated: “one GC / AG intron-exon junction between exons 5 and 6” (exon five was extended from 14258178 to 14258199). This modification took into account the rare splicing site, GC, whereas the GAZE combiner predicted instead the classical GT.

2.2. Molecular Markers

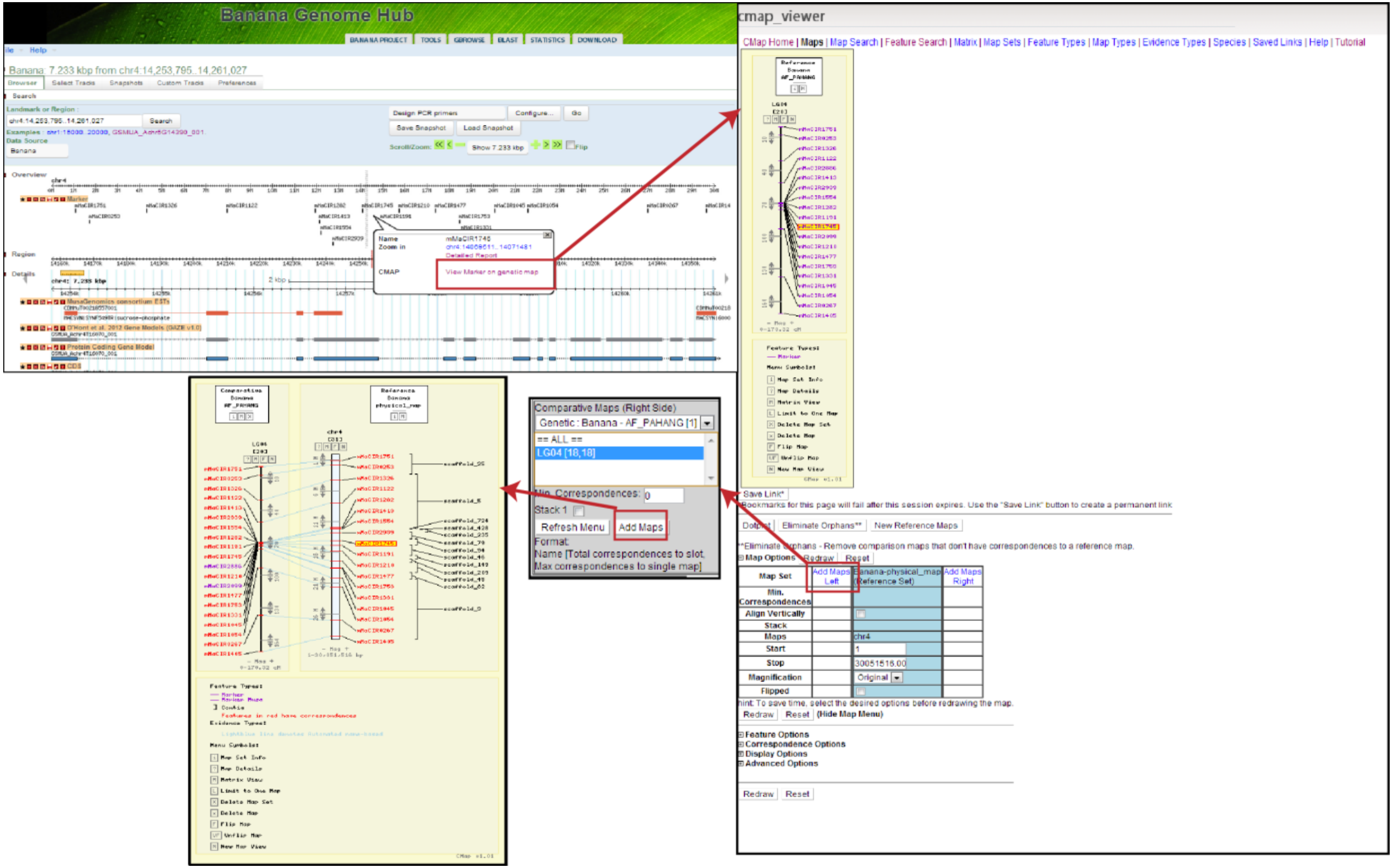
Banana Genome Hub
BANANA PROJECT | TOOLS | GBROWSE | BLAST | STATISTICS | DOWNLOAD
Banana: 7.233 kbp from chr4:14,253,795..14,261,027
View Marker on genetic map
cmap_viewer
CMap Home | Maps | Map Search | Feature Search | Matrix | Map Sets | Feature Types | Map Types | Evidence Types | Species | Saved Links | Help | Tutorial
Comparative Maps (Right Side)
Genetic : Banana - AF_PAHANG [1]
== ALL ==
LG04 [18,18]
Min. Correspondences: 0
Stack 1
Refresh Menu
Add Maps
Format:
Name [Total correspondences to slot, Max correspondences to single map]
Eliminate Orphans**
New Reference Maps
Map Options
Redraw
Reset
Map Set
Add Maps Left
Banana-physical_map (Reference Set)
Add Maps Right
Min. Correspondences
Align Vertically
Stack
Maps
chr4
Start
1
Stop
30051516.00
Magnification
Original
Flipped
Feature Options
Correspondence Options
Display Options
Advanced Options

Figure 4: Genetic Map (CMap).

Method

In order to get information about the genetic environment of the genes, we studied the nearest markers of the genes from the pop-up of the marker track on the GBrowse by clicking on "View marker on genetic map".

http://banana-genome.cirad.fr/cmap_viewer?ref_map_set_acc=108;ref_map_accs=3150;highlight=111183;comparative_maps=1=map_acc=AF_PAHANG_LG4

CMAP provides a physical map of the markers, and via an addition of map, links it to the genetic map ("Add a map" link).

A marker in CMap is linked to the Banana GBrowse and TropGeneDB

http://banana-genome.cirad.fr/cgi-bin/gbrowse/musa_acuminata/?name=mMaCIR1745

http://tropgenedb.cirad.fr/tropgene/JSP/link.jsp?module=BANANA&value=mMaCIR1745&tref=marker&cref=name&tabIndex=0&onglet=MAPS&numero=10

Results

We can see in this case that GSMUA_Achr4P16070_001 is in a region with a low recombination rate in the vicinity of the centromere. The closest actual marker mMaCIR1745 (14069511..14071481) is located at 182 kb in 5' of GSMUA_Achr4G16070_001. The type of this marker is microsatellite, also known as Simple Sequence Repeats (SSRs). Quantitative trait locus (QTL) information generated on banana could be added when available, providing additional clues on putative function of the genes.

3. Functional Annotation

Knowing the structure of the genes of the SPS gene Family in Musa, we wanted to learn more about their function.

3.1. Gene Report

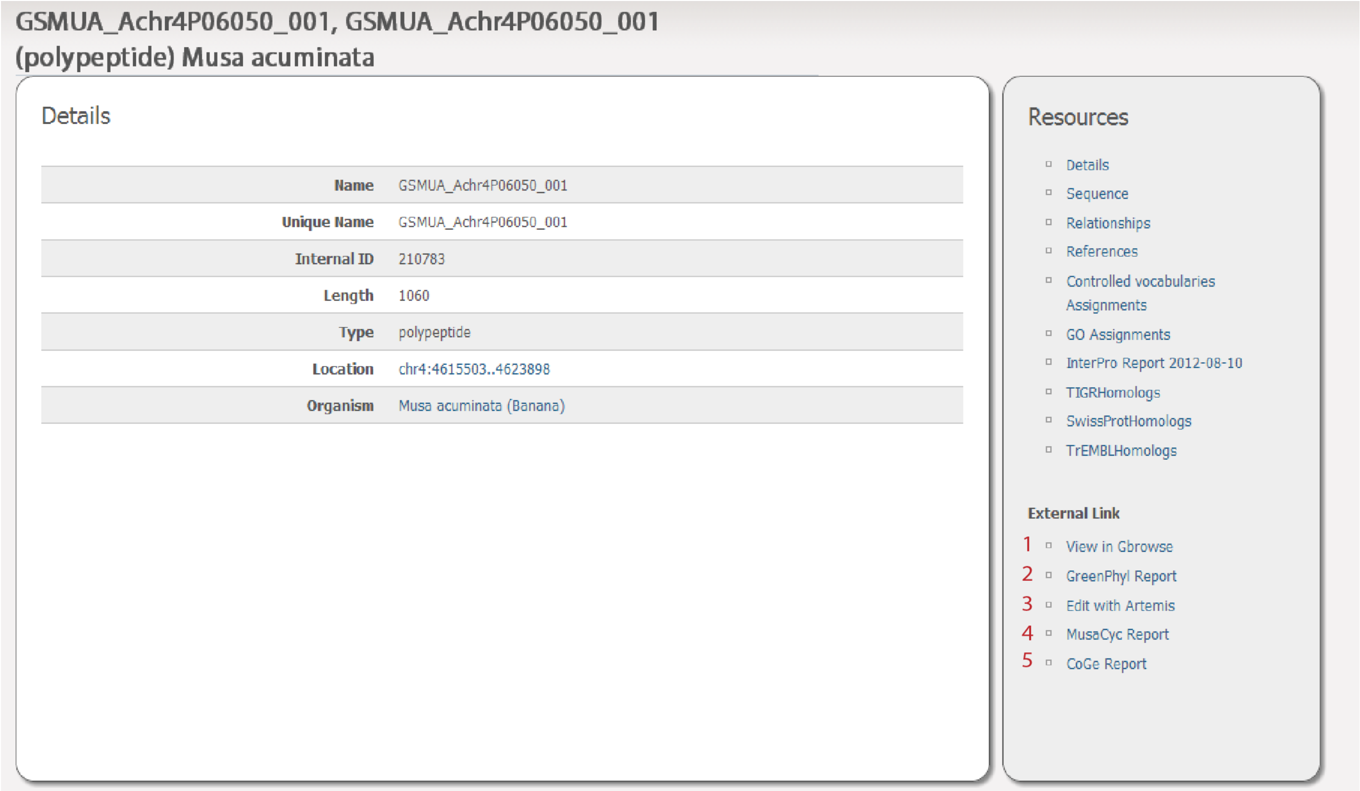

1 - Return to gene visualisation on Gbrowse
2 - Link to Gene informations on Greenphyl
3 - Edition of the gene with the Artemis Software
4 - Link to the MusaCyc tool giving information about the pathway the gene are implied in
5 - Link to the CoGe tool

Figure 5: Gene report.

Method

To get detailed information about a banana gene, mRNA or polypeptide, the Gene Report (Tripal) is accessible through the quick search above the bar menu (in the banner) of the Banana Genome Hub (search for GSMUA_4P06050_001) and also via the “Gene Report” link in the GBrowse pop up of a gene or polypeptide (Figure 2).
http://banana-genome.cirad.fr/GSMUA_4P06050_001
The gene report provides resources about the protein-coding genes:

- Details : General information : Name, ID, length, type, Location, Organism
- Sequence : Sequence on fasta format
- Relationship : Other features linked in the Banana Genome Hub
- References : cross references to other external databases
- Controlled vocabularies Assignments
- GO Assignment
- InterPro Report : Links to Interpro Domains
- TIGRHomologs : Best Blast hits against MSU (ex-TIGR) Database
- SwissProtHomologs : Best Blast hits against SwissProt Database
- TrEMBLHomologs : Best Blast hits against TrEMBL Database

External links are also provided to lead to several tools giving complementary information about our gene of interest (GBrowse, Figure 2; Greenphyl, Figure 6; Artemis, Figure 3; MusaCyc, Figure 7, CoGe).

Results

Tableau 1 General functional information about the search results.

Name	Length	Location	InterPro Domains	GO Assignment
GSMUA_Achr4P06050_001	1060	chr4:4615503..4623898	IPR000368	GO:0009058
			IPR001296	GO:0005985
			IPR006380	GO:0046524
			IPR012819	
GSMUA_Achr4P16070_001	1083	chr4:14253795..14261027	IPR000368	GO:0009058
			IPR001296	GO:0005985
			IPR006380	GO:0046524
			IPR012819	
GSMUA_Achr9P22510_001	1062	chr9:27608557..27620605	IPR001296	GO:0009058
			IPR006380	GO:0005985
			IPR012819	GO:0046524
GSMUA_Achr6P17480_001	1044	chr6:11756040..11760311	IPR001296	GO:0009058
			IPR006380	GO:0005985
			IPR012819	GO:0046524

Three InterPro domains are common to all genes:

- IPR001296 : Glycosyl transferase, group 1
- IPR003680 : Sucrose-phosphate synthase
- IPR012819 : Sucrose phosphate synthase, plant

An additional InterPro domain was found only in GSMUA_Achr4P16070_001 and GSMUA_Achr9P22510_001: IPR000368 Sucrose synthase.

The GO Assignments were the same for the four genes:

- GO:0009058 : biosynthetic process
- GO:0005985 : sucrose metabolic process
- GO:0046524 : sucrose-phosphate synthase activity

The controlled vocabulary is available on the CV tab of the Artemis Gene Builder. To have more information about how to annotate a polypeptide function, see the Protocol for functional gene annotation.
http://banana-genome.cirad.fr/documentation

3.2. Gene Families

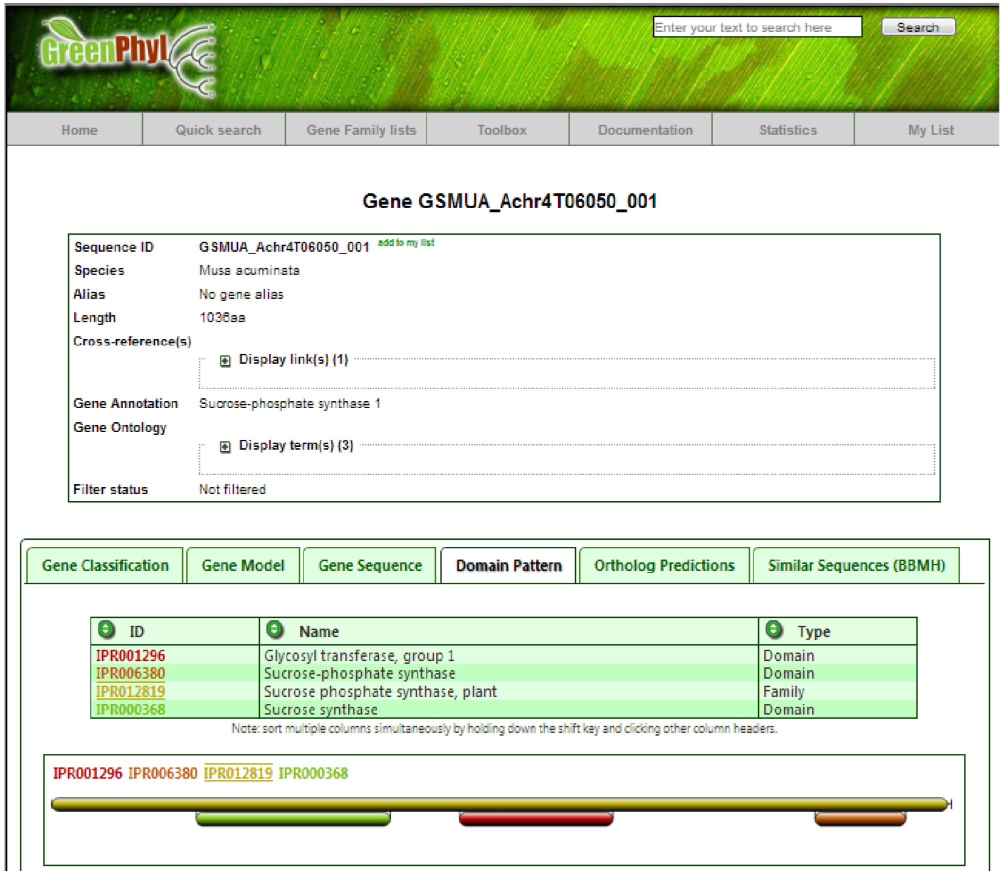

ID	Name	Type
IPR001296	Glycosyl transferase, group 1	Domain
IPR006380	Sucrose-phosphate synthase	Domain
IPR012819	Sucrose phosphate synthase, plant	Family
IPR000368	Sucrose synthase	Domain

Figure 1: IPR domains for GSMUA_Achr4T06050_001

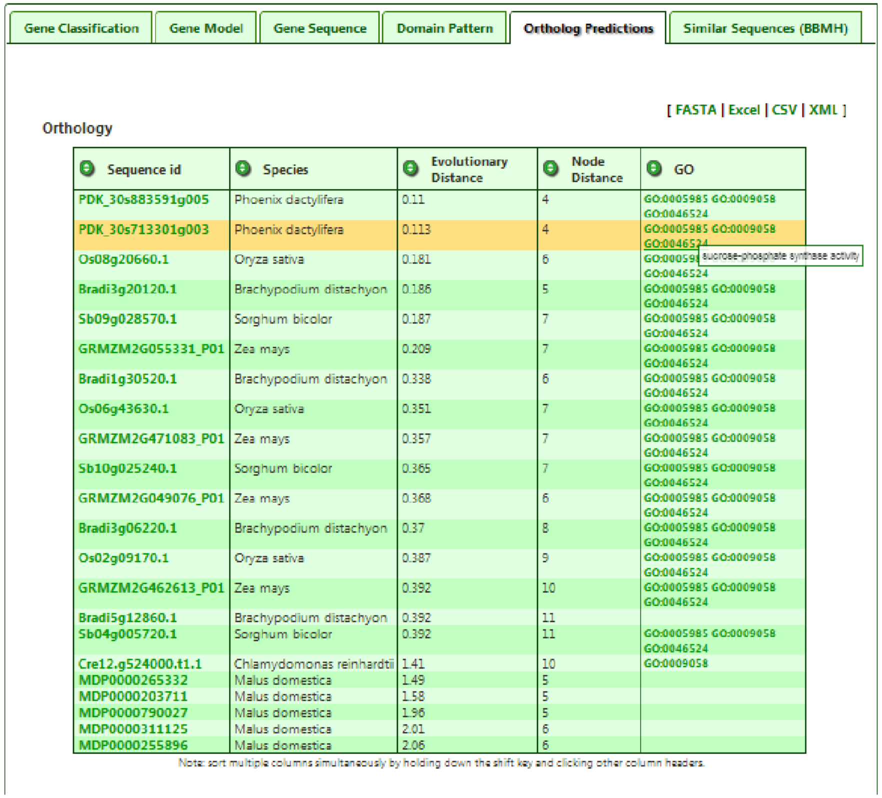

Sequence id	Species	Evolutionary Distance	Node Distance	GO
PDK_30s883591g005	Phoenix dactylifera	0.11	4	GO:0005985 GO:0009058 GO:0046524
PDK_30s713301g003	Phoenix dactylifera	0.113	4	GO:0005985 GO:0009058 GO:0046524
Os08g20660.1	Oryza sativa	0.181	6	GO:00059[illegible] GO:0046524
Bradi3g20120.1	Brachypodium distachyon	0.186	5	GO:0005985 GO:0009058 GO:0046524
Sb09g028570.1	Sorghum bicolor	0.187	7	GO:0005985 GO:0009058 GO:0046524
GRMZM2G055331_P01	Zea mays	0.209	7	GO:0005985 GO:0009058 GO:0046524
Bradi1g30520.1	Brachypodium distachyon	0.338	6	GO:0005985 GO:0009058 GO:0046524
Os06g43630.1	Oryza sativa	0.351	7	GO:0005985 GO:0009058 GO:0046524
GRMZM2G471083_P01	Zea mays	0.357	7	GO:0005985 GO:0009058 GO:0046524
Sb10g025240.1	Sorghum bicolor	0.365	7	GO:0005985 GO:0009058 GO:0046524
GRMZM2G049076_P01	Zea mays	0.368	6	GO:0005985 GO:0009058 GO:0046524
Bradi3g06220.1	Brachypodium distachyon	0.37	8	GO:0005985 GO:0009058 GO:0046524
Os02g09170.1	Oryza sativa	0.387	9	GO:0005985 GO:0009058 GO:0046524
GRMZM2G462613_P01	Zea mays	0.392	10	GO:0005985 GO:0009058 GO:0046524
Bradi5g12860.1	Brachypodium distachyon	0.392	11	
Sb04g005720.1	Sorghum bicolor	0.392	11	GO:0005985 GO:0009058 GO:0046524
Cre12.g524000.t1.1	Chlamydomonas reinhardtii	1.41	10	GO:0009058
MDP0000265332	Malus domestica	1.49	5	
MDP0000203711	Malus domestica	1.58	5	
MDP0000790027	Malus domestica	1.96	5	
MDP0000311125	Malus domestica	2.01	6	
MDP0000255896	Malus domestica	2.06	6	

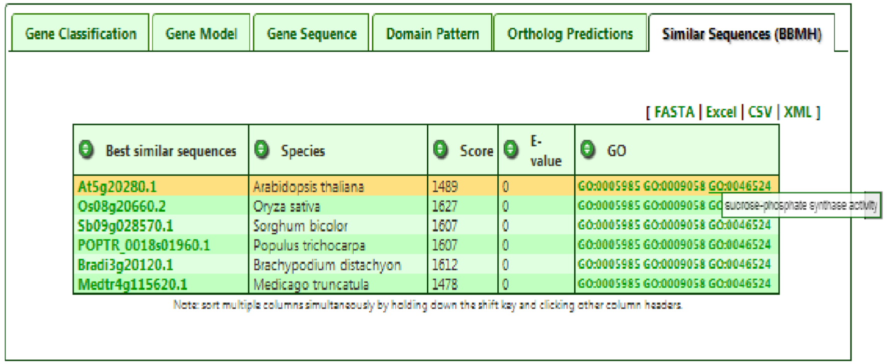

Best similar sequences	Species	Score	E-value	GO
At5g20280.1	Arabidopsis thaliana	1489	0	GO:0005985 GO:0009058 GO:0046524
Os08g20660.2	Oryza sativa	1627	0	GO:0005985 GO:0009058 GO[illegible]
Sb09g028570.1	Sorghum bicolor	1607	0	GO:0005985 GO:0009058 GO:0046524
POPTR_0018s01960.1	Populus trichocarpa	1607	0	GO:0005985 GO:0009058 GO:0046524
Bradi3g20120.1	Brachypodium distachyon	1612	0	GO:0005985 GO:0009058 GO:0046524
Medtr4g115620.1	Medicago truncatula	1478	0	GO:0005985 GO:0009058 GO:0046524

Figure 6: Sucrose-phosphate synthase family (GreenPhylDB).

Method

In order to study further the SPS gene family, we clicked on the GreenPhyl gene report accessible from GBrowse or the Gene report trough the "GreenPhyl Report" link.
http://www.greenphyl.org/cgi-bin/sequence.cgi?p=id&seq_textid=GSMUA_Achr4T06050_001

From the GreenPhyl report, we can access to the InterPro Domains and their Location, to BBMH and to orthologs prediction. For more see Figure 9 and Figure 10.

Results

The observation of the BBMH and the Orthologs confirmed the functional annotation of our genes. Indeed, the GO:0046524 sucrose Phosphatase activity is present in all of them, as well as InterPro IPR006380 Sucrose-phosphate synthase.

3.3. Metabolic Pathways

Search Database *Musa acuminata* chan

Home | Search | Tools | Help | Gene

Musa acuminata Enzyme: Sucrose-phosphate sy

Protein Sequence | Nucleotide Sequence | Nucleotide Sequence, Ad

Gene: GSMUA_Achr4P06050_001 Accession Number: GSMUA_ACHR4P06050_001

Map Position: [4,615,503 -> 4,623,898] (15.36 centisomes) on Chromosome chr4 Genor

Unification Links: Pmid:16876912 , Tigr:Os01g69030.2 , Tigr:Os01g69030.3 , Tigr:Os01g , Tigr:Os02g09170.2 , Tigr:Os06g43630.1 , Tigr:Os08g20660.1 , Tigr:Os08g20660.2 Trembl:C8TEV8 , Trembl:D6R6Y6 , Trembl:Q2ABX9 , Trembl:Q6SXU0 , Trembl:Q6UL Trembl:Q8H1Y1 , UniProtKB:O04932 , UniProtKB:O04933 , UniProtKB:O22060 , UniPr UniProtKB:P31928 , UniProtKB:P49031 , UniProtKB:Q43845 , UniProtKB:Q43876

Relationship Links: InterPro:IN-FAMILY:IPR000368 , InterPro:IN-FAMILY:IPR001296 , Inte InterPro:IN-FAMILY:IPR012819

Gene-Reaction Schematic:

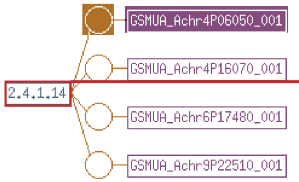

GO Terms:

Biological Process:	GO:0005985 - sucrose metabolic process<br>GO:0009058 - biosynthetic process
Molecular Function:	GO:0046524 - sucrose-phosphate synthase activity

MultiFun Terms: UNCLASSIFIED

Enzymatic reaction of: sucrose-phosphate synthase (Sucrose-phosphate s

D-fructose-6-phosphate + UDP-D-glucose <=> sucrose-6-phosphate + uridine-5'-diphosphat

The reaction direction shown, that is, A + B <==> C + D versus C + D <==> A + B, is in acc Commission system.

The reaction is favored in the direction shown.

In Pathways: sucrose biosynthesis

Musa acuminata Reaction: 2.4.1.14

Species Comparison

Superclasses: Reactions-Classified-By-Conversion-Type -> Simple-Reactions -> Chemical-Reactions -> EC-Reactions -> 2 -- Transferases -> 2.4 -- Glycosyltransferases -> 2.4.1 -- Hexosyltransferases
Reactions-Classified-By-Substrate -> Small-Molecule-Reactions

Enzymes and Genes:
Sucrose-phosphate synthase : GSMUA_Achr9P22510_001
Sucrose-phosphate synthase : GSMUA_Achr6P17480_001
Sucrose-phosphate synthase : GSMUA_Achr4P16070_001
Sucrose-phosphate synthase : GSMUA_Achr4P06050_001

In Pathway: sucrose biosynthesis

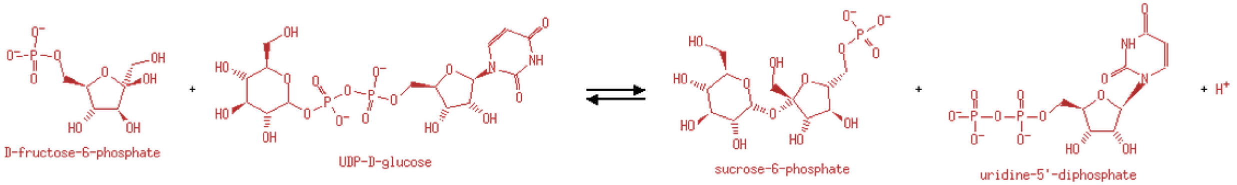

The reaction direction shown, that is, A + B <==> C + D versus C + D <==> A + B, is in accordance with the Enzyme Commission system.

Most BioCyc compounds have been protonated to a reference pH value of 7.3, and some reactions have been computationally balanced for hydrogen by adding free protons. Please see the BioCyc Guide for more information.

Enzyme Commission Primary Name for this Reaction: Sucrose-phosphate synthase

Quick Search | Gene Search
Search Database *Musa acuminata* change

Home | Search | Tools | Help | Pathway

Musa acuminata Pathway: sucrose biosynthesis

Show Predicted Enzymes | More Detail | Less Detail | Species Comparison

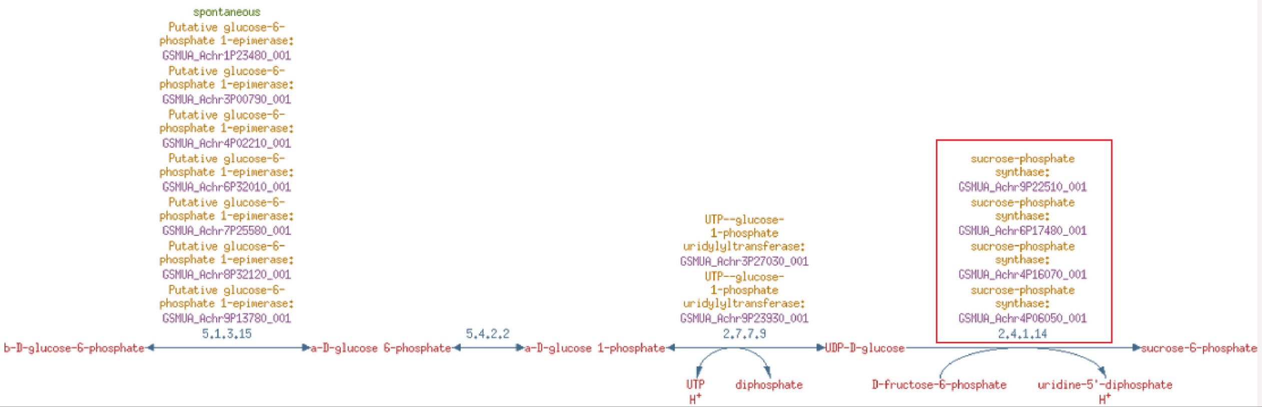

Figure 7: Enzyme Sucrose-phosphate synthase, reaction 2.4.1.14 and pathway sucrose biosynthesis (Pathway Tools / MusaCyc).

Method

MusaCyc displays information about all enzymes implied in the reaction and also in the pathway. It provides biological information about the reaction and the pathway. In order to get information about the metabolic pathways of the SPS gene family in plants, we clicked on the "MusaCyc Report" link available on GBrowse and the gene report.

http://banana-genome.cirad.fr/musacyc_report?GSMUA_Achr4P06050_001

As in other systems, you can have access to cross references to other databases, in the "Unification Links" and the "Relationship Links" paragraphs. If you click on the Enzyme Commission (EC) number (here 2.1.4.14), you can also have cross references to the BRENDA and ENZYME databases.

http://enzyme.expasy.org/EC/2.4.1.14

By clicking on the Enzymatic reaction, you can have access to the catalyzing step of sucrose biosynthesis from UDP-glucose and fructose- 6-phosphate.

http://gohelle.cirad.fr:1555/MUSA/NEW-IMAGE?type=REACTION&object=SUCROSE-PHOSPHATE-SYNTHASE-RXN

By clicking on the Pathway, you can have access to the sucrose bioisynthesis pathway.

http://gohelle.cirad.fr:1555/MUSA/NEW-IMAGE?type=PATHWAY&object=SUCSYN-PWY

Results

The EC number 2.1.4.14 was predicted for GSMUA_Achr4P06050_001. It corresponds to the enzyme named the Sucrose-phosphate synthase catalyzing the last reaction of the sucrose biosynthesis pathway.

The four SPS polypeptides (GSMUA_Achr4P16070_001, GSMUA_Achr4P06050_001, GSMUA_Achr6P17480_001 and GSMUA_Achr9P22510_001) have an enzymatic activity characterized by the EC number 2.1.4.14.

3.1. Transcriptome Expression

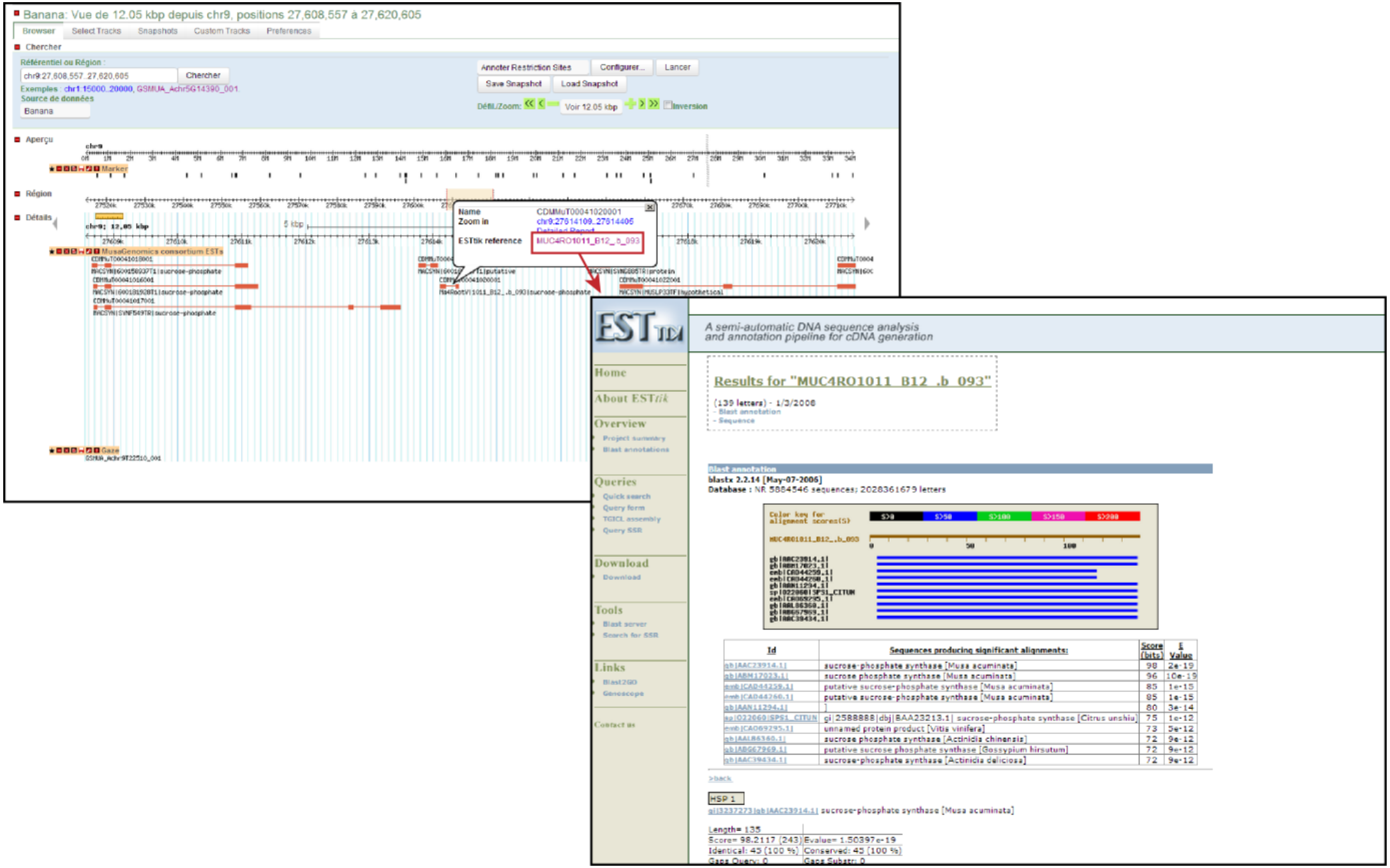
Banana: Vue de 12.05 kbp depuis chr9, positions 27,608,557 à 27,620,605
Browser
Select Tracks
Snapshots
Custom Tracks
Preferences
Chercher
Référentiel ou Région :
chr9:27,608,557..27,620,605
Chercher
Exemples : chr1:15000..20000, GSMUA_Achr5G14390_001
Source de données
Banana
Annoter Restriction Sites
Configurer...
Lancer
Save Snapshot
Load Snapshot
Défil./Zoom:
Voir 12.05 kbp
Inversion
Aperçu
Région
Détails
Marker
MusaGenomics consortium ESTs
Gaze
Name
CDMMuT00041020001
Zoom in
chr9:27614109..27614405
ESTtik reference
MUC4RO1011_B12_.b_093
A semi-automatic DNA sequence analysis and annotation pipeline for cDNA generation
Home
About ESTtik
Overview
Queries
Download
Tools
Links
Contact us
Results for "MUC4RO1011_B12_.b_093"
(139 letters) - 1/3/2008
Blast annotation
blastx 2.2.14 [May-07-2006]
Database : NR 5884546 sequences; 2028361679 letters
Id
Sequences producing significant alignments:
Score (bits)
E Value
gb|AAC23914.1| sucrose-phosphate synthase [Musa acuminata] 98 2e-19
gb|ABM17023.1| sucrose phosphate synthase [Musa acuminata] 96 10e-19
emb|CAD44259.1| putative sucrose-phosphate synthase [Musa acuminata] 85 1e-15
emb|CAD44260.1| putative sucrose-phosphate synthase [Musa acuminata] 85 1e-15
gb|AAN11294.1| 80 3e-14
sp|O22060|SPS1_CITUN gi|2588888|dbj|BAA23213.1| sucrose-phosphate synthase [Citrus unshiu] 75 1e-12
emb|CAO69295.1| unnamed protein product [Vitis vinifera] 73 5e-12
gb|AAL86360.1| sucrose phosphate synthase [Actinidia chinensis] 72 9e-12
gb|ABG67969.1| putative sucrose phosphate synthase [Gossypium hirsutum] 72 9e-12
gb|AAC39434.1| sucrose-phosphate synthase [Actinidia deliciosa] 72 9e-12
HSP 1
gi|3237273|gb|AAC23914.1| sucrose-phosphate synthase [Musa acuminata]
Length= 135
Score= 98.2117 (243) Evalue= 1.50397e-19
Identical: 45 (100 %) Conserved: 45 (100 %)
Gaps Query: 0 Gaps Subst: 0

Figure 8: EST report (ESTtik).

Method

Evidence of expression is sometimes available with ESTs from ESTtik system. You can go to ESTtik from the “ESTtik reference” link present in the GBrowse pop up of some EST of the track MusaGenomics consortium ESTs (group E. Similarity with cDNA or EST).

http://esttik.cirad.fr/cgi-bin/public_quick_search.cgi?name_seq=MUC4RO1011_B12_.b_093&type=singleton&project=MUC4_ROOTINVITRO&clean=cleaned&navigate=2&species=MusaESTtik

ESTtik report provides information about ESTs such as the sequence, the blast annotation and the database from which is issued the EST.

Results

In this case, the EST MUC4RO1011_B12_.b_093 matches with GSMUA_AChr9T22510_001. As described in the detailed report, the EST has been reported on roots, that confirm the expression of this gene in *Musa* roots. Moreover the best blastx hit with nr databank is a *Musa* polypeptide (gb|AAC23914.1 in ESTtik Report) encoded by a CDS (GenBank:U59489.1) that has a *Musa* reference by Nascimento,J.R.O. et al. 1997 (PMID:9431676). They said:

“A 952-base pair polymerase-chain-reaction product of sucrose-phosphate synthase (SPS) (EC 2.3.1.14) from banana (Musa acuminata cv. Nanicão) fruit was cloned and used to study expression of the enzyme during development and ripening. The deduced amino acid sequence shows that banana SPS has a high similarity with the leaf, tap-root and bean enzymes from other species. Enzyme activity, and mRNA and protein levels point to an increase in SPS expression during ripening. The accumulation of sucrose was correlated to starch degradation and happened 4 d after SPS mRNA and activity had reached their maxima. These results indicate that access to substrate and transcriptional activation with increase in SPS expression might be important regulatory events of sweetening during banana fruit ripening. »

These proposals could be confirmed by analysis of recent results on *Musa* RNA-seq.

http://www.ncbi.nlm.nih.gov/protein/3237273
http://www.ncbi.nlm.nih.gov/nuccore/3237272
http://www.ncbi.nlm.nih.gov/pubmed/9431676

4. Phylogenetic and Paralogous Region Analyses

To explore the evolutionary history of the SPS gene family. We looked at the phylogenetic et paralogous relationship at different scales.

4.1. Phylogenomics System

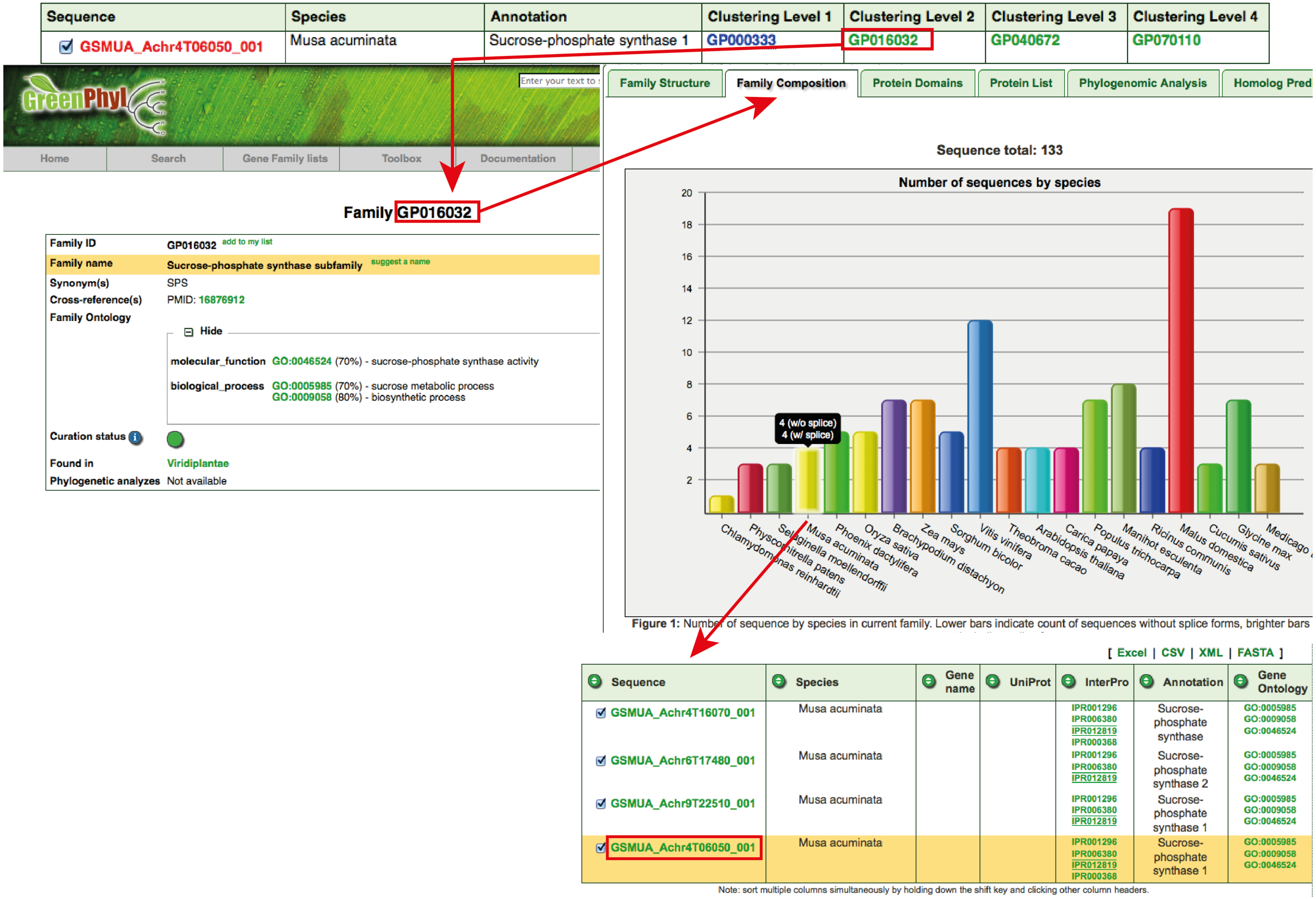
Sequence
Species
Annotation
Clustering Level 1
Clustering Level 2
Clustering Level 3
Clustering Level 4
GSMUA_Achr4T06050_001
Musa acuminata
Sucrose-phosphate synthase 1
GP000333
GP016032
GP040672
GP070110
GreenPhyl
Enter your text to
Home
Search
Gene Family lists
Toolbox
Documentation
Family Structure
Family Composition
Protein Domains
Protein List
Phylogenomic Analysis
Homolog Pred
Family GP016032
Family ID
GP016032
add to my list
Family name
Sucrose-phosphate synthase subfamily
suggest a name
Synonym(s)
SPS
Cross-reference(s)
PMID: 16876912
Family Ontology
Hide
molecular_function GO:0046524 (70%) - sucrose-phosphate synthase activity
biological_process GO:0005985 (70%) - sucrose metabolic process
GO:0009058 (80%) - biosynthetic process
Curation status
Found in
Viridiplantae
Phylogenetic analyzes
Not available
Sequence total: 133
Number of sequences by species
4 (w/o splice)
4 (w/ splice)
Chlamydomonas reinhardtii
Physcomitrella patens
Selaginella moellendorffii
Musa acuminata
Phoenix dactylifera
Oryza sativa
Brachypodium distachyon
Zea mays
Sorghum bicolor
Vitis vinifera
Theobroma cacao
Arabidopsis thaliana
Carica papaya
Populus trichocarpa
Manihot esculenta
Ricinus communis
Malus domestica
Cucumis sativus
Glycine max
Figure 1: Number of sequence by species in current family. Lower bars indicate count of sequences without splice forms, brighter bars
[Excel | CSV | XML | FASTA]
Sequence
Species
Gene name
UniProt
InterPro
Annotation
Gene Ontology
GSMUA_Achr4T16070_001
Musa acuminata
IPR001296
IPR006380
IPR012819
IPR000368
Sucrose-phosphate synthase
GO:0005985
GO:0009058
GO:0046524
GSMUA_Achr6T17480_001
Musa acuminata
IPR001296
IPR006380
IPR012819
Sucrose-phosphate synthase 2
GO:0005985
GO:0009058
GO:0046524
GSMUA_Achr9T22510_001
Musa acuminata
IPR001296
IPR006380
IPR012819
Sucrose-phosphate synthase 1
GO:0005985
GO:0009058
GO:0046524
GSMUA_Achr4T06050_001
Musa acuminata
IPR001296
IPR006380
IPR012819
IPR000368
Sucrose-phosphate synthase 1
GO:0005985
GO:0009058
GO:0046524
Note: sort multiple columns simultaneously by holding down the shift key and clicking other column headers.

Figure 9: Phylogenomics system (GreenphylDB)

Method

We can access to the gene families by clicking the links on gene families available from the GreenPhyl Report previously described (GSMUA_Achr4T06050_001; Figure 6), http://www.greenphyl.org/cgi-bin/sequence.cgi?p=id&seq_textid=GSMUA_Achr4T06050_001

or by searching the family name (sucrose-phosphate synthase),

http://www.greenphyl.org/cgi-bin/family.cgi?p=id&family_id=16032

or by searching the identifier of the InterPro entry of family type (IPR012819).

http://www.greenphyl.org/cgi-bin/family.cgi?p=id&family_id=333

GreenPhyl provides gene clusters at 4 levels of stringency, gene families, Interpro domains, the orthologs prediction and the BBMHs, accessible by clicking on the respective tabs. Through the clusters links, information is provided about the gene family. You can get the Gene ontologies, the distribution of the families between the species, the InterPro domains, the list of proteins, the prediction of homologs and a phylogenetic analysis.

Results

The four genes previously found (GSMUA_Achr4T16070_001, GSMUA_Achr4T06050_001, GSMUA_Achr6T17480_001 and GSMUA_Achr9T22510_001) belong to a cluster curated as Sucrose Phosphate Synthase subfamily (cluster GP016032, second level of clustering), which is a subfamily of the superfamily named Sucrose Synthase (cluster GP000333, first level of clustering). In this particular case, the phylogenetic analysis was not available for the sucrose phosphate synthase family, but available at the upper level.

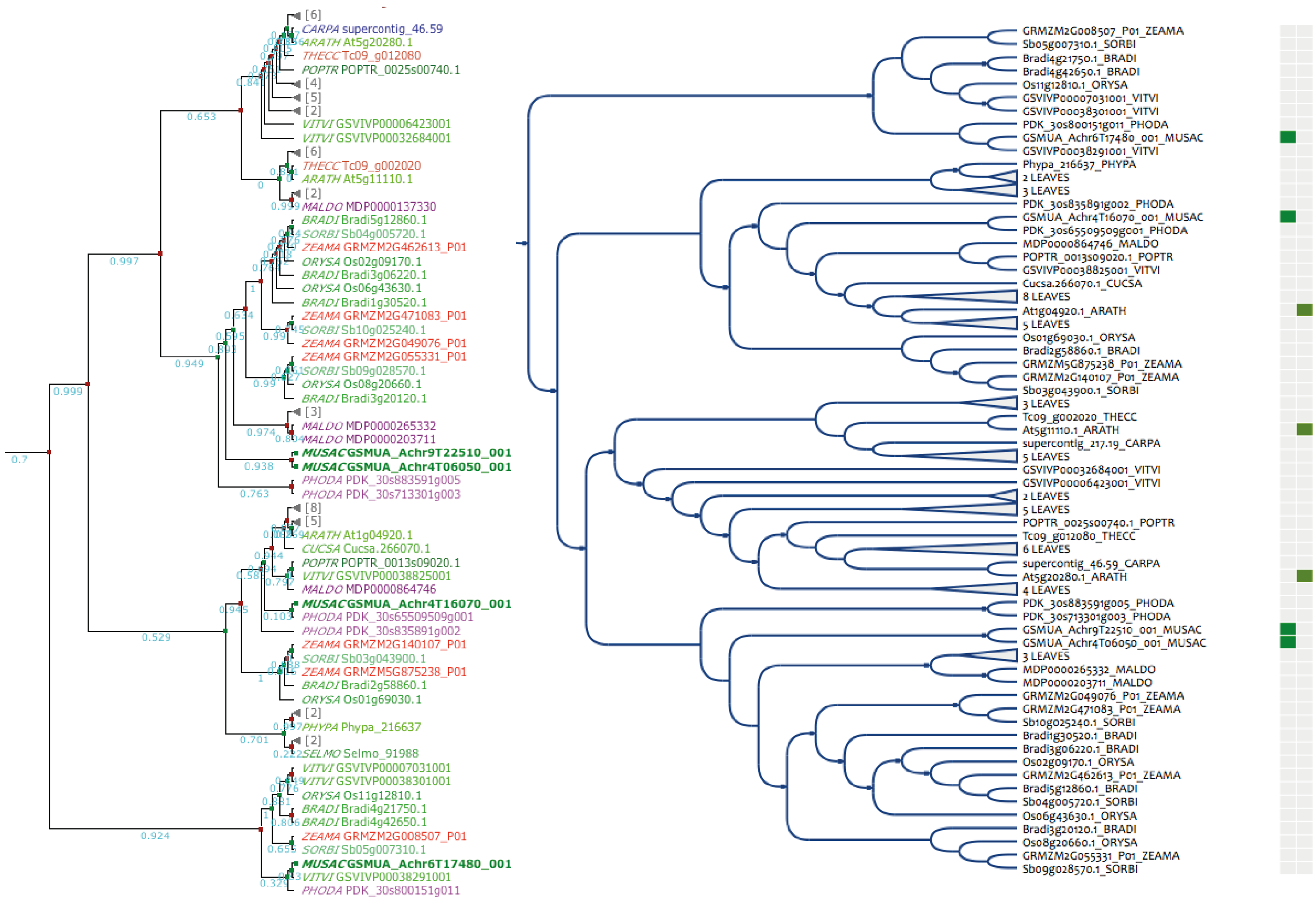
[6]
CARPA supercontig_46.59
ARATH At5g20280.1
THECC Tc09_g012080
POPTR POPTR_0025s00740.1
[4]
[5]
[2]
VITVI GSVIVP00006423001
VITVI GSVIVP00032684001
[6]
THECC Tc09_g002020
ARATH At5g11110.1
[2]
MALDO MDP0000137330
BRADI Bradi5g12860.1
SORBI Sb04g005720.1
ZEAMA GRMZM2G462613_P01
ORYSA Os02g09170.1
BRADI Bradi3g06220.1
ORYSA Os06g43630.1
BRADI Bradi1g30520.1
ZEAMA GRMZM2G471083_P01
SORBI Sb10g025240.1
ZEAMA GRMZM2G049076_P01
ZEAMA GRMZM2G055331_P01
SORBI Sb09g028570.1
ORYSA Os08g20660.1
BRADI Bradi3g20120.1
[3]
MALDO MDP0000265332
MALDO MDP0000203711
MUSAC GSMUA_Achr9T22510_001
MUSAC GSMUA_Achr4T06050_001
PHODA PDK_30s883591g005
PHODA PDK_30s713301g003
[8]
[5]
ARATH At1g04920.1
CUCSA Cucsa.266070.1
POPTR POPTR_0013s09020.1
VITVI GSVIVP00038825001
MALDO MDP0000864746
MUSAC GSMUA_Achr4T16070_001
PHODA PDK_30s65509509g001
PHODA PDK_30s835891g002
ZEAMA GRMZM2G140107_P01
SORBI Sb03g043900.1
ZEAMA GRMZM5G875238_P01
BRADI Bradi2g58860.1
ORYSA Os01g69030.1
[2]
PHYPA Phypa_216637
[2]
SELMO Selmo_91988
VITVI GSVIVP00007031001
VITVI GSVIVP00038301001
ORYSA Os11g12810.1
BRADI Bradi4g21750.1
BRADI Bradi4g42650.1
ZEAMA GRMZM2G008507_P01
SORBI Sb05g007310.1
MUSAC GSMUA_Achr6T17480_001
VITVI GSVIVP00038291001
PHODA PDK_30s800151g011
0.7
0.999
0.997
0.653
0.949
0.529
0.924
0.763
0.938
0.974
0.701
GRMZM2G008507_P01_ZEAMA
Sb05g007310.1_SORBI
Bradi4g21750.1_BRADI
Bradi4g42650.1_BRADI
Os11g12810.1_ORYSA
GSVIVP00007031001_VITVI
GSVIVP00038301001_VITVI
PDK_30s800151g011_PHODA
GSMUA_Achr6T17480_001_MUSAC
GSVIVP00038291001_VITVI
Phypa_216637_PHYPA
2 LEAVES
3 LEAVES
PDK_30s835891g002_PHODA
GSMUA_Achr4T16070_001_MUSAC
PDK_30s65509509g001_PHODA
MDP0000864746_MALDO
POPTR_0013s09020.1_POPTR
GSVIVP00038825001_VITVI
Cucsa.266070.1_CUCSA
8 LEAVES
At1g04920.1_ARATH
5 LEAVES
Os01g69030.1_ORYSA
Bradi2g58860.1_BRADI
GRMZM5G875238_P01_ZEAMA
GRMZM2G140107_P01_ZEAMA
Sb03g043900.1_SORBI
3 LEAVES
Tc09_g002020_THECC
At5g11110.1_ARATH
supercontig_217.19_CARPA
5 LEAVES
GSVIVP00032684001_VITVI
GSVIVP00006423001_VITVI
2 LEAVES
5 LEAVES
POPTR_0025s00740.1_POPTR
Tc09_g012080_THECC
6 LEAVES
supercontig_46.59_CARPA
At5g20280.1_ARATH
4 LEAVES
PDK_30s883591g005_PHODA
PDK_30s713301g003_PHODA
GSMUA_Achr9T22510_001_MUSAC
GSMUA_Achr4T06050_001_MUSAC
3 LEAVES
MDP0000265332_MALDO
MDP0000203711_MALDO
GRMZM2G049076_P01_ZEAMA
GRMZM2G471083_P01_ZEAMA
Sb10g025240.1_SORBI
Bradi1g30520.1_BRADI
Bradi3g06220.1_BRADI
Os02g09170.1_ORYSA
GRMZM2G462613_P01_ZEAMA
Bradi5g12860.1_BRADI
Sb04g005720.1_SORBI
Os06g43630.1_ORYSA
Bradi3g20120.1_BRADI
Os08g20660.1_ORYSA
GRMZM2G055331_P01_ZEAMA
Sb09g028570.1_SORBI

Figure 10: Phylogenetic trees (GreenphylDB)

Method

The phylogenetic tree of this family is accessible from the “Phylogenomic Analysis” tab and then the button “View gene tree with APTX”.

An alternate visualization is proposed through the button “View gene tree with IntreeGreat”. InTreeGreat is developed in the context of the SouthGreen platform.

The *Musa* SPS polypeptides were flagged with green squares whereas Arabidopsis polypeptides were flagged with avocat squares.

Results

We noticed in this phylogeny that two of our genes of interest are really close together: it appeared that GSMUA_Achr9G22510_001 and GSMUA_Achr4G06050_001 were the result of a recent duplication.

4.2.Phylogeny Refinement

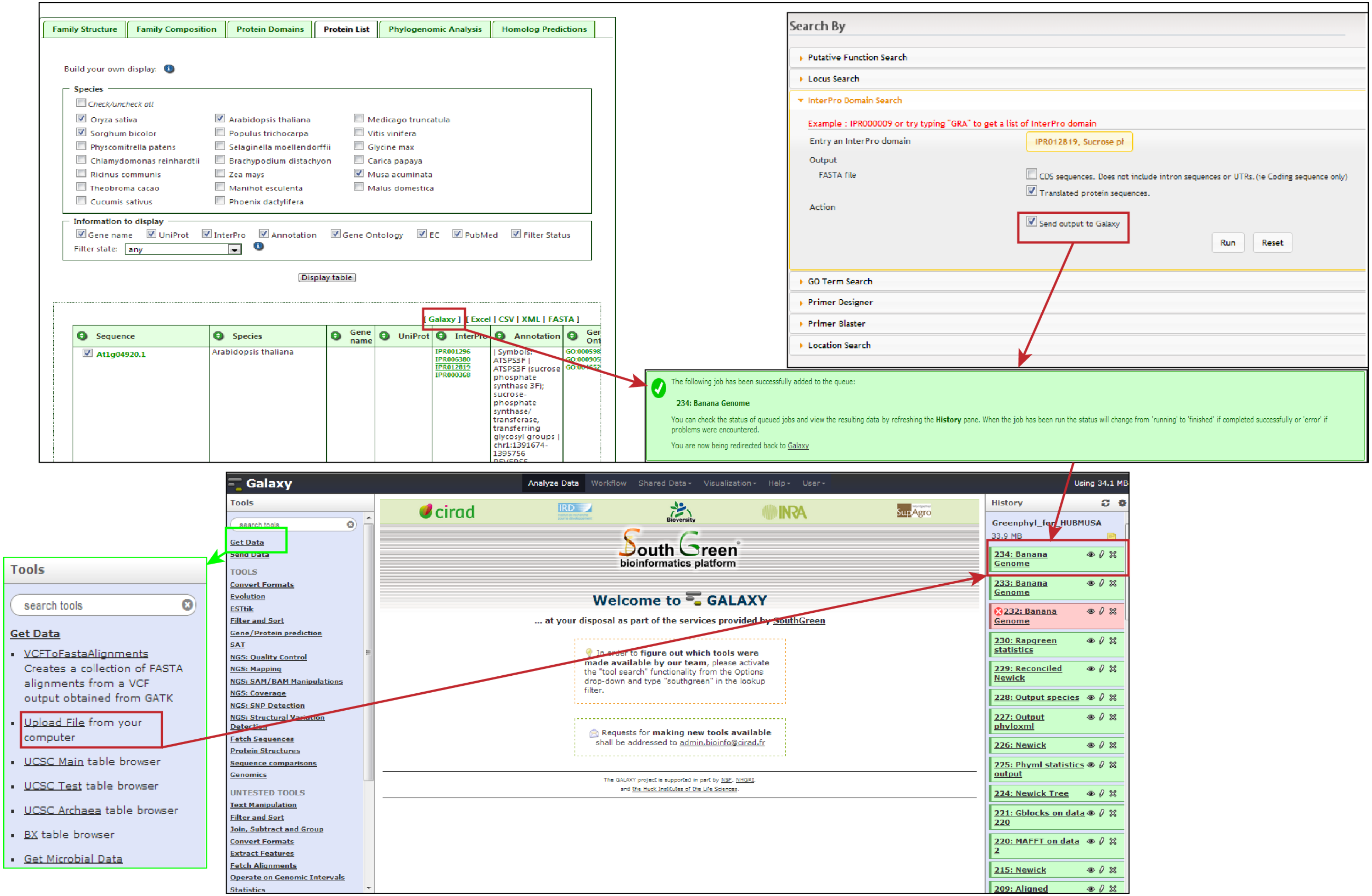
Family Structure
Family Composition
Protein Domains
Protein List
Phylogenomic Analysis
Homolog Predictions
Build your own display:
Species
Check/uncheck all
Oryza sativa
Sorghum bicolor
Physcomitrella patens
Chlamydomonas reinhardtii
Ricinus communis
Theobroma cacao
Cucumis sativus
Arabidopsis thaliana
Populus trichocarpa
Selaginella moellendorffii
Brachypodium distachyon
Zea mays
Manihot esculenta
Phoenix dactylifera
Medicago truncatula
Vitis vinifera
Glycine max
Carica papaya
Musa acuminata
Malus domestica
Information to display
Gene name
UniProt
InterPro
Annotation
Gene Ontology
EC
PubMed
Filter Status
Filter state:
any
Display table
Galaxy] [Excel | CSV | XML | FASTA]
Sequence
Species
Gene name
UniProt
Annotation
At1g04920.1
Arabidopsis thaliana
Search By
Putative Function Search
Locus Search
InterPro Domain Search
Example : IPR000009 or try typing "GRA" to get a list of InterPro domain
Entry an InterPro domain
IPR012819, Sucrose pl
Output
FASTA file
CDS sequences. Does not include intron sequences or UTRs. (ie Coding sequence only)
Translated protein sequences.
Action
Send output to Galaxy
Run
Reset
GO Term Search
Primer Designer
Primer Blaster
Location Search
The following job has been successfully added to the queue:
234: Banana Genome
You are now being redirected back to Galaxy
Galaxy
Analyze Data
Workflow
Shared Data
Visualization
Help
User
Tools
History
Greenphyl_for_HUBMUSA
234: Banana Genome
233: Banana Genome
232: Banana Genome
230: Rapgreen statistics
229: Reconciled Newick
228: Output species
227: Output phyloxml
226: Newick
225: Phyml statistics output
224: Newick Tree
221: Gblocks on data 220
220: MAFFT on data 2
215: Newick
209: Aligned
South Green
bioinformatics platform
Welcome to GALAXY
... at your disposal as part of the services provided by SouthGreen
Get Data
Send Data
TOOLS
Convert Formats
Evolution
ESTtik
Filter and Sort
Gene/Protein prediction
SAT
NGS: Quality Control
NGS: Mapping
NGS: SAM/BAM Manipulations
NGS: Coverage
NGS: SNP Detection
Fetch Sequences
Protein Structures
Sequence comparisons
Genomics
UNTESTED TOOLS
Text Manipulation
Filter and Sort
Join, Subtract and Group
Convert Formats
Extract Features
Fetch Alignments
Operate on Genomic Intervals
Statistics
Tools
search tools
Get Data
VCFToFastaAlignments
Creates a collection of FASTA alignments from a VCF output obtained from GATK
Upload File from your computer
UCSC Main table browser
UCSC Test table browser
UCSC Archaea table browser
BX table browser
Get Microbial Data

Figure 11: Upload data to the workflow manager (Galaxy)

Method

As the SPS genes have been manually annotated, we wanted to study if the phylogeny would be different. We performed a new phylogenetic analysis via an in-house Galaxy Workflow corresponding to the one implemented in GreenPhylDB.

http://gohelle.cirad.fr/galaxy/

Data uploading to Galaxy can be made from the Advanced Search tool of the Banana Genome Hub or from the Protein List tab in GreenPhylDB, after choosing species. You can also upload our own data through the uploading tool of Galaxy. Once the data uploaded, they will appear in green on the right of the screen. They will be available with a number that can be used by a Galaxy tool. Even if you are not logged in, you can use the Galaxy tools accessible in the left panel of the screen.

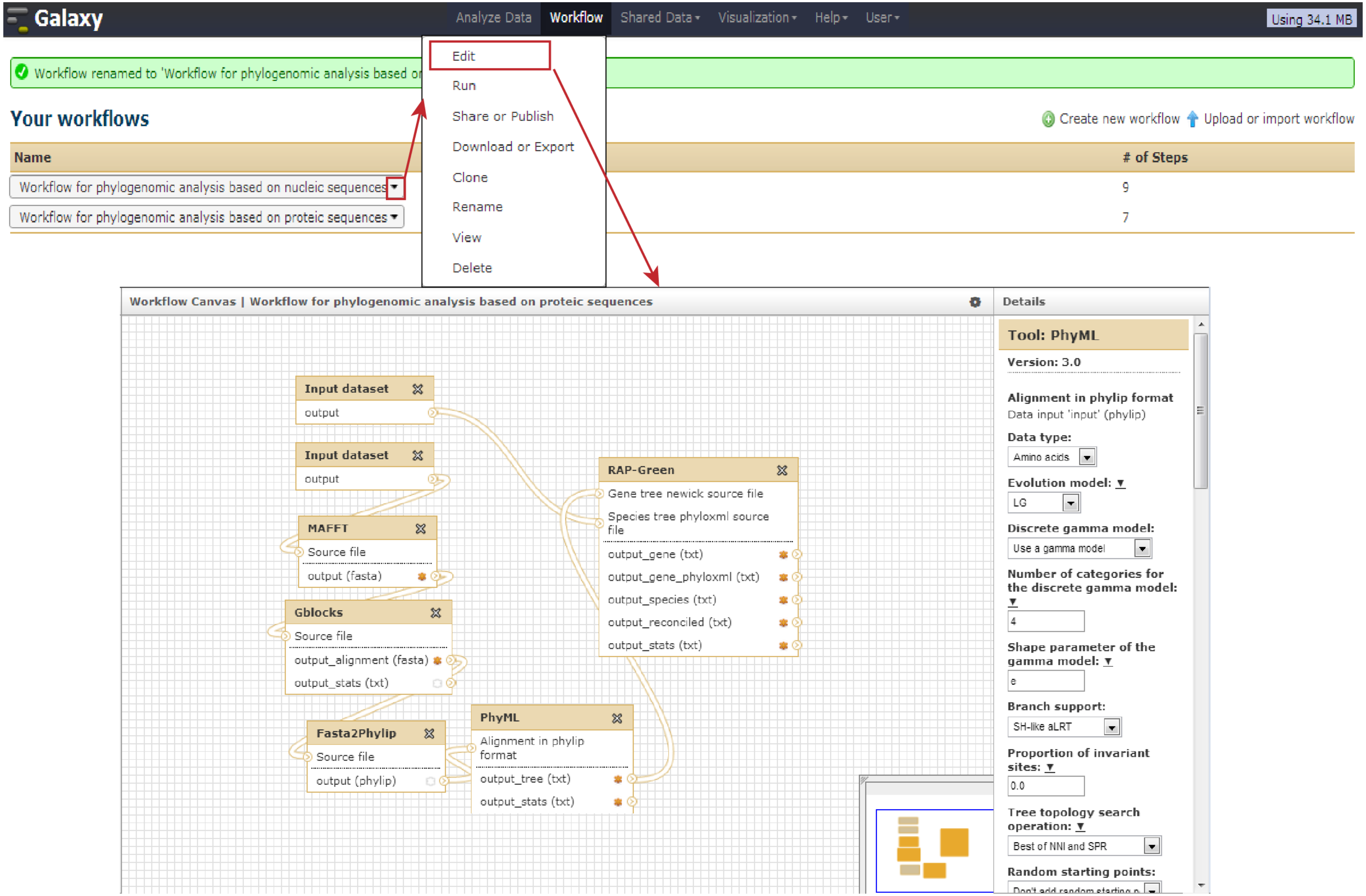

Galaxy
Analyze Data
Workflow
Shared Data
Visualization
Help
User
Using 34.1 MB
Edit
Run
Share or Publish
Download or Export
Clone
Rename
View
Delete
Workflow renamed to 'Workflow for phylogenomic analysis based o
Your workflows
Create new workflow
Upload or import workflow
Name
# of Steps
Workflow for phylogenomic analysis based on nucleic sequences
9
Workflow for phylogenomic analysis based on proteic sequences
7
Workflow Canvas | Workflow for phylogenomic analysis based on proteic sequences
Details
Input dataset
output
Input dataset
output
MAFFT
Source file
output (fasta)
Gblocks
Source file
output_alignment (fasta)
output_stats (txt)
Fasta2Phylip
Source file
output (phylip)
RAP-Green
Gene tree newick source file
Species tree phyloxml source file
output_gene (txt)
output_gene_phyloxml (txt)
output_species (txt)
output_reconciled (txt)
output_stats (txt)
PhyML
Alignment in phylip format
output_tree (txt)
output_stats (txt)
Tool: PhyML
Version: 3.0
Alignment in phylip format
Data input 'input' (phylip)
Data type:
Amino acids
Evolution model:
LG
Discrete gamma model:
Use a gamma model
Number of categories for the discrete gamma model:
4
Shape parameter of the gamma model:
e
Branch support:
SH-like aLRT
Proportion of invariant sites:
0.0
Tree topology search operation:
Best of NNI and SPR
Random starting points:

Figure 12: Editing a workflow (Galaxy)

Method

For users who have an account, the workflows are available on the Workflows tab. For those who do not have one, the workflows will not be available, but the tools are still accessible. You can apply for a account in the computer center section of the SouthGreen Web site.

http://gohelle.cirad.fr/cluster/compte_galaxy.php

The workflows can be modified in the edit section of the workflow menu accessible by clicking on the arrow next to the workflow's name. The different steps are visible in the central part of the screen. The parameters can be modified for each step in the Details panel.

Results

The workflow "GreenPhyl Phylogenomic analysis workflow (nucleic)" displayed in this example is accessible in the Shared Data / Published Workflows menu of the Galaxy web site.

http://gohelle.cirad.fr/galaxy/workflow/list_published

The protein version is also avalaible ("GreenPhyl Phylogenomic analysis workflow").

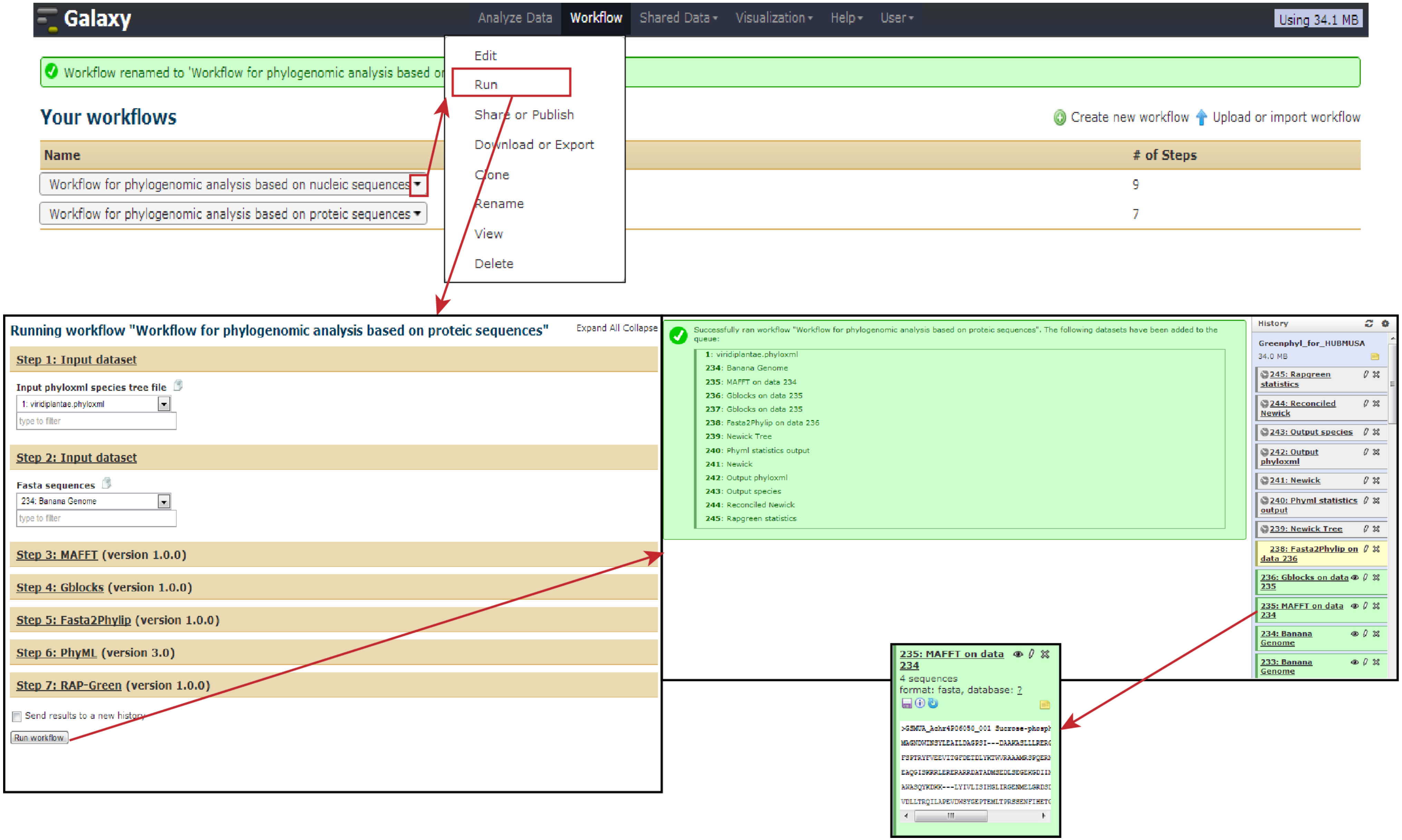
Galaxy
Analyze Data
Workflow
Shared Data
Visualization
Help
User
Using 34.1 MB
Workflow renamed to 'Workflow for phylogenomic analysis based o
Edit
Run
Share or Publish
Download or Export
Clone
Rename
View
Delete
Your workflows
Create new workflow
Upload or import workflow
Name
# of Steps
Workflow for phylogenomic analysis based on nucleic sequences
9
Workflow for phylogenomic analysis based on proteic sequences
7
Running workflow "Workflow for phylogenomic analysis based on proteic sequences"
Expand All Collapse
Step 1: Input dataset
Input phyloxml species tree file
1: viridiplantae.phyloxml
type to filter
Step 2: Input dataset
Fasta sequences
234: Banana Genome
type to filter
Step 3: MAFFT (version 1.0.0)
Step 4: Gblocks (version 1.0.0)
Step 5: Fasta2Phylip (version 1.0.0)
Step 6: PhyML (version 3.0)
Step 7: RAP-Green (version 1.0.0)
Send results to a new history
Run workflow
Successfully ran workflow "Workflow for phylogenomic analysis based on proteic sequences". The following datasets have been added to the queue:
1: viridiplantae.phyloxml
234: Banana Genome
235: MAFFT on data 234
236: Gblocks on data 235
237: Gblocks on data 235
238: Fasta2Phylip on data 236
239: Newick Tree
240: Phyml statistics output
241: Newick
242: Output phyloxml
243: Output species
244: Reconciled Newick
245: Rapgreen statistics
History
Greenphyl_for_HUBMUSA
34.0 MB
245: Rapgreen statistics
244: Reconciled Newick
243: Output species
242: Output phyloxml
241: Newick
240: Phyml statistics output
239: Newick Tree
238: Fasta2Phylip on data 236
236: Gblocks on data 235
235: MAFFT on data 234
234: Banana Genome
233: Banana Genome
235: MAFFT on data 234
4 sequences
format: fasta, database: ?

Figure 13: Running a workflow (Galaxy)

<u>Method</u>

The workflow is launched from the run section. Choose the entry data and then click on the “run workflow” button. The steps will be shown on the history, in grey while they are waiting and in yellow while process. The workflow finishes running when all the steps are green. If an error occurs, the steps will be red. You can visualize and download result files of each step by clicking on it.

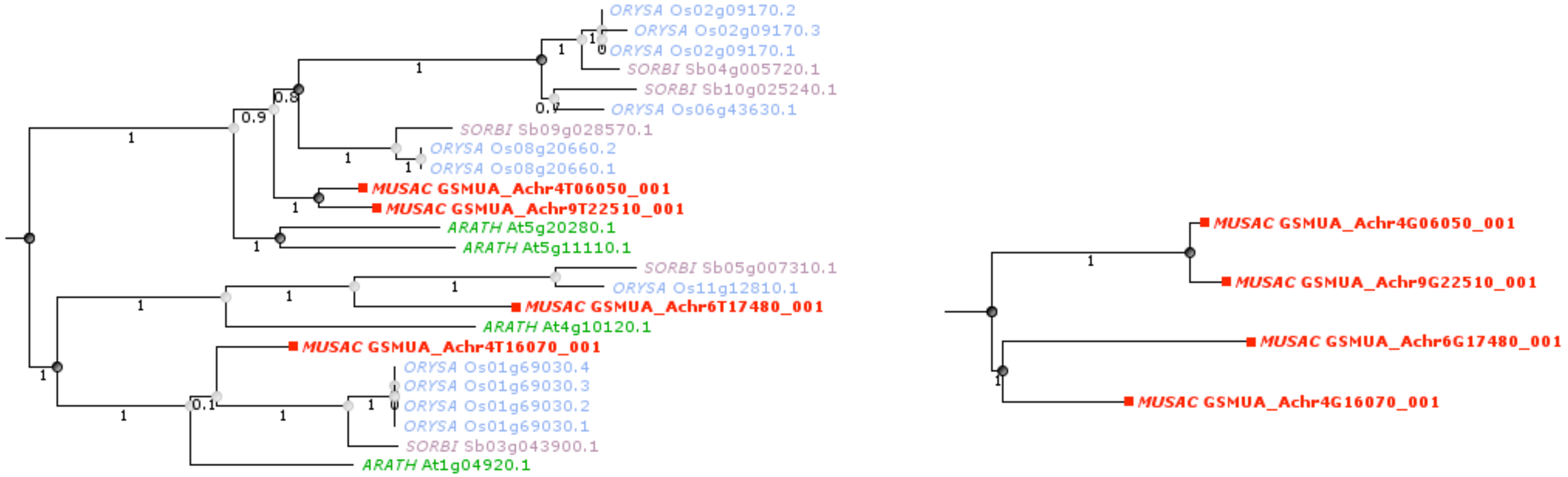

Figure 14: Phylogenetic trees (Galaxy)

<u>Results</u>

For the protein analysis, species considered are Oryza sativa (ORYSA), Sorghum bicolor (SORBI), Arabidopsis thaliana (ARATH) and Musa acuminate (MUSAC). We ran the phylogeny on fewer species and the phylogeny did not change significantly. The genes GSMUA_Achr9G22510_001 and GSMUA_Achr4G06050_001 were still very close together.

For the nucleotide analysis, only *Musa* was considered. The results are consistent; the duplication between the genes GSMUA_Achr9G22510_001 and GSMUA_Achr4G06050_001 is very more recent than between the other genes of the family in *Musa*.

4.3. Synteny of Paralogous Musa Regions

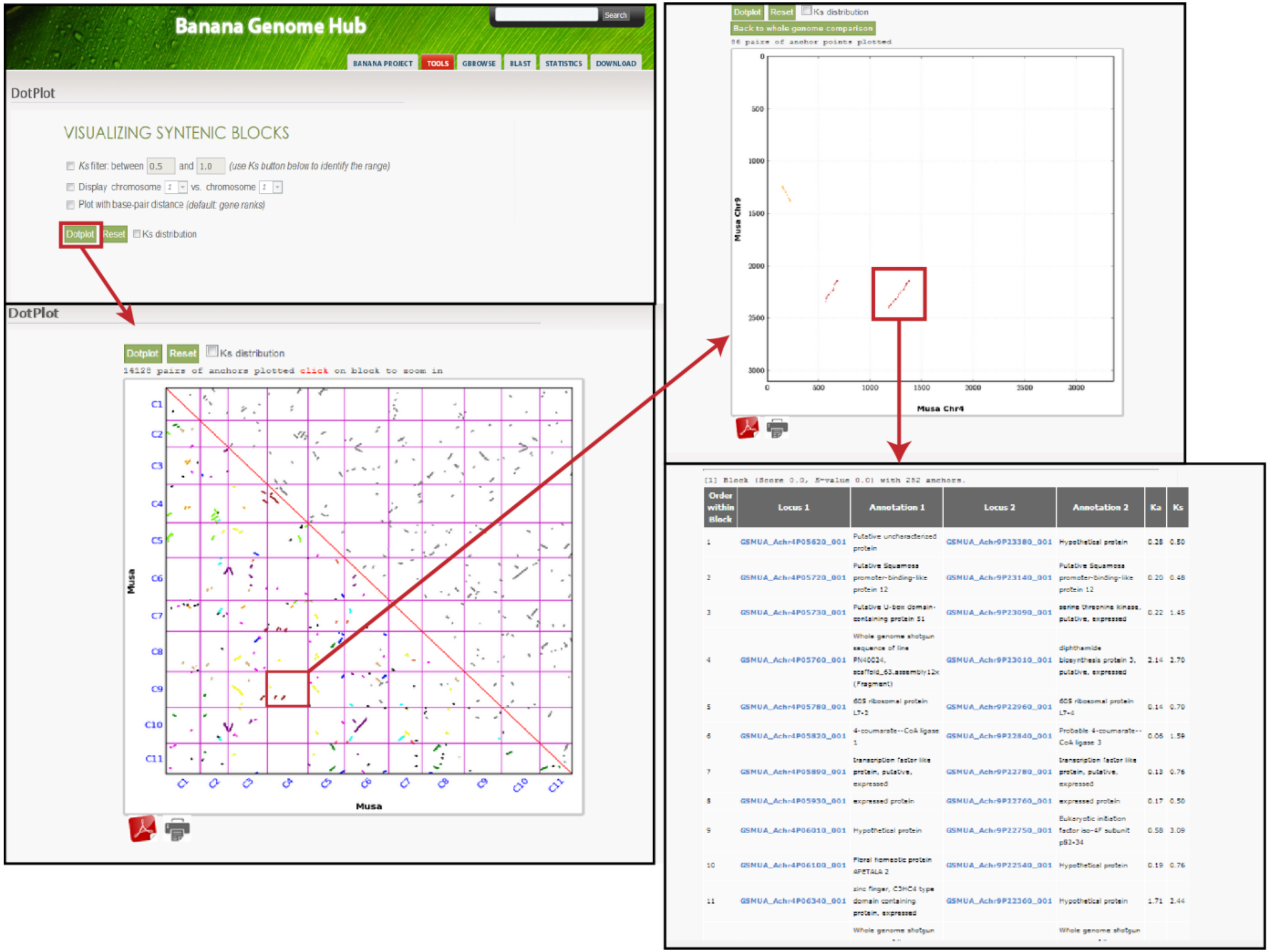

Banana Genome Hub
Search
BANANA PROJECT
TOOLS
GBROWSE
BLAST
STATISTICS
DOWNLOAD
DotPlot
VISUALIZING SYNTENIC BLOCKS
Ks filter: between 0.5 and 1.0 (use Ks button below to identify the range)
Display chromosome 1 vs. chromosome 1
Plot with base-pair distance (default: gene ranks)
Dotplot
Reset
Ks distribution
DotPlot
Dotplot
Reset
Ks distribution
C1
C2
C3
C4
C5
C6
C7
C8
C9
C10
C11
Musa
Musa
Dotplot
Reset
Ks distribution
Back to whole genome comparison
Musa Chr9
Musa Chr4
Order within Block
Locus 1
Annotation 1
Locus 2
Annotation 2
Ka
Ks

Figure 15: Dotplot of paralogous *Musa* regions coloured according to Musa ancestral blocks (PGDD)

Method

We then tried to determine if the high similarity of these genes was due to block duplication in *Musa*. For that, we used the Plant Genome Duplication Database (PGDD) system that provides in our case, paralogous regions between *Musa* chromosomes, coloured according to *Musa* ancestral blocks. The hypothesis is that these paralogous regions come from whole genome duplications (WGD).

PGDD, is available from the DotPlot section of the "Tools" tab ("Syntenic Dotplot" link).

http://banana-genome.cirad.fr/dotplot

To examine the synteny, we clicked on the Dotplot button without giving any parameters. You can zoom afterwards on a square of the dot plot to visualize the Dotplot between two chromosomes by clicking on a square in the global plot or by selecting the "Display chromosome A vs chromosome B" option. The scale then represents the gene rank. By clicking on a syntenic region, you get the list of co-localized paralogs with the values of Ks and Ka.

Results

We studied the microsynteny between the chromosomes 4 and 9 in order to conclude if the genes GSMUA_Achr9G22510_001 and GSMUA_Achr4G06050_001 that appeared very similar on the phylogeny (Figure 10 and Figure 14) were issued from the same duplicated ancestral block. We noticed that the genomic regions are issued from WGD event of the ancestral block 4 (**Figure 1**), but our two genes are not listed as syntenic in this table. Further studies will be required to confirm whether or not the duplication between GSMUA_Achr9G22510_001 and GSMUA_Achr4G06050_001 is linked to a WGD event.
