# Supplementary material for: The Banana Genome Hub
Source: Database (Oxford). 2013 May 23;2013:bat035. doi: 10.1093/database/bat035 (PMC3662865; doi:10.1093/database/bat035)
Supplement: Supplementary Data [file supp_bat035_suppl_data.zip › Supplementary File 2.pdf]

Case study of the Cellulose synthase (CesA) and cellulose synthase-like (Csl) superfamily

In this document, we provide an overview of the characterization of the cellulose synthase family using the Banana Genome Hub.

To identify Musa CESA and CSL sequences, the Musa proteome was searched using the protein combination tool of GreenPhylDB (http://www.greenphyl.org/cgi-bin/ipr2genomes.cgi) with InterPro domain IPR005150 corresponding to the cellulose synthase family (CESA, CSLB, CSLD-J) and IPR001173 corresponding to the Glycosyl transferase family 2 (CSLA, CSLC).

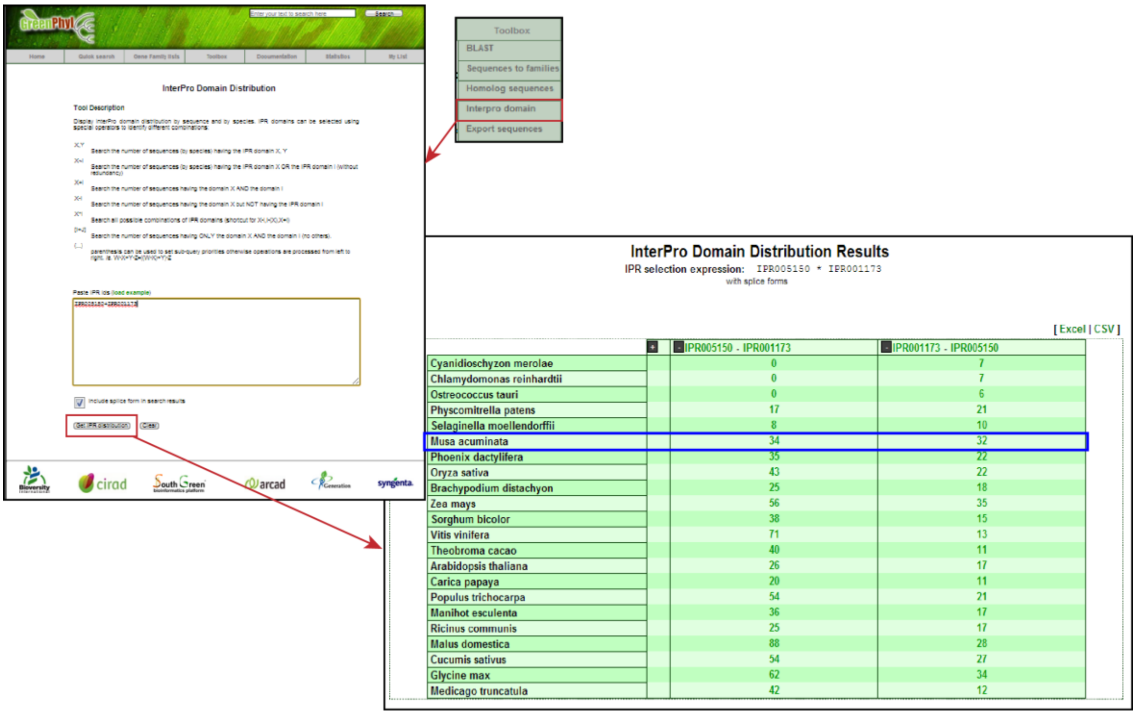

Figure 1 - Interpro domain research

The search using IPR005150 identified 34 sequences that clustered within the "Cellulose synthase" family in GreenPhylDB. The search using IPR001173 in GreenPhylDB identified 32 sequences of which 26 clustered within the "Putative glycosyl transferase" family that comprised CSLA and CSLC homologs from other species.

The sequences have also been identified through their function with pathway tools. (http://banana-genome.cirad.fr/musacyc)

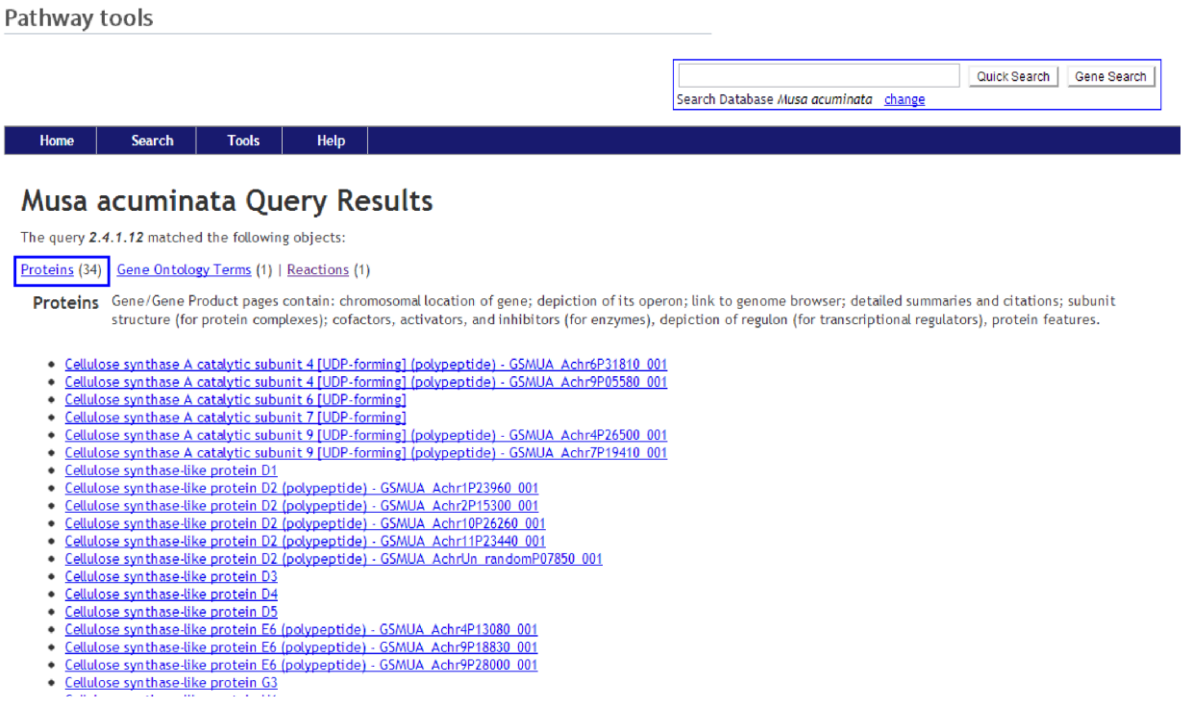

Figure 2 - Search by function on MusaCyc

Predicted enzymes with the associated E.C. number E.C. 2.4.1.12 for cellulose synthase function that were retrieved from MusaCyc(Fig.2), corresponded to the same set of 34 sequences.

Homologous sequences from the rice, sorghum, Arabidopsis and grapevine genomes were retrieved from GreenPhylDB (http://www.greenphyl.org/cgi-bin/family.cgi?p=id&family_id=73#tab-famstruct) and were assigned to the different subfamilies following published data (Fig3.)

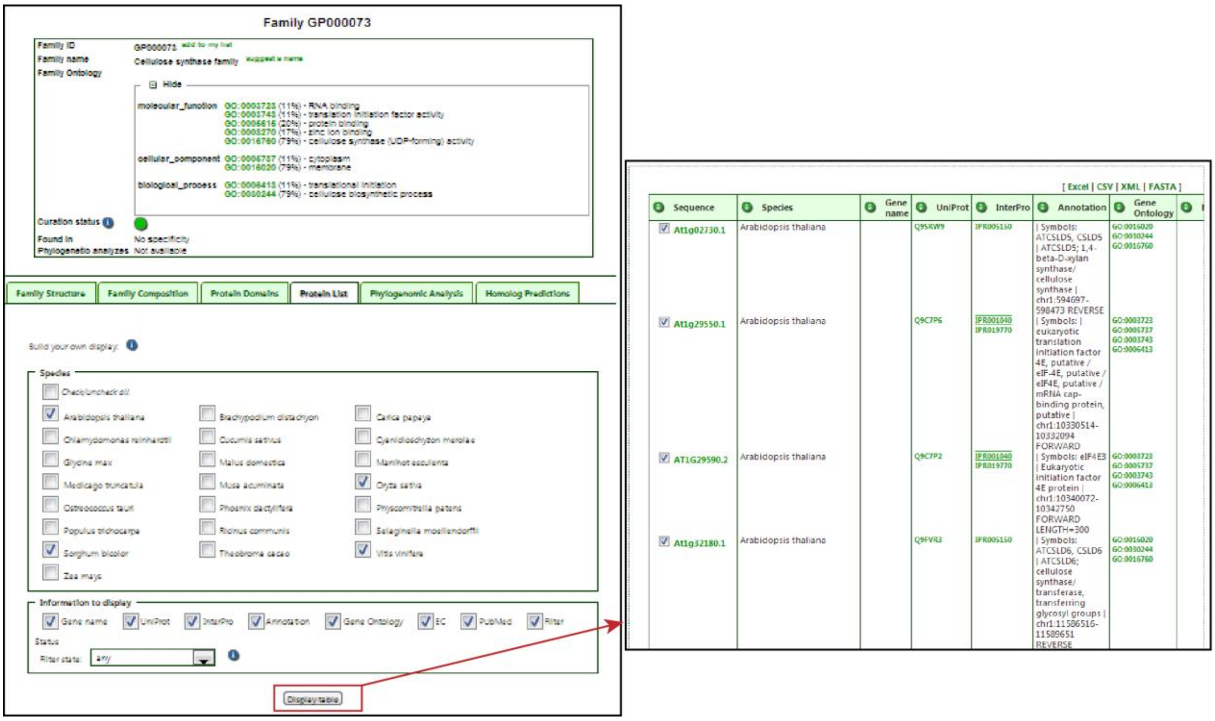

Figure 3 - Homologos sequences from rice, sorghum, Arabidopsis and grapevine genomes on GreenPhylDB

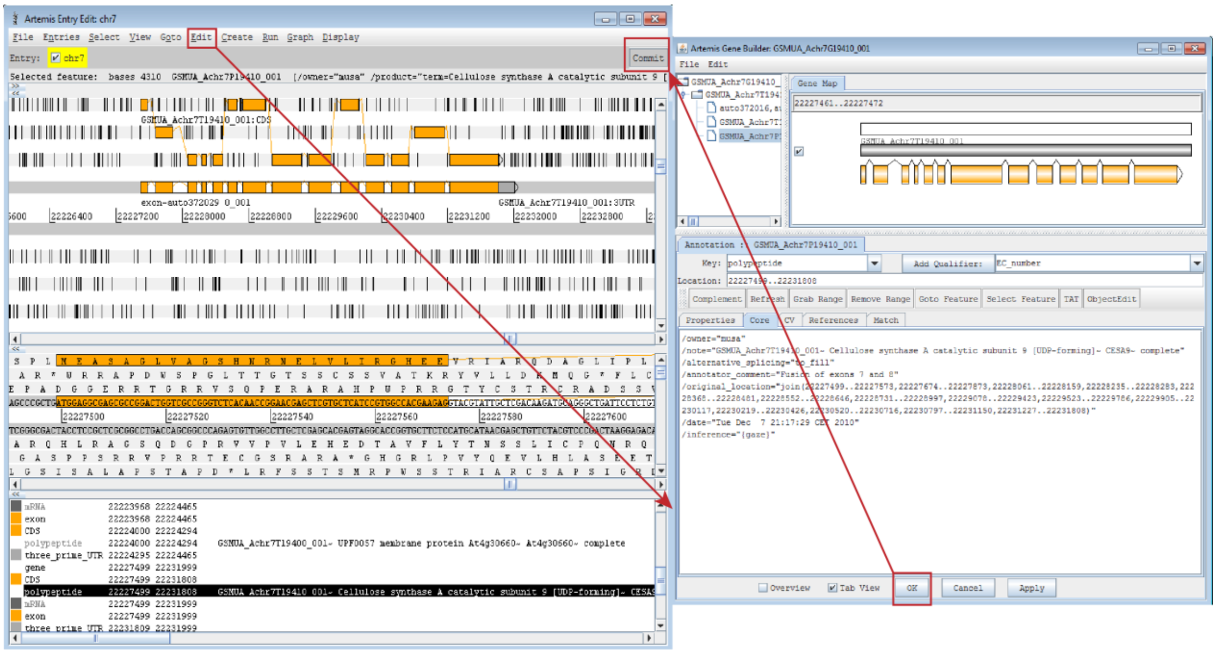

Figure 4 - Manual Curation with Artemis

Manual curation of the compiled 60 *Musa* genes (Fig. 4) was performed using information from different *de novo* prediction programs, expression data and BLASTP data available on the banana GBrowse. This resulted in a final set of 59 genes.
